# Visible-Light-Mediated Aerobic α-Oxygenation of Tetrahydroisoquinolines and Isoindolines Without External Photocatalysts

**DOI:** 10.3390/molecules30030743

**Published:** 2025-02-06

**Authors:** Taiqiang Ye, Yuzheng Li, Feng Zhao, Aorou Song, Zhaoxia Zhong, Shenpeng Tan, Feng Li

**Affiliations:** Key Laboratory of Molecular Pharmacology and Drug Evaluation (Ministry of Education), Collaborative Innovation Center of Advanced Drug Delivery System and Biotech Drugs in Universities of Shandong, School of Pharmacy, Yantai University, Yantai 264005, China; yetaiqiang@s.ytu.edu.cn (T.Y.); liyuzheng@s.ytu.edu.cn (Y.L.); songaorou@s.ytu.edu.cn (A.S.); zhongzhaoxia@s.ytu.edu.cn (Z.Z.); tanshenpeng@ytu.edu.cn (S.T.)

**Keywords:** visible light, tetrahydroisoquinolines, isoindolines, aerobic oxidation, photocatalyst-free

## Abstract

A visible-light-mediated strategy for the direct oxygenation of *N*-substituted tetrahydroisoquinolines and isoindolines to the corresponding benzo-fused lactams under clean conditions without using any external photocatalysts has been developed. The reaction was performed in the presence of a catalytic amount of base and oxygen. Mechanistic studies reveal that the reaction is initiated by the substrates themselves as photosensitizers. Additionally, BHT could be used as a buffer-like additive to improve reaction selectivity and product yield in this photo-oxidation process.

## 1. Introduction

The oxidation of hydrocarbons is one of the most fundamental and important chemical transformations in organic synthesis, and the development of green and sustainable oxidation methods has been a central goal in synthetic chemistry. Over the past decade, photocatalysis, especially visible-light-mediated photocatalysis, has emerged as an alternative to the conventional thermo-chemical process due to its mild reaction conditions and the use of nontoxic, cheap and widely abundant light as a reagent [1,2]. Photocatalysts, either metal- or organic-based, are generally employed in the photochemical strategy to convert energy from visible light into the reactants of interest, as most organic molecules do not absorb in the visible region by themselves [3,4]. On the other hand, direct excitation of organic compounds or reactive species (e.g., electron donor–acceptor (EDA) complexes) with the ability to absorb visible light to trigger a photochemical process for organic transformations without the use of photocatalysts is also of growing interest because of its economic and synthetic value [5,6].

**Figure 1:**
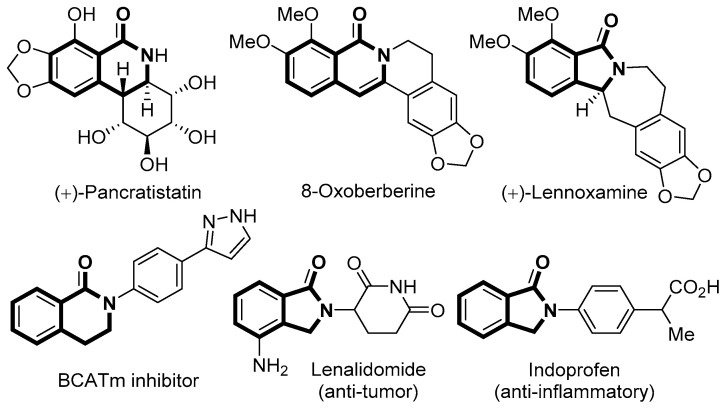
Examples of natural products and pharmaceuticals containing an isoquinolinone and isoindolinone scaffold.

The direct oxygenation of the α-methylene of tetrahydroisoquinoline and isoindoline derivatives has been extensively studied in recent years [7,8,9,10,11,12], because the resulting heterocyclic frameworks such as isoquinolinones and isoindolinones are important structural motifs of many natural alkaloids, biologically active compounds and pharmaceuticals [13,14,15,16,17,18] (Figure 1). Even though the generation of dihydroisoquinolones from tetrahydroisoquinolines has been observed, often as byproducts in cross-dehydrogenative-coupling reactions [19,20,21], the direct α-oxygenation of tetrahydroisoquinolines and isoindolines remains a challenge, especially in oxidation reactions with abundant and clean oxygen as both an oxidant and an oxygen atom source.

**Scheme 1:**
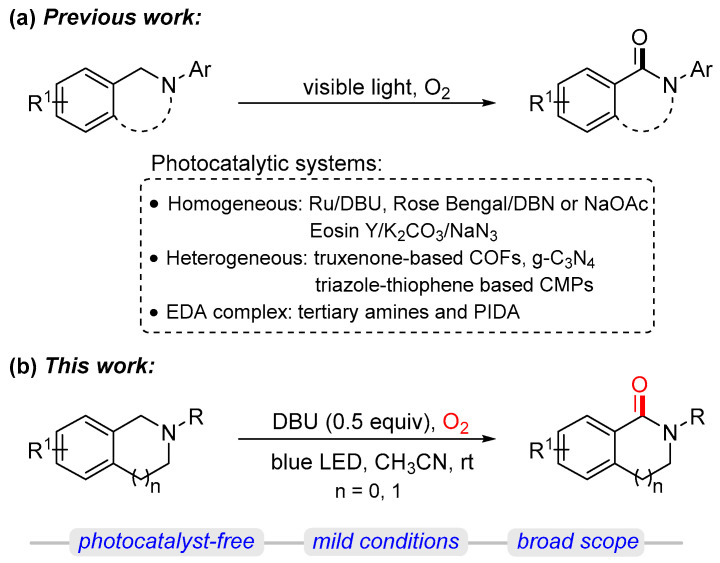
Photo-oxidation of tetrahydroisoquinolines and isoindolines.

In recent years, visible-light-mediated aerobic oxidation of tetrahydroisoquinolines and isoindolines has achieved strong priority for its environmentally friendly and sustainable features [22,23,24,25,26,27,28,29] (Scheme 1a). These photoinduced oxygenation reactions were mostly catalyzed by ruthenium-based photocatalysts [22] and organic dyes like rose bengal [23,24] and eosin Y [25] in combination with excess or stoichiometric amounts of bases such as DBU, DBN and K_2_CO_3_. In addition, some heterogeneous photocatalysts [26,27,28] based on organic polymers have been explored and applied to initiate this aerobic oxygenation reaction. Recently, Shi and colleagues [29] fulfilled the α-oxygenation of tetrahydroisoquinolines and isoindolines under photocatalyst-free conditions via the formation of photo-absorbing EDA complexes. Although there has been a plethora of strategies utilizing different photocatalysts as well as EDA complexes reported so far, the search for a green catalysis system with low costs, low toxicity and high efficiency for the photooxidation of tetrahydroisoquinolines and isoindolines is still an appealing and worthy endeavor.

Recently, we reported the *N*-bromosuccinimide (NBS)-catalyzed aerobic oxidation of benzylic C(sp^3^)–H bonds under visible light irradiation [30]. However, this catalytic system was not suitable for the oxidation of *N*-aryl-substituted tetrahydroisoquinolines and isoindolines, presumably due to the formation of iminium salts of substrates and NBS [31]. During the study, a small sample solution of *N*-phenyl tetrahydroisoquinoline was retained for detecting the reaction, and unexpectedly we observed α-oxygenation products with very low yields within a few days. This means that an auto-oxidation reaction had occurred. This interesting result prompted us to explore this transformation by utilizing the photosensitizing potential of the substrate to trigger its own oxygenation reaction instead of an external photocatalyst.

Herein, we report the photoinduced aerobic α-oxygenation of *N*-substituted tetrahydroisoquinolines and isoindolines free of either photocatalysts or organocatalysts (Scheme 1b). The presence of a catalytic amount of base can improve the yield of the reaction. Mechanistic studies reveal that the reaction is initiated by the substrates themselves as photosensitizers. Moreover, the addition of butylated hydroxytoluene (BHT) was found to enhance the product yield by reducing by-product generation in this photo-oxidative process.

## 2. Results and Discussion

In the beginning, *N*-phenyl tetrahydroisoquinoline **1a** was used as a model substrate, and the oxidation of **1a** with O_2_ in DCM was screened under natural light and visible light, respectively. We found that the highest reaction rate and full conversion of **1a** could be achieved if a 72 W blue LED was used, despite the desired dihydroisoquinolone **2a** being obtained with a 16% yield (Table 1, entry 1). To increase the yield, the reaction was next performed in the presence of DBU, as a base was commonly used to accelerate the reaction in previous reports. To our delight, the yield of **2a** increased to 50% after adding only a catalytic amount of DBU (entry 2). Several solvents, such as THF, EA, CH_3_CN and DMF, were then screened with DBU and the most effective solvent was CH_3_CN, which produced **2a** with a 74% yield (entries 3–6). The evaluation of bases indicated that similar organic bases such as DBN or TBD provided the product **2a** with moderate yields, [32] whereas the yields decreased significantly with inorganic bases such as K_2_CO_3_ and Cs_2_CO_3_ as the base (entries 7–8 and Table S1). Furthermore, as the DBU loading increased from 20 mol% to 50 mol%, the yield of **2a** was slightly improved to 80% (entry 9). A further increase in the base loading did not affect the output of the reaction (entry 10 vs. entry 9). It is worth noting that this reaction also proceeded well in air, with a yield of 85% for **2a**, albeit at a slower reaction rate (entry 11). Control experiments showed that the reaction was not successful in the absence of O_2_ and visible light (entries 12–13). In addition, the light source was also investigated, and purple light apparently accelerated the reaction, but a complex mixture was generated, with a yield of 60% for **2a** (entry 14). Yellow light gave only trace amounts of **2a** (entry 15). To verify a radical pathway involved in this reaction, radical inhibition experiments were conducted. It was found that BHT as a radical inhibitor only prolonged the reaction time and did not inhibit the reaction. In addition, we found that BHT made the reaction cleaner. Considering the antioxidant property of BHT [33,34], we hypothesized that it may interact with the oxidizing species produced in the system, thus acting with a buffer-like effect and smoothing out this oxidation reaction. Further experiments demonstrated that the addition of 0.5 equiv. of BHT significantly increased the yield of **2a** up to 95% (entry 16 and Table S1).

**Scheme 2:**
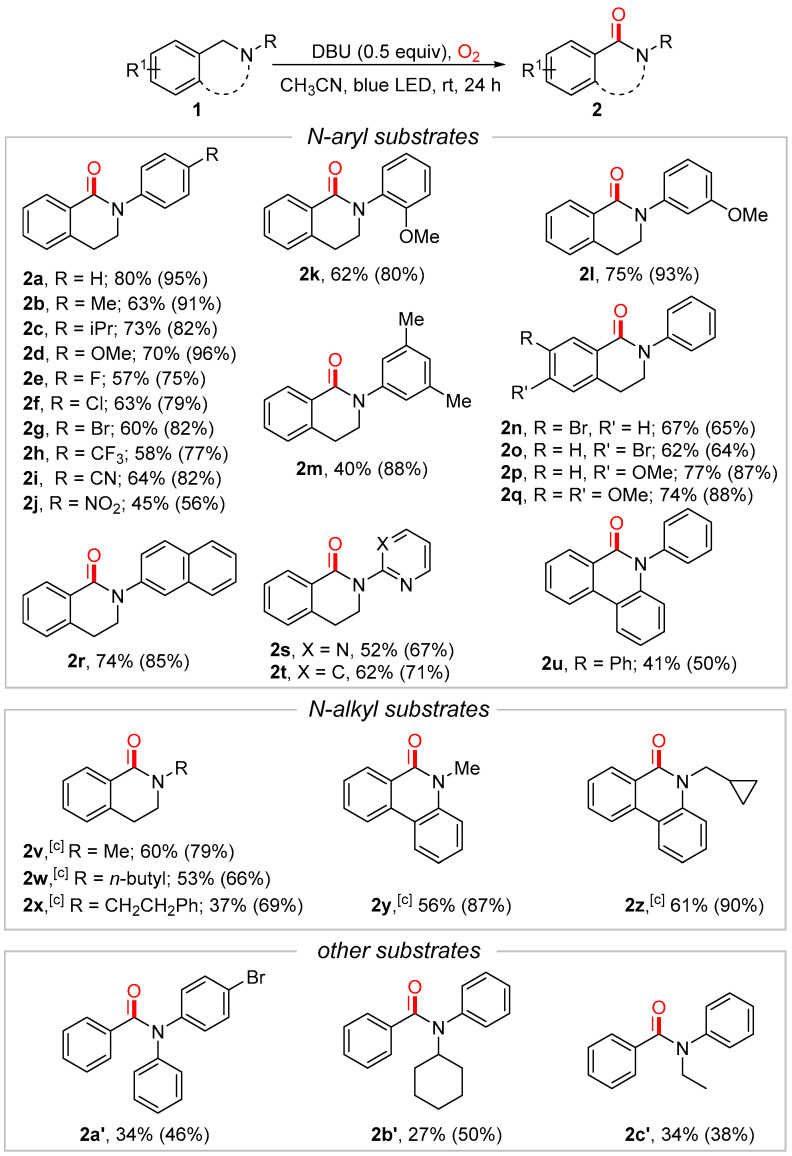
Substrate scope for tetrahydroisoquinolines ^[a][b]^. [a] Reaction conditions: **1** (0.15 mmol), DBU (0.075 mmol), CH_3_CN (3 mL), O_2_ balloon, 72 W blue LED, 24 h, rt. [b] Yields in parentheses refer to the results obtained by adding BHT (0.075 mmol) to the standard conditions. [c] A 40 W purple LED instead of a 72 W blue LED.

The ability of aerobic α-oxygenation of *N*-phenyl tetrahydroisoquinoline **1a** under DBU or DBU/BHT conditions was intriguing, and we next explored the generality of these conditions (Scheme 2). Generally, the desired oxidation products were obtained with moderate-to-good yields in the presence of only DBU, and there was an overall 10% to 20% increase in reaction yields under DBU/BHT conditions (comparison of the yields is shown in parentheses in Scheme 2). For *N*-aryl tetrahydroisoquinolines with multiple benzylic sites, high reaction selectivity was observed, and the yield was increased by 30% to 40% in the presence of BHT (**2b**: 63% vs. 91%; **2m**: 40% vs. 88%; and **2x**: 37% vs. 69%). As shown in Scheme 2, both the electron-donating and electron-withdrawing groups on the *N*-phenyl ring underwent oxidation smoothly to produce the desired products in good-to-excellent yields (**2b**–**2m**, 56–96% yields). Notably, a diminished yield was observed for **2j,** bearing a nitro group. The oxidation of *N*-(4-nitrophenyl)-tetrahydroisoquinoline always suffers from low efficiency in the single-electron transfer (SET) catalytic system [35], suggesting that the present reaction may involve a single-electron transfer process. Changing the electronic effect of the phenyl ring of tetrahydroisoquinolines had no significant influence on the reaction efficiency (**2n**–**2q**). Products **2n** and **2o** possessing a halide group showed similar results under both reaction conditions. Furthermore, *N*-naphthyl- or *N*-heteroaryl-substituted tetrahydroisoquinolines also exhibited good reactivity, leading to desired products with good yields (**2r**–**2t**, 67–85% yields). *N*-Phenyl tetrahydroisoquinoline with a fused phenyl ring also provided the desired product with a moderate yield (**2u**, 50%). The oxidation of *N*-alkyl tetrahydroisoquinolines was also conducted. The reactivity of *N*-alkyl tetrahydroisoquinolines under the standard blue light conditions was sluggish; however, when conducted under the purple light conditions, the yields of products significantly increased (**2v**–**2z**, 66–90% yields). In addition, the present protocol could be extended to open-chain benzyl amines, although only moderate yields were obtained (**2a′**–**2c′**, 38–50% yields).

**Scheme 3:**
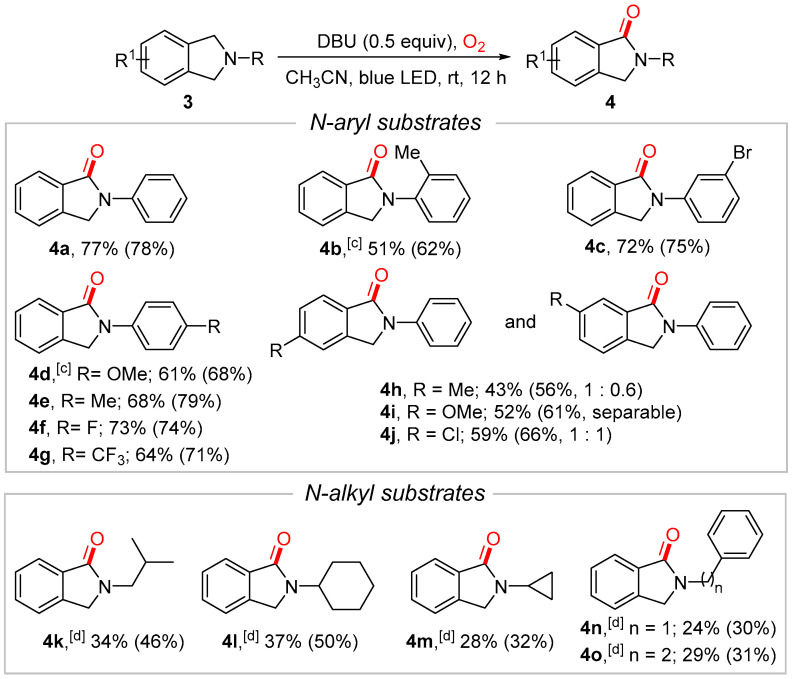
Substrate scope for isoindolines ^[a][b]^. [a] Reaction conditions: **3** (0.15 mmol), DBU (0.075 mmol), CH_3_CN (3 mL), O_2_ balloon, 72 W blue LED, 12 h, rt. [b] Yields in parentheses refer to the results obtained by adding BHT (0.075 mmol) to the standard conditions. [c] DMF as solvent. [d] 40 W purple LED instead of 72 W blue LED.

Subsequently, *N*-aryl/alkyl isoindolines were examined to further explore the generality of this reaction (Scheme 3). Different substituted *N*-aryl isoindolines were also compatible with this transformation (**4a**–**4j**, 56–79% yields). It is noteworthy that the DBU/BHT conditions did not have a significant advantage in oxidizing isoindolines, which may be because the reaction rate of isoindolines is faster than that of tetrahydroisoquinolines, and thus the inhibition of by-product formation by BHT is not strong. Nevertheless, for the methyl-containing *N*-aryl isoindolines, the addition of BHT was still superior to base-only conditions, resulting in an about 10% increase in yield (**4b**: 51% vs. 62%; **4e**: 68% vs. 79%; and **4h**: 43% vs. 56%). Furthermore, *N*-alkyl substrates including isobutyl, cyclohexyl, cyclopropyl, benzyl and phenylethyl were applied under the minorly modified standard conditions to afford **4k**–**4o** with comparably lower yields (30–50% yields).

**Scheme 4:**
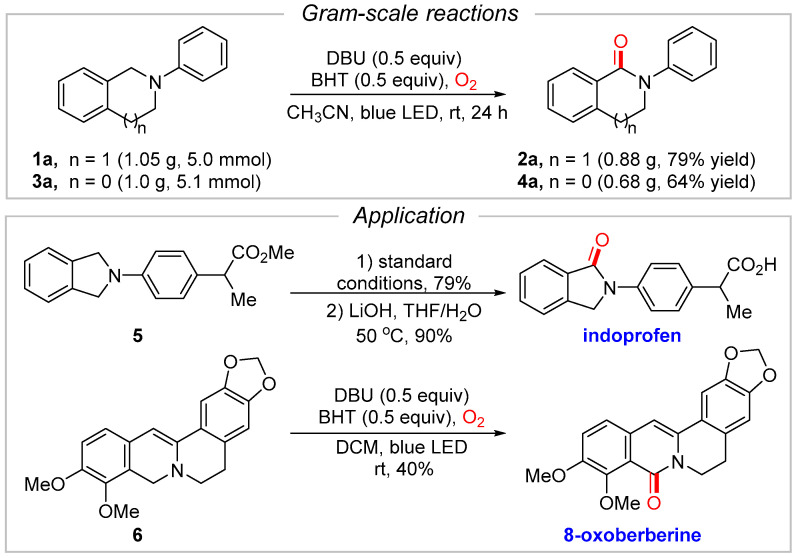
Gram-scale reactions and applications in the synthesis of indoprofen and 8-oxoberberine.

The synthetic utility of this protocol was further demonstrated by gram-scale experiments and applications in the synthesis of bioactive and medicinal compounds (Scheme 4). When the reaction of **1a** and **3a** was scaled up to 5 mmol, the corresponding products were still obtained with good yields. The present oxygenation method was also successfully applied as a key step in the synthesis of indoprofen, a known anti-inflammatory drug and platelet aggregation inhibitor [18]. In addition, we also examined the applicability of this method for the direct synthesis of natural products. 8-Oxoberberine, one of the important members of the berberine family [14], can be synthesized directly by the oxidation of **6** under the minorly modified standard conditions.

**Figure 2:**
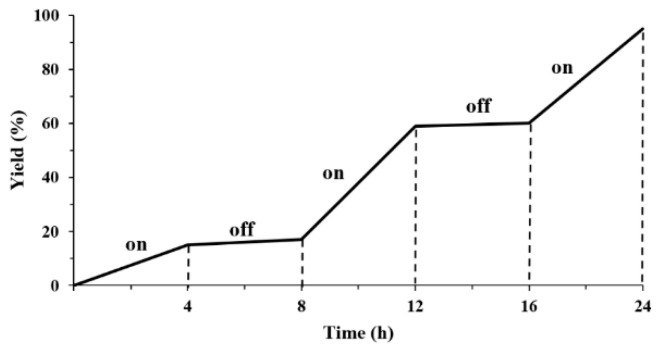
Light on-off experiments.

In order to gain insight into the reaction mechanism, several experiments were conducted. First, almost no oxygenation of **1a** occurred without O_2_ or light irradiation (Table 1, entries 12–13). Meanwhile, light on–off experiments were also conducted to prove that the reaction was completely suppressed in the absence of visible light (Figure 2). Next, the effect of different radical scavengers was studied to recognize possible intermediates and reactive oxygen species (Table 2). When TEMPO was employed, an apparent decrease in the yield was observed (Table 2, entry 1). In addition, the TEMPO-trapped intermediate was detected by ESI-HRMS (see Supporting Information for details), suggesting that a radical pathway might be involved in this transformation. The addition of benzoquinone (BQ) and CuCl_2_ to the reaction exhibited extremely lower yields, which clearly indicated the presence of the superoxide anion radical (O_2_**^−^**) and the involvement of single-electron processes in this oxidation reaction (Table 2, entries 2–3). Furthermore, almost identical reaction results were obtained when DABCO was used as a singlet oxygen (^1^O_2_) scavenger, which suggested that singlet oxygen was not involved (Table 2, entry 4).

**Figure 3:**
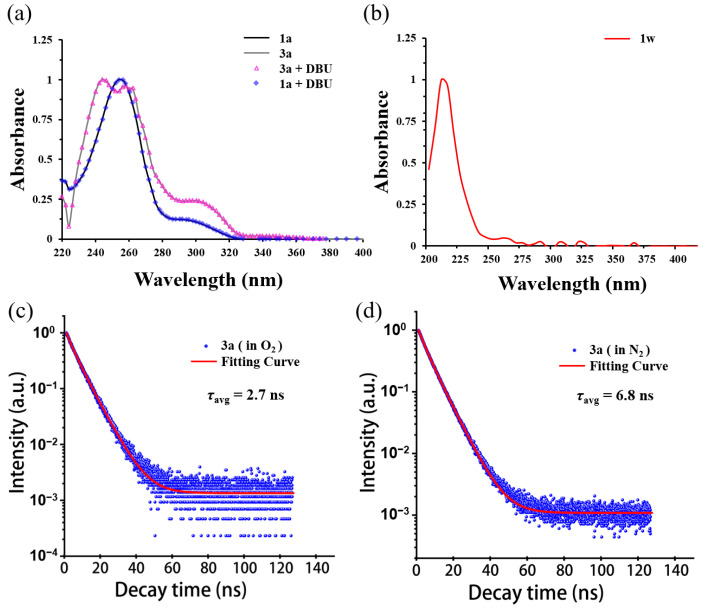
(**a**) UV/vis absorption spectra of **1a**, **3a**, **1a** + DBU and **3a** + DBU (0.2 μM) in CH_3_CN. (**b**) UV/vis absorption spectra of **1w** (0.2 μM) in CH_3_CN. (**c**) Time-dependent delayed fluorescence lifetime decay spectra of **3a** in O_2_. (**d**) Time-dependent delayed fluorescence lifetime decay spectra of **3a** in N_2_.

Considering no external photocatalysts were used in this reaction, we next focused on understanding how energy from visible light is harvested during the reaction. Thus, UV-Vis absorption spectroscopies were studied; substrates **1a** and **3a** showed strong absorption in the near-UV region, with maximum absorption wavelengths of 254 nm and 244 nm for **1a** and **3a**, respectively (Figure 3a). In addition, the absorption spectra of the mixture of **1a** and DBU or **3a** and DBU were almost unchanged compared to that of **1a** or **3a** (Figure 3a), suggesting that an EDA mechanism could be ruled out. Although no significant absorption was observed for **1a** or **3a** above 400 nm, blue LED visible light sources (*λ*_max_ = 465 nm) gave the highest yield (Table 1). We hypothesize that the emission tail of the blue LED at wavelengths shorter than the emission maximum could be sufficient to excite the substrate and trigger the photo-oxidation reaction [36]. However, for *N*-alkyl substrates such as **1w,** with absorption in the much more blue-shifted region of 210 nm (Figure 3b), purple LED light with a shorter wavelength and higher energy was more effective than blue LED (see Scheme 2 and Scheme 3).

**Scheme 5:**
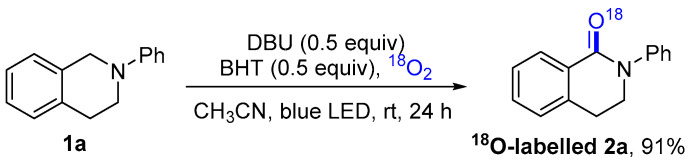
^18^O-labeling experiment.

The fluorescence quenching experiments were then investigated to demonstrate that a SET process occurred from photoexcited substrates and oxygen. As expected, the average lifetime of the excited state of **3a** in O_2_ was clearly shorter than that in N_2_ (2.7 ns vs. 6.8 ns), suggesting that O_2_ can quench the excited state of **3a** to some extent (Figure 3c,d). Moreover, the fluorescence intensity of **1a** decreased significantly with increasing concentrations of oxidizing agents such as BQ and NBS (Figure S5, see Supporting Information for details). In addition, oxygenation of **1a** in the presence of an ^18^O_2_ atmosphere resulted in a ^18^O-labeled **2a** with a 91% yield, which revealed that the amide oxygen atoms in the products were derived from oxygen (Scheme 5).

**Scheme 6:**
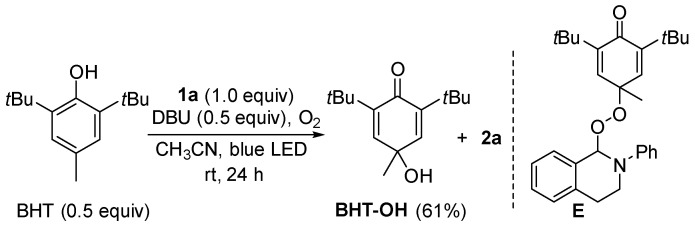
Identification of BHT-OH in the presence of BHT.

The role of BHT in this reaction was also investigated. During the study, we found that BHT was fully converted, so the major by-product that arose along with the oxidized product was isolated and finally identified as BHT-OH (Scheme 6). In addition, the ESI-HRMS analysis of the reaction mixture of **1a** revealed the presence of intermediate **E**. These results indicated that BHT was oxidized in this system and participated in the oxidation of **1a**. Therefore, the slower reaction rate and high yields in the presence of BHT should be attributed to the consumption of partially highly reactive oxygen species by BHT, thus preventing the generation of other by-products.

**Scheme 7:**
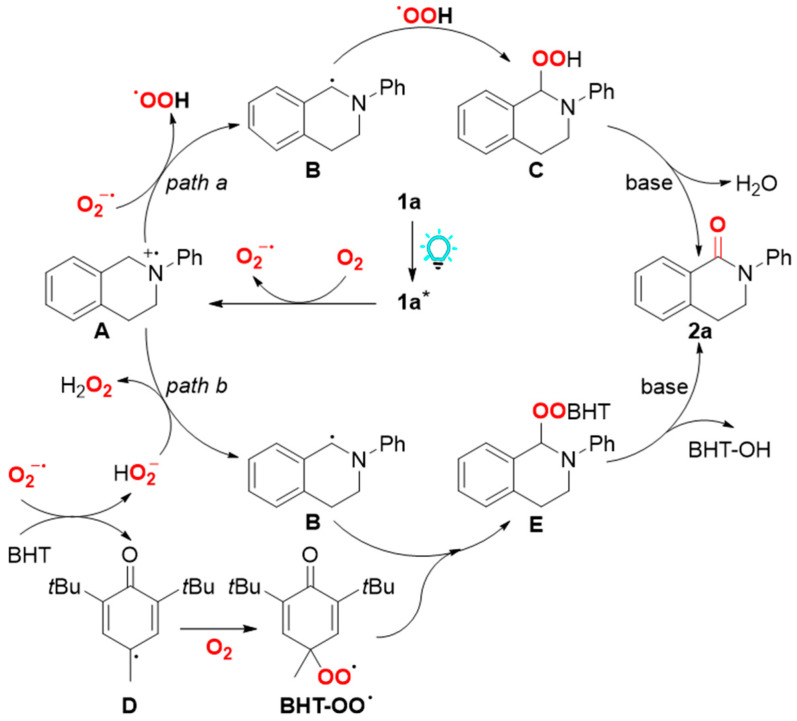
Plausible mechanism.

Based on the above studies, a plausible mechanism was proposed (Scheme 7). Firstly, **1a** was excited by the irradiation of visible light and underwent a SET process with oxygen, thus generating amine radical cation intermediate **A** and O_2_**^−^**, which both combine to give carbon-centered radical **B** via path a. Subsequently, radical **B** reacted with the peroxide radical (HOO˙) from the O_2_**^−^** to form intermediate **C**. Finally, the desired product **2a** was obtained by base-assisted H_2_O elimination in intermediate **C**. On the other hand, when BHT was added to the reaction, the active species O_2_**^−^** could also react with BHT to generate a BHT radical **D** and a hydroperoxide anion (HO_2_**^−^**). Then, radical **D** underwent radical addition with oxygen to give the BHT peroxy radical (BHT-OO˙), which further reacted with radical **B** generated from the reaction between intermediate **A** and HO_2_**^−^** to produce intermediate **E**. Base-promoted elimination in intermediate **E** would provide the desired product **2a** and BHT-OH.

## 3. Conclusions

In conclusion, a facile and efficient visible-light-mediated strategy was developed for the synthesis of dihydroisoquinolones and isoindolinones. This photocatalytic method represents an example of α-oxygenation of tetrahydroisoquinolines and isoindolines without using any photocatalyst and chemical oxidant. The reaction works well on a wide range of substrates under mild conditions. This work provides a possible manner for photochemical synthesis using one of these simple benzo-fused lactams as a photosensitizer. Further work in this regard is underway in our laboratory.

## Figures and Tables

**Table 1:** Optimization of reaction conditions ^[a]^.

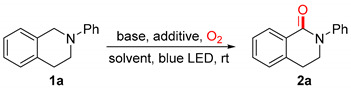				
Entry	Base (Equiv)	Solvent	Time (h)	Yield (%) ^[b]^
1	-	DCM	12	16
2	DBU (0.2)	DCM	12	50
3	DBU (0.2)	THF	12	71
4	DBU (0.2)	EA	24	61
5	DBU (0.2)	CH_3_CN	24	74
6	DBU (0.2)	DMF	24	65
7	DBN (0.2)	CH_3_CN	24	50
8	TBD (0.2)	CH_3_CN	24	46
9	DBU (0.5)	CH_3_CN	12	80
10	DBU (1.0)	CH_3_CN	12	78
11 ^[c]^	DBU (0.5)	CH_3_CN	48	85
12 ^[d]^	DBU (0.5)	CH_3_CN	12	0
13 ^[e]^	DBU (0.5)	CH_3_CN	12	trace
14 ^[f]^	DBU (0.5)	CH_3_CN	12	60
15 ^[g]^	DBU (0.5)	CH_3_CN	12	trace
16 ^[h]^	DBU (0.5)	CH_3_CN	24	95

[a] Reaction conditions: **1a** (0.15 mmol), base (0.2–1.0 equiv.), solvent (3 mL), O_2_ balloon, 72 W blue LED (450–465 nm), rt. [b] Isolated yield. [c] Under an air balloon. [d] Under a N_2_ balloon. [e] In the dark. [f] Purple light (365–375 nm). [g] Yellow light (560–575 nm). [h] BHT (0.5 equiv.) was added.

**Table 2:** Quenching experiments for oxygenation of **1a** ^[a]^.

Entry	Quencher (1 Equiv)	Yield (%) ^[b]^
1	TEMPO	44
2	BQ	19
3	CuCl_2_	10
4	DABCO	72

[a] Reaction conditions: **1a** (0.15 mmol), DBU (0.5 equiv.), quencher (0.15 mmol), CH_3_CN (3 mL), O_2_ balloon, 72 W blue LED, 24 h, rt. [b] Isolated yield.

## Data Availability

The data presented in this study are available in the article and Supplementary Materials.

## Supplementary Materials

The following supporting information can be downloaded at: https://www.mdpi.com/article/10.3390/molecules30030743/s1: Table S1 and Figures S1–S6; ^1^H and ^13^C nuclear magnetic resonance (NMR) spectra and experimental procedures. References [11,37,38,39,40,41,42,43,44] are cited in the supplementary materials.
