# Supplementary material for: Visible-Light-Mediated Aerobic α-Oxygenation of Tetrahydroisoquinolines and Isoindolines Without External Photocatalysts
Source: Molecules. 2025 Feb 6;30(3):743. doi: 10.3390/molecules30030743 (PMC11820899; doi:10.3390/molecules30030743)

Supporting information

Visible-light-mediated Aerobic α-Oxygenation of Tetrahydro isoquinolines and Isoindolines without External Photocatalysts

Taiqiang Ye,‡ Yuzheng Li,‡ Feng Zhao,* Aorou Song, Zhaoxia Zhong, Shenpeng Tan, and Feng Li*

Key Laboratory of Molecular Pharmacology and Drug Evaluation (Ministry of Education), Collaborative Innovation Center of Advanced Drug Delivery System and Biotech Drugs in Universities of Shandong, School of Pharmacy, Yantai University, Yantai 264005, China

E-mail: zhaofeng@ytu.edu.cn, lifengsnu@ytu.edu.cn.

‡ These authors contributed equally.

Contents

1. General ······ S1

2. LED Light Sources and Photoreactors in Detail ······ S2

3. Optimization of the Reaction Conditions ······ S3

4. Preparation of the Starting Materials ······ S3-S4

5. Experimental Procedures for Aerobic Oxygenation Reactions ······ S4–S24

6. Mechanistic Studies ······ S25–S31

6.1 Radical Trapping Experiments ······ S25-S27

6.2 ^{18}O Labeling Experiments ······ S27

6.3 UV-Vis Absorption and Fluorescence Properties ······ S27-S31

7. Application in the Synthesis of Indoprofen and 8-Oxoberberine ······ S32–S34

8. Reference ······ S34

9. ^{1}H NMR and ^{13}C NMR Spectra ······ S35–S85

1. General

All of the chemicals were obtained from commercial supplier and were used without further purification unless otherwise stated. All solvents were dried and distilled under nitrogen prior to use. The reactions were monitored by thin layer chromatography (TLC) analysis using silica gel GF-254 TLC plates. The compound spots were visualized using UV light. The melting points were measured using a Shanghai Shenguang Instrument Melting Point apparatus without correction. Flash column chromatography was carried out on silica gel (200–300 mesh). ^{1}H NMR (400 MHz) and ^{13}C NMR (101 MHz) spectra were referenced to Me_4Si (0 ppm), residual $CDCl_3$ (^{1}H NMR δ = 7.25 ppm, ^{13}C NMR δ = 77.16 ppm). The splitting patterns are indicated as s (singlet), d (doublet), t (triplet), q (quartet) and m (multiplet) for the ^{1}H NMR and ^{13}C NMR data. The high-resolution mass spectra (HRMS) were recorded using ESI/Q-TOF.

2. LED Light Sources and Photoreactors in Detail

The photoreactor used for reaction was bought from GeAo Chem (Figure S1). Manufacturer: Wuhan GeAo Chemical Technology Co., Ltd; Model: 72 W blue LED; Wavelength: λ = 450~465 nm; Material of the irradiation vessel: borosilicate tube (25 mL or 150 mL); Distance from the light source to the irradiation vessel: 1.5 cm (Not use any filters).

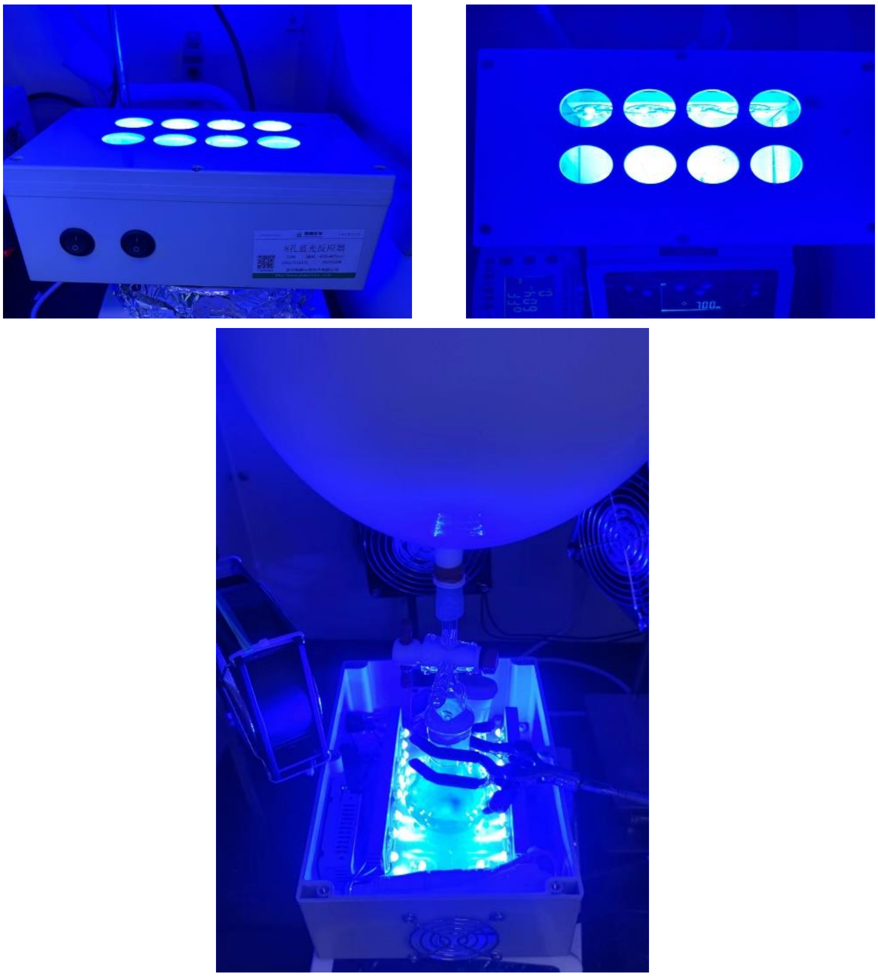

Figure S1. Photoreactor used in this research (72 W blue LED)

3. Optimization of the Reaction Conditions

Table S1. Optimization of the reaction conditions[a]

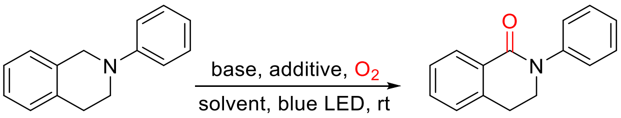

1a **2a**

entry	base (equiv)	additive (equiv)	solvent	time (h)	yield (%)[b]
1	DBU (0.5)	-	CH_3CN	12	80
2	DMAP (0.2)	-	CH_3CN	24	56
3	DABCO (0.2)	-	CH_3CN	12	53
4	K_2CO_3 (0.2)	-	CH_3CN	12	38
5	Cs_2CO_3 (0.2)	-	CH_3CN	12	35
6	LiOH (0.2)	-	CH_3CN	24	trace
7	$Ca(OH)_2$ (0.2)	-	CH_3CN	24	trace
8	DBU (0.5)	BHT (1.0)	CH_3CN	24	86
9	DBU (0.5)	BHT (0.2)	CH_3CN	24	81
10	DBU (0.5)	BHA (0.5)	CH_3CN	24	90
11	DBU (0.5)	TBHQ (0.5)	CH_3CN	24	84
12[c]	DBU (0.5)	BHT (0.5)	CH_3CN	48	70

[a]Reaction conditions: **1a** (0.15 mmol), base (0.03–0.075 mmol), additive (0.03–0.15 mmol), solvent (3 mL), O_2 balloon, 72 W blue LED, rt. [b]Isolated yield. [c]Under an air balloon. BHA: Butylated hydroxyanisole; TBHQ: *tert*-Butylhydro quinone.

4. Preparation of the Starting Materials

Unless otherwise specified, oxidation substrates were obtained from commercial suppliers. Substrates **1a-1t**,[37] **1u**,[38] **1v-1w**,[39] **1x**,[40] **1y**,[41] **1a'**,[42] **1b'**,[43] **3a-3j**[44], and **3l-3o**[44] are known compounds and prepared following previously reported procedures. Compounds **1z** and **3k** are new, and their synthetic procedures and characterization data are listed as below.

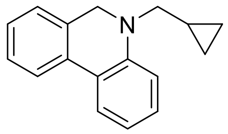

5-(Cyclopropylmethyl)-5,6-dihydrophenanthridine (1z)

The titled compound was synthesized using the general procedure in the literature,[45] and obtained as light yellow oil; ^{1}H NMR ($CDCl_3$, 400 MHz) δ 7.72 (t, J = 9.0 Hz, 2H), 7.33–7.13 (m, 4H), 6.89–6.82 (m, 2H), 4.37 (s, 2H), 3.19 (d, J = 4.0 Hz, 2H), 1.18–1.08 (m, 1H), 0.63–0.58 (m, 2H), 0.30–0.26 (m, 2H); ^{13}C NMR ($CDCl_3$, 101 MHz) δ 146.4, 133.4, 132.2, 129.1, 127.7, 127.0, 125.8, 123.9, 123.4, 122.6, 118.2, 112.8, 55.2, 52.2, 7.5, 4.0 (2C); HRMS (ESI) calcd for $C_{17}H_{18}N$ 236.1434 ($[M+H]^+$), found 236.1425.

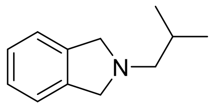

2-Isobutylisoindoline (3k)

The titled compound was synthesized using the general procedure in the literature,[44] and obtained as light yellow oil; ^{1}H NMR ($CDCl_3$, 400 MHz) δ 7.22–7.18 (m, 4H), 3.92 (s, 4H), 2.52 (d, J = 8.0 Hz, 2H), 1.89–1.79 (m, 1H), 1.00 (d, J = 8 Hz, 6H); ^{13}C NMR ($CDCl_3$, 101 MHz) δ 140.4 (2C), 126.7 (2C), 122.4 (2C), 64.9, 59.5 (2C), 27.7, 21.0 (2C); HRMS (ESI) calcd for $C_{12}H_{18}N$ 176.1434 ($[M+H]^+$), found 176.1424.

5. Experimental Procedures for Aerobic Oxygenation Reactions

General Procedure A: Aerobic Oxygenation Reactions under DBU Conditions.

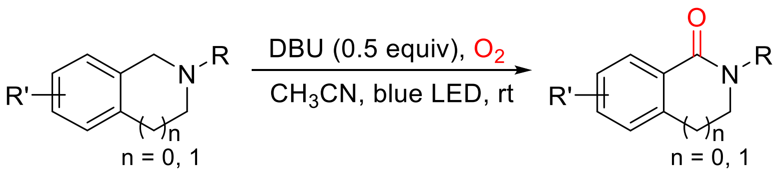

A mixture of substrates (0.15 mmol, 1.0 equiv) and DBU (0.075 mmol, 0.5 equiv) were mixed in a 25 mL Schlenk tube containing a magnetic stirring bar, then adding dry CH_3CN (3 mL). The resulting mixture was stirred at rt with the irradiation of a 72 W blue LED light under

O_2 atmosphere. After completion (TLC monitoring), the reaction solution underwent an aqueous workup and was extracted three times with DCM (10 mL). The combined organic phases were washed with brine, dried over anhydrous Na_2SO_4 and concentrated in vacuo. The crude product was purified by flash column chromatography on silica gel (Petroleum ether/EtOAc) to afford the desired product.

General Procedure B: Aerobic Oxygenation Reactions under DBU/BHT Conditions.

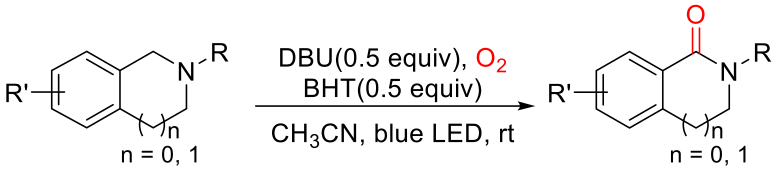

A mixture of substrates (0.15 mmol, 1.0 equiv), DBU (0.075 mmol, 0.5 equiv) and BHT (0.075 mmol, 0.5 equiv) were mixed in a 25 mL Schlenk tube containing a magnetic stirring bar, then adding dry CH_3CN (3 mL). The resulting mixture was stirred at rt with the irradiation of a 72 W blue LED light under O_2 atmosphere. After completion (TLC monitoring), the reaction solution underwent an aqueous workup and was extracted three times with DCM (10 mL). The combined organic phases were washed with brine, dried over anhydrous Na_2SO_4 and concentrated in vacuo. The crude product was purified by flash column chromatography on silica gel (Petroleum ether/EtOAc) to afford the desired product.

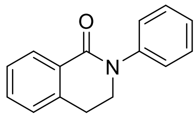

2-Phenyl-3,4-dihydroisoquinolin-1(2H)-one (2a)

Following the general procedure A and B, the crude product was purified by column chromatography on silica gel (Petroleum ether/EtOAc, 10:1) to yield product **2a** (**A**: 26.8 mg, 80%; **B**: 31.8 mg, 95%) as a white solid; m.p. 95-97 °C; ^{1}H NMR ($CDCl_3$, 400 MHz) δ 8.17 (d, J = 9.2 Hz, 1H), 7.44 (td, J = 7.5, 1.5 Hz, 1H), 7.40–7.34 (m, 5H), 7.25–7.20 (m, 2H), 3.93 (t, J = 6.4 Hz, 2H), 3.09 (d, J = 6.4 Hz, 2H); ^{13}C NMR ($CDCl_3$, 101 MHz) δ 164.3, 143.3,

138.5, 132.2, 129.9, 129.0 (2C), 128.8, 127.3, 127.2, 126.3, 125.4 (2C), 49.5, 28.7; HRMS (ESI) calcd for $C_{15}H_{14}NO$ 224.1070 ($[M+H]^+$), found 224.1064.

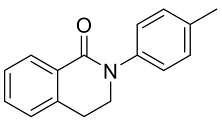

2-(p-Tolyl)-3,4-dihydroisoquinolin-1(2H)-one (2b)

Following the general procedure A and B, the crude product was purified by column chromatography on silica gel (Petroleum ether/EtOAc, 10:1) to yield product **2b** (**A**: 22.4 mg, 63%; **B**: 32.4 mg, 91%) as a white solid; m.p. 112-114 ºC; ^{1}H NMR ($CDCl_3$, 400 MHz) δ 8.15 (d, J = 9.1 Hz, 1H), 7.45 (td, J = 7.5, 1.5 Hz, 1H), 7.36 (td, J = 7.6, 1.3 Hz, 1H), 7.27–7.25 (m, 2H), 7.23–7.19 (m, 3H), 3.95 (t, J = 6.4 Hz, 2H), 3.12 (t, J = 6.4 Hz, 2H), 2.36 (s, 3H); ^{13}C NMR ($CDCl_3$, 101 MHz) δ 164.3, 140.7, 138.4, 136.1, 132.0, 129.9, 129.6 (2C), 128.8, 127.2, 127.0, 125.3 (2C), 49.6, 28.7, 21.1; HRMS (ESI) calcd for $C_{16}H_{16}NO$ 238.1226 ($[M+H]^+$), found 238.1218.

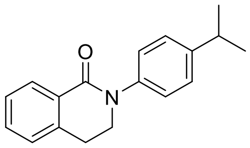

2-(4-Isopropylphenyl)-3,4-dihydroisoquinolin-1(2H)-one (2c)

Following the general procedure A and B, the crude product was purified by column chromatography on silica gel (Petroleum ether/EtOAc, 10:1) to yield product **2c** (**A**: 29.1 mg, 73%; **B**: 32.6 mg, 82%) as a white solid; m.p. 136-139 ºC; ^{1}H NMR ($CDCl_3$, 400 MHz) δ 8.15 (dd, J = 7.7, 1.5 Hz, 1H), 7.45 (td, J = 7.4, 1.5 Hz, 1H), 7.38–7.27 (m, 5H), 7.23 (d, , J = 7.6 Hz, 1H), 3.97 (t, J = 6.5 Hz, 2H), 3.12 (t, J = 6.5 Hz, 2H), 2.95–2.89 (m, 1H), 1.27 (s, 3H), 1.25 (s, 3H); ^{13}C NMR ($CDCl_3$, 101 MHz) δ 164.3, 147.0, 140.9, 138.4, 132.0, 130.0, 128.8, 127.3, 127.0 (3C), 125.2 (2C), 49.6, 33.9, 28.7, 24.1 (2C); HRMS (ESI) calcd for $C_{18}H_{20}NO$ 266.1539 ($[M+H]^+$), found 266.1530.

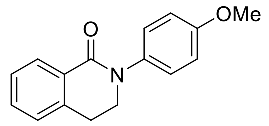

2-(4-Methoxyphenyl)-3,4-dihydroisoquinolin-1(2H)-one (2d)

Following the general procedure A and B, the crude product was purified by column chromatography on silica gel (Petroleum ether/EtOAc, 5:1) to yield product **2d** (**A**: 26.6 mg, 70%; **B**: 36.5 mg, 96%) as a white solid; m.p. 119-121 °C; ^{1}H NMR ($CDCl_3$, 400 MHz) δ 8.14 (d, J = 9.2 Hz, 1H), 7.44 (td, J = 7.4, 1.5 Hz, 1H), 7.36 (t, J = 7.6 Hz, 1H), 7.30–7.26 (m, 2H), 7.22 (d, J = 7.5 Hz, 1H), 6.95–6.91 (m, 2H), 3.93 (t, J = 6.5 Hz, 2H), 3.81 (s, 3H), 3.12 (t, J = 6.5 Hz, 2H); ^{13}C NMR ($CDCl_3$, 101 MHz) δ 164.5, 157.9, 138.4, 136.2, 132.0, 129.9, 128.8, 127.2, 127.0, 126.8 (2C), 114.3 (2C), 55.6, 49.8, 28.7; HRMS (ESI) calcd for $C_{16}H_{16}NO_2$ 254.1176 ([M+H]$^+$), found 254.1167.

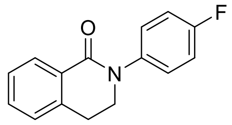

2-(4-Fluorophenyl)-3,4-dihydroisoquinolin-1(2H)-one (2e)

Following the general procedure A and B, the crude product was purified by column chromatography on silica gel (Petroleum ether/EtOAc, 3:1) to yield product **2e** (**A**: 20.6 mg, 57%; **B**: 27.1 mg, 75%) as a white solid; m.p. 117-118 °C; ^{1}H NMR ($CDCl_3$, 400 MHz) δ 8.13 (d, J = 6.2 Hz, 1H), 7.46 (td, J = 7.4, 1.5 Hz, 1H), 7.39–7.31 (m, 3H), 7.23 (d, J = 7.5 Hz, 1H), 7.12–7.05 (m, 2H), 3.95 (t, J = 6.5 Hz, 2H), 3.13 (t, J = 6.5 Hz, 2H); ^{13}C NMR ($CDCl_3$, 101 MHz) δ 164.4, 160.8 (d, $J_{C\text{-}F}$ = 247.4 Hz, 1C), 139.1 (d, $J_{C\text{-}F}$ = 3.0 Hz, 1C), 138.4, 132.2, 129.6, 128.8, 127.2 (d, $J_{C\text{-}F}$ = 10.1 Hz, 2C), 127.1 (d, $J_{C\text{-}F}$ = 5.1 Hz, 2C), 115.8 (d, $J_{C\text{-}F}$ = 23.2 Hz, 2C), 49.7, 28.7; HRMS (ESI) calcd for $C_{15}H_{13}FNO$ 242.0976 ([M+H]$^+$), found 242.0968.

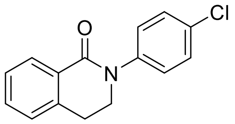

2-(4-Chlorophenyl)-3,4-dihydroisoquinolin-1(2H)-one (2f)

Following the general procedure A and B, the crude product was purified

by column chromatography on silica gel (Petroleum ether/EtOAc, 5:1) to yield product **2f** (**A**: 24.4 mg, 63%; **B**: 30.5 mg, 79%) as a white solid; m.p. 149-150 ºC; ^{1}H NMR ($CDCl_3$, 400 MHz) δ 8.13 (dd, J = 7.8, 1.5 Hz, 1H), 7.46 (td, J = 7.5, 1.5 Hz, 1H), 7.39–7.30 (m, 5H), 7.25–7.22 (m, 1H), 3.95 (t, J = 6.5 Hz, 2H), 3.12 (t, J = 6.4 Hz, 2H); ^{13}C NMR ($CDCl_3$, 101 MHz) δ 164.3, 141.7, 138.4, 132.3, 131.6, 129.5, 129.1 (2C), 128.9, 127.4, 127.1, 126.7 (2C), 49.4, 28.6; HRMS (ESI) calcd for $C_{15}H_{13}ClNO$ 258.0680 ([M+H]$^+$), found 258.0671.

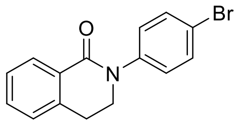

2-(4-Bromophenyl)-3,4-dihydroisoquinolin-1(2H)-one (2g)

Following the general procedure A and B, the crude product was purified by column chromatography on silica gel (Petroleum ether/EtOAc, 10:1) to yield product **2g** (**A**: 27.2 mg, 60%; **B**: 37.2 mg, 82%) as a white solid; m.p. 163-167 ºC; ^{1}H NMR ($CDCl_3$, 400 MHz) δ 8.13 (d, J = 9.2 Hz, 1H), 7.52 (d, J = 2.0 Hz, 2H), 7.46 (td, J = 7.4, 1.5 Hz, 1H), 7.36 (t, J = 6.9 Hz, 1H), 7.29–7.22 (m, 3H), 3.95 (t, J = 6.4 Hz, 2H), 3.13 (t, J = 6.4 Hz, 2H); ^{13}C NMR ($CDCl_3$, 101 MHz) δ 164.2, 142.2, 138.3, 132.3, 132.0 (2C), 129.5, 128.9, 127.4, 127.1, 127.0 (2C), 119.5, 49.3, 28.6; HRMS (ESI) calcd for $C_{15}H_{13}BrNO$ 302.0175 ([M+H]$^+$), found 302.0166.

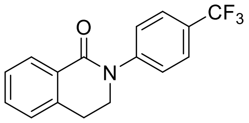

2-(4-(Trifluoromethyl)phenyl)-3,4-dihydroisoquinolin-1(2H)-one (2h) Following the general procedure A and B, the crude product was purified by column chromatography on silica gel (Petroleum ether/EtOAc, 10:1) to yield product **2h** (**A**: 25.3 mg, 58%; **B**: 33.6 mg, 77%) as a white solid; m.p. 169-170 ºC; ^{1}H NMR ($CDCl_3$, 400 MHz) δ 8.15 (d, J = 7.8 Hz, 1H), 7.67 (d, J = 8.5 Hz, 2H), 7.53 (d, J = 8.4 Hz, 2H), 7.49 (td, J = 10.4, 3.7 Hz, 1H), 7.38 (t, J = 7.6 Hz, 1H), 7.25 (d, J = 7.5 Hz, 1H), 4.02 (t, J =

6.4 Hz, 2H), 3.16 (t, J = 6.4 Hz, 2H); ^{13}C NMR ($CDCl_3$, 101 MHz) δ 164.3, 146.2, 138.3, 132.5, 129.4, 129.0, 128.1, 127.8, 127.5, 127.2, 126.1, 126.0, 125.2(2C), 49.2, 28.6; HRMS (ESI) calcd for $C_{16}H_{13}F_3NO$ 292.0944 ($[M+H]^+$), found 292.0934.

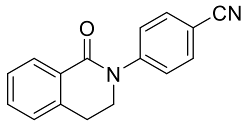

4-(1-oxo-3,4-Dihydroisoquinolin-2(1H)-yl)benzonitrile (2i)

Following the general procedure A and B, the crude product was purified by column chromatography on silica gel (Petroleum ether/EtOAc, 5:1) to yield product **2i** (**A**: 23.8 mg, 64%; **B**: 30.5 mg, 82%) as a white solid; m.p. 118-119 °C; ^{1}H NMR ($CDCl_3$, 400 MHz) δ 8.14 (d, J = 7.7 Hz, 1H), 7.69–7.66 (m, 2H), 7.55–7.47 (m, 3H), 7.38 (t, J = 6.9 Hz, 1H), 7.25 (d, J = 8.7 Hz, 1H), 4.02 (t, J = 6.4 Hz, 2H), 3.16 (t, J = 6.3 Hz, 2H); ^{13}C NMR ($CDCl_3$, 101 MHz) δ 164.2, 147.0, 138.3, 132.8 (2C), 132.8, 129.1, 129.1, 127.6, 127.2, 125.2 (2C), 118.8, 109.1, 49.0, 28.5; HRMS (ESI) calcd for $C_{16}H_{13}N_2O$ 249.1022 ($[M+H]^+$), found 249.1015.

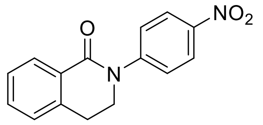

2-(4-Nitrophenyl)-3,4-dihydroisoquinolin-1(2H)-one (2j)

Following the general procedure A and B, the crude product was purified by column chromatography on silica gel (Petroleum ether/EtOAc, 10:1) to yield product **2j** (**A**: 18.1 mg, 45%; **B**: 22.5 mg, 56%) as a yellow solid; m.p. 161-162 °C; ^{1}H NMR ($CDCl_3$, 400 MHz) δ 8.27–8.23 (m, 2H), 8.14 (d, J = 6.4 Hz, 1H), 7.61–7.57 (m, 2H), 7.50 (td, J = 7.4, 1.4 Hz, 1H), 7.39 (t, J = 7.0 Hz, 1H), 7.26 (d, J = 7.6 Hz, 1H), 4.07 (t, J = 6.4 Hz, 2H), 3.18 (t, J = 6.4 Hz, 2H); ^{13}C NMR ($CDCl_3$, 101 MHz) δ 164.3, 148.8, 144.8, 138.3, 132.9, 129.1, 129.0, 127.6, 127.2, 124.9 (2C), 124.3 (2C), 49.1, 28.5; HRMS (ESI) calcd for $C_{15}H_{13}N_2O_3$ 269.0921 ($[M+H]^+$), found 269.0912.

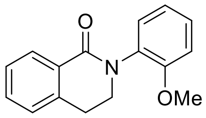

2-(2-Methoxyphenyl)-3,4-dihydroisoquinolin-1(2H)-one (2k)

Following the general procedure A and B, the crude product was purified by column chromatography on silica gel (Petroleum ether/EtOAc, 5:1) to yield product **2k** (**A**: 23.6 mg, 62%; **B**: 30.4 mg, 80%) as colorless oil; ^{1}H NMR ($CDCl_3$, 400 MHz) δ 8.14 (d, J = 9.2 Hz, 1H), 7.44 (td, J = 7.4, 1.5 Hz, 1H), 7.35 (td, J = 7.5, 1.3 Hz, 1H), 7.31–7.27 (m, 2H), 7.22 (d, J = 7.5 Hz, 1H), 7.02–6.98 (m, 2H), 3.86–3.79 (m, 5H), 3.12 (t, J = 6.6 Hz, 2H); ^{13}C NMR ($CDCl_3$, 101 MHz) δ 164.4, 154.8, 138.9, 131.9, 131.7, 129.9, 129.2, 128.8, 128.7, 127.1, 127.0, 121.0, 112.2, 55.8, 49.2, 28.8; HRMS (ESI) calcd for $C_{16}H_{16}NO_2$ 254.1176 ([M+H]$^+$), found 254.1161.

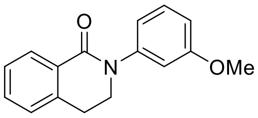

2-(3-Methoxyphenyl)-3,4-dihydroisoquinolin-1(2H)-one (2l)

Following the general procedure A and B, the crude product was purified by column chromatography on silica gel (Petroleum ether/EtOAc, 10:1) to yield product **2l** (**A**: 28.5 mg, 75%; **B**: 35.3 mg, 93%) as a colorless oil; ^{1}H NMR ($CDCl_3$, 400 MHz) δ 8.14 (d, J = 9.2 Hz, 1H), 7.45 (td, J = 7.5, 1.5 Hz, 1H), 7.36 (td, J = 7.5, 1.5 Hz, 1H), 7.32–7.28 (m, 1H), 7.22 (d, J = 7.7 Hz, 1H), 6.96–6.95 (m, 2H), 6.82–6.79 (m, 1H), 3.96 (t, J = 6.4 Hz, 2H), 3.81 (s, 3H), 3.12 (t, J = 6.4 Hz, 2H); ^{13}C NMR ($CDCl_3$, 101 MHz) δ 164.3, 160.1, 144.4, 138.4, 132.1, 129.8, 129.7, 128.8, 127.3, 127.0, 117.6, 112.3, 111.5, 55.5, 49.6, 28.7; HRMS (ESI) calcd for $C_{16}H_{16}NO_2$ 254.1176 ([M+H]$^+$), found 254.1169.

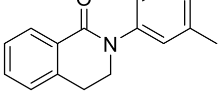

2-(3,5-Dimethylphenyl)-3,4-dihydroisoquinolin-1(2H)-one (2m)

Following the general procedure A and B, the crude product was purified by column chromatography on silica gel (Petroleum ether/EtOAc, 10:1) to yield product **2m** (**A**: 15.1 mg, 40%; **B**: 33.2 mg, 88%) as a white solid; m.p. 93-95 ^{o}C; ^{1}H NMR ($CDCl_3$, 400

MHz) δ 8.15 (d, J = 9.1 Hz, 1H), 7.45 (td, J = 7.4, 1.5 Hz, 1H), 7.36 (t, J = 7.6 Hz, 1H), 7.22 (d, J = 7.6 Hz, 1H), 6.99 (s, 2H), 6.90 (s, 1H), 3.94 (t, J = 6.4 Hz, 2H), 3.12 (t, J = 6.4 Hz, 2H), 2.33 (s, 6H); ^{13}C NMR ($CDCl_3$, 101 MHz) δ 164.3, 143.1, 138.8, 138.4, 132.0, 129.9, 128.8, 128.3, 127.2, 127.0, 123.3 (3C), 49.7, 28.8, 21.4 (2C); HRMS (ESI) calcd for $C_{17}H_{18}NO$ 252.1383 ([M+H]$^+$), found 252.1374.

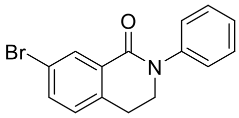

7-Bromo-2-phenyl-3,4-dihydroisoquinolin-1(2H)-one (2n)

Following the general procedure A and B, the crude product was purified by column chromatography on silica gel (Petroleum ether/EtOAc, 10:1) to yield product **2n** (**A**: 30.4 mg, 67%; **B**: 29.5 mg, 65%) as a colorless oil; ^{1}H NMR ($CDCl_3$, 400 MHz) δ 8.27 (d, J = 2.1 Hz, 1H), 7.56 (dd, J = 8.1, 2.2 Hz, 1H), 7.38 (m, 4H), 7.26 (t, J = 7.2 Hz, 1H), 7.12 (d, J = 8.1 Hz, 1H), 3.97 (t, J = 6.5 Hz, 2H), 3.08 (t, J = 6.5 Hz, 2H); ^{13}C NMR ($CDCl_3$, 101 MHz) δ 163.0, 142.9, 137.2, 135.0, 131.7, 131.6, 129.1 (2C), 128.8, 126.6, 125.4 (2C), 121.2, 49.4, 28.2; HRMS (ESI) calcd for $C_{15}H_{13}BrNO$ 302.0175 ([M+H]$^+$), found 302.0165.

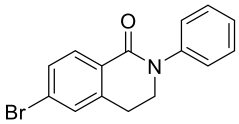

6-Bromo-2-phenyl-3,4-dihydroisoquinolin-1(2H)-one (2o)

Following the general procedure A and B, the crude product was purified by column chromatography on silica gel (Petroleum ether/EtOAc, 10:1) to yield product **2o** (**A**: 28.2 mg, 62%; **B**: 29.1 mg, 64%) as a white solid; m.p. 172-174 ºC; ^{1}H NMR ($CDCl_3$, 400 MHz) δ 8.00 (d, J = 8.3 Hz, 1H), 7.50 (dd, J = 8.3, 2.0 Hz, 1H), 7.42–7.38 (m, 3H), 7.37–7.34 (m, 2H), 7.27–7.23 (m, 1H), 3.97 (t, J = 6.6 Hz, 2H), 3.11 (t, J = 6.4 Hz, 2H); ^{13}C NMR ($CDCl_3$, 101 MHz) δ 163.6, 142.9, 140.2, 130.6, 130.0, 129.5, 129.1 (2C), 128.8, 126.8, 126.6, 125.4 (2C), 49.3, 28.5; HRMS (ESI) calcd for $C_{15}H_{13}BrNO$ 302.0175 ([M+H]$^+$), found 302.0165.

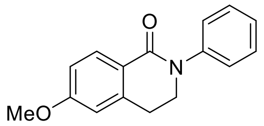

6-Methoxy-2-phenyl-3,4-dihydroisoquinolin-1(2H)-one (2p)

Following the general procedure A and B, the crude product was purified by column chromatography on silica gel (Petroleum ether/EtOAc, 5:1) to yield product **2p** (**A**: 29.3 mg, 77%; **B**: 33.1 mg, 87%) as a white solid; m.p. 140-142 °C; ^{1}H NMR ($CDCl_3$, 400 MHz) *δ* 8.09 (d, *J* = 8.6 Hz, 1H), 7.41–7.35 (m, 4H), 7.25–7.20 (m, 1H), 6.87 (dd, *J* = 8.7, 2.6 Hz, 1H), 6.71 (d, *J* = 2.5 Hz, 1H), 3.96 (t, *J* = 6.5 Hz, 2H), 3.85 (s, 3H), 3.09 (t, *J* = 6.4 Hz, 2H); ^{13}C NMR ($CDCl_3$, 101 MHz) δ 164.3, 162.6, 143.4, 140.5, 131.0, 128.9 (2C), 126.1, 125.4 (2C), 122.7, 112.8, 112.0, 55.5, 49.4, 29.1; HRMS (ESI) calcd for $C_{16}H_{16}NO_2$ 254.1176 ([M+H]$^+$), found 254.1166.

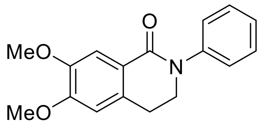

6,7-Dimethoxy-2-phenyl-3,4-dihydroisoquinolin-1(2H)-one (2q)

Following the general procedure A and B, the crude product was purified by column chromatography on silica gel (Petroleum ether/EtOAc, 3:1) to yield product **2q** (**A**: 31.4 mg, 74%; **B**: 37.4 mg, 88%) as a white solid; m.p. 163-165 °C; ^{1}H NMR ($CDCl_3$, 400 MHz) *δ* 7.65 (s, 1H), 7.37 (m, 4H), 7.22 (m, 1H), 6.67 (s, 1H), 3.96 (t, *J* = 6.5 Hz, 2H), 3,92 (s, 3H), 3.91 (s, 3H), 3.05 (t, *J* = 6.2 Hz, 2H); ^{13}C NMR ($CDCl_3$, 101 MHz) δ 164.3, 152.3, 148.2, 143.4, 132.2, 128.9 (2C), 126.1, 125.4 (2C), 122.3, 111.0, 109.3, 56.2 (2C), 49.7, 28.3; HRMS (ESI) calcd for $C_{17}H_{18}NO_3$ 284.1281 ([M+H]$^+$), found 284.1272.

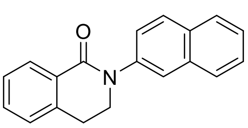

2-(Naphthalen-2-yl)-3,4-dihydroisoquinolin-1(2H)-one (2r)

Following the general procedure A and B, the crude product was purified by column chromatography on silica gel (Petroleum ether/EtOAc, 10:1) to yield product **2r** (**A**: 30.3 mg, 74%; **B**: 34.8 mg, 85%) as a white solid; m.p. 169-170 °C; ^{1}H NMR

(CDCl$_3$, 400 MHz) δ 8.20 (dd, J = 7.7, 1.5 Hz, 1H), 7.82 (m, 4H), 7.58 (dd, J = 8.8, 2.1 Hz, 1H), 7.47 (m, 3H), 7.39 (t, J = 7.0 Hz, 1H), 7.26 (d, J = 8.0 Hz, 1H), 4.09 (t, J = 6.5 Hz, 2H), 3.18 (t, J = 6.4 Hz, 2H); ^{13}C NMR (CDCl$_3$, 101 MHz) δ164.5, 141.0, 138.5, 133.7, 132.2, 131.8, 129.8, 128.9, 128.6, 127.9, 127.8, 127.3, 127.1, 126.4, 126.0, 124.7, 122.7, 49.8, 28.8; HRMS (ESI) calcd for $C_{19}H_{16}NO$ 274.1226 ([M+H]$^+$), found 274.1217.

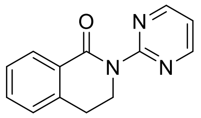

2-(Pyrimidin-2-yl)-3,4-dihydroisoquinolin-1(2H)-one (2s)

Following the general procedure A and B, the crude product was purified by column chromatography on silica gel (Petroleum ether/EtOAc, 1:1) to yield product **2s** (**A**: 17.6 mg, 52%; **B**: 22.6 mg, 67%) as a colorless oil; ^{1}H NMR (CDCl$_3$, 400 MHz) δ 8.75 (s, 2H), 8.24 (d, J = 6.4 Hz, 1H), 7.46 (td, J = 7.4, 1.4 Hz, 1H), 7.36 (t, J = 7.0 Hz, 1H), 7.23 (d, J = 7.4 Hz, 1H), 7.10 (t, J = 4.7 Hz, 1H), 4.25 (t, J = 6.3 Hz, 2H), 3.13 (t, J = 6.3 Hz, 2H); ^{13}C NMR (CDCl$_3$, 101 MHz) δ 164.6, 161.0, 158.0, 139.2, 132.7 (2C), 129.5 (2C), 127.3 (2C), 117.6, , 46.9, 28.6; HRMS (ESI) calcd for $C_{13}H_{12}N_3O$ 226.0975 ([M+H]$^+$), found 226.0968.

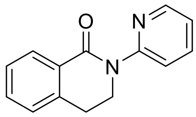

2-(Pyridin-2-yl)-3,4-dihydroisoquinolin-1(2H)-one (2t)

Following the general procedure A and B, the crude product was purified by column chromatography on silica gel (Petroleum ether/EtOAc, 10:1) to yield product **2t** (**A**: 20.9 mg, 62%; **B**: 23.9 mg, 71%) as a colorless oil; ^{1}H NMR (CDCl$_3$, 400 MHz) δ 8.43 (m, 1H), 8.17(dd, J = 7.8, 1.4 Hz, 1H), 8.02(dt, J = 8.4, 1.0 Hz, 1H), 7.71 (m, 1H), 7.47 (td, J = 7.4, 1.4 Hz, 1H), 7.37 (t, J = 7.3 Hz, 1H), 7.25 (d, J = 7.52 Hz, 1H), 7.08(m, 1H), 4.30 (t, J = 6.4 Hz, 2H), 3.11 (t, J = 6.4 Hz, 2H); ^{13}C NMR (CDCl$_3$, 101 MHz) δ164.9, 154.2, 147.7, 139.3, 137.0, 132.5, 129.9, 129.0, 127.2, 127.1, 120.3 (2C), 45.8, 28.6; HRMS (ESI) calcd for $C_{14}H_{13}N_2O$ 225.1022 ([M+H]$^+$), found 225.1016.

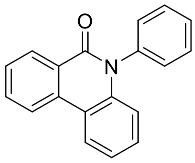

5-Phenylphenanthridin-6(5H)-one (2u)

Following the general procedure A and B, the crude product was purified by column chromatography on silica gel (Petroleum ether/EtOAc, 10:1) to yield product **2u** (**A**: 16.8 mg, 41%; **B**: 20.5 mg, 50%) as a white solid; m.p. 227-229 ºC; ^{1}H NMR ($CDCl_3$, 400 MHz) *δ* 8.00 (dd, *J* = 7.9, 1.4 Hz, 1H), 8.35–8.28 (m, 2H), 7.42–7.81 (ddd, *J* = 8.3, 7.1, 1.5 Hz, 1H), 7.63–7.59 (m, 3H), 7.55–7.51 (m, 1H), 7.35–7.27 (m, 4H), 6.71–6.67 (m, 1H); ^{13}C NMR ($CDCl_3$, 101 MHz) δ 161.8, 139.3, 138.4, 134.1, 132.9, 130.3 (2C), 129.2 (3C), 129.1, 128.9, 128.2, 126.0, 123.1, 122.7, 121.9, 119.1, 117.1; HRMS (ESI) calcd for $C_{19}H_{14}NO$ 272.1070 ([M+H]$^+$), found 272.1061.

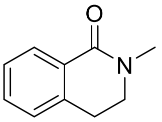

2-Methyl-3,4-dihydroisoquinolin-1(2H)-one (2v)

Following the general procedure A and B, the crude product was purified by column chromatography on silica gel (Petroleum ether/EtOAc, 3:1) to yield product **2v** (**A**: 14.5 mg, 60%; **B**:19.0 mg, 79%) as a colorless oil; ^{1}H NMR ($CDCl_3$, 400 MHz) *δ* 8.06 (dd, *J* = 7.7, 1.5 Hz, 1H), 7.38 (td, *J* = 7.5, 1.5 Hz, 1H), 7.32–7.28 (m, 1H), 7.14 (d, *J* = 7.4 Hz, 1H), 3.54 (t, *J* = 6.7 Hz, 2H), 3.13 (s, 3H), 2.98 (t, *J* = 6.7 Hz, 2H); ^{13}C NMR ($CDCl_3$, 101 MHz) *δ* 164.9, 138.0, 131.6, 129.5, 128.2, 127.1, 126.9, 48.2, 35.2, 28.0; HRMS (ESI) calcd for $C_{10}H_{12}NO$ 162.0913 ([M+H]$^+$), found 162.0911.

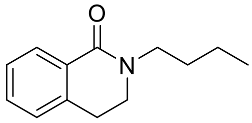

2-Butyl-3,4-dihydroisoquinolin-1(2H)-one (2w)

Following the general procedure A and B, the crude product was purified by column chromatography on silica gel (Petroleum ether/EtOAc, 10:1) to yield product **2w** (**A**: 16.0 mg, 53%; **B**: 20.2 mg, 66%) as a colorless oil; ^{1}H NMR ($CDCl_3$, 400 MHz) *δ* 8.06 (dd, *J* = 7.7, 1.5 Hz, 1H), 7.38 (td, *J* = 7.4, 1.6 Hz, 1H), 7.33–7.29 (m, 1H), 7.15 (d, *J* =

7.4 Hz, 1H), 3.57–3.52 (m, 4H), 2.96 (t, *J* = 6.6 Hz, 2H), 1.64–1.56 (m, 2H), 1.42–1.33 (m, 2H), 0.94 (t, *J* = 7.4 Hz, 3H); ^{13}C NMR ($CDCl_3$, 101 MHz) *δ* 164.3, 138.0, 131.5, 129.8, 128.3, 127.1, 126.8, 47.3, 46.1, 29.9, 28.3, 20.3, 14.0; HRMS (ESI) calcd for $C_{13}H_{18}NO$ 204.1383 ([M+H]$^+$), found 204.1376.

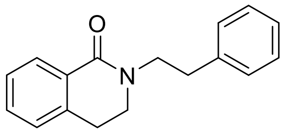

2-Phenethyl-3,4-dihydroisoquinolin-1(2H)-one (2x)

Following the general procedure A and B, the crude product was purified by column chromatography on silica gel (Petroleum ether/EtOAc, 10:1) to yield product **2x** (**A**: 13.9 mg, 37%; **B**: 26.1 mg, 69%) as a colorless oil; ^{1}H NMR ($CDCl_3$, 400 MHz) *δ* 8.08 (dd, *J* = 7.6, 1.5 Hz, 1H), 7.39 (td, *J* = 7.4, 1.6 Hz, 1H), 7.34–7.29 (m, 2H), 7.27–7.20 (m, 4H), 7.12 (tt, *J* = 7.5 Hz, 1H), 3.78 (t, *J* = 7.2 Hz, 2H), 3.37 (t, *J* = 6.6 Hz, 2H), 2.96 (t, *J* = 7.2 Hz, 2H), 2.83 (t, *J* = 6.6 Hz, 2H); ^{13}C NMR ($CDCl_3$, 101 MHz) *δ* 164.5, 139.4, 138.2, 131.6, 129.6, 129.0 (2C), 128.6 (2C), 128.2, 127.1, 126.9, 126.5, 50.1, 47.2, 34.4, 28.1; HRMS (ESI) calcd for $C_{17}H_{17}NONa$ 274.1202 ([M+Na]$^+$), found 274.1199.

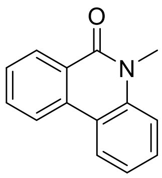

5-Methylphenanthridin-6(5H)-one (2y)

Following the general procedure A and B, the crude product was purified by column chromatography on silica gel (Petroleum ether/EtOAc, 30:1) to yield product **2y** (**A**: 17.8 mg, 56%; **B**: 27.3 mg, 87%) as a white solid; m.p. 110-111 ^{o}C; ^{1}H NMR ($CDCl_3$, 400 MHz) *δ* 8.51 (dd, *J* = 7.9, 1.4 Hz, 1H), 8.20 (d, *J* = 7.8 Hz, 2H), 7.72–7.68 (m, 1H), 7.56–7.47 (m, 2H), 7.35 (d, *J* = 8.6 Hz, 1H), 7.29–7.25 (m, 1H), 3.76 (s, 3H); ^{13}C NMR ($CDCl_3$, 101 MHz) *δ* 161.7, 138.1, 133.6, 132.4, 129.6, 128.9, 128.0, 125.6, 123.3, 122.5, 121.7, 119.3, 115.1, 30.0; HRMS (ESI) calcd for $C_{14}H_{12}NO$ 210.0913 ([M+H]$^+$), found 210.0908.

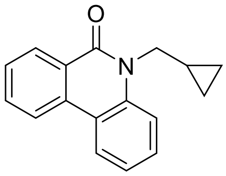

5-(Cyclopropylmethyl)phenanthridin-6(5H)-one (2z)

Following the general procedure A and B, the crude product was purified by column chromatography on silica gel (Petroleum ether/EtOAc, 30:1) to yield product **2z** (**A**: 22.8 mg, 61%; **B**: 34.0 mg, 90%) as a white solid; m.p. 107-108 °C; ^{1}H NMR ($CDCl_3$, 400 MHz) δ 8.53 (dd, J = 8.0, 1.5 Hz, 1H), 8.27 (t, J = 8.8 Hz, 2H), 7.76–7.71 (m, 1H), 7.59–7.51 (m, 3H), 7.30 (ddd, J = 8.1, 6.1, 2.1 Hz, 1H), 4.35 (d, J = 6.8 Hz, 2H), 1.35–1.27 (m, 1H), 0.63–0.58 (m, 2H), 0.55–0.50 (m, 2H); ^{13}C NMR ($CDCl_3$, 101 MHz) δ 161.8, 137.5, 133.7, 132.5, 129.6, 129.1, 128.0, 125.8, 123.5, 122.4, 121.7, 119.5, 115.5, 46.3, 9.9, 4.2 (2C); HRMS (ESI) calcd for $C_{17}H_{15}NONa$ 272.1046 ([M+Na]$^+$), found 272.1042.

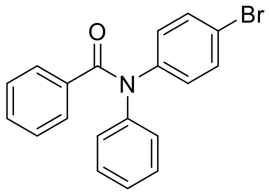

***N*-(4-Bromophenyl)-N-phenylbenzamide (2a')**

Following the general procedure A and B, the crude product was purified by column chromatography on silica gel (Petroleum ether/EtOAc, 30:1) to yield product **2a'** (**A**: 18.1 mg, 34%; **B**: 24.6 mg, 46%) as a colorless oil; ^{1}H NMR ($CDCl_3$, 400 MHz) δ 7.46–7.39 (m, 4H), 7.32–7.25 (m, 3H), 7.23–7.16 (m, 3H), 7.12–7.08 (m, 2H), 7.06–7.02 (m, 2H); ^{13}C NMR ($CDCl_3$, 101 MHz) δ 170.6, 143.6, 143.1, 135.8, 132.3 (2C), 130.5, 129.4 (2C), 129.3 (2C), 128.9 (2C), 128.1 (2C), 127.7 (2C), 126.8, 119.9; HRMS (ESI) calcd for $C_{19}H_{14}BrNONa$ 374.0151 ([M+Na]$^+$), found 374.0150.

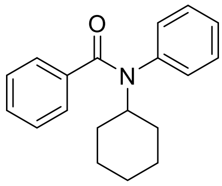

***N*-Cyclohexyl-N-phenylbenzamide (2b')**

Following the general procedure A and B, the crude product was purified by column chromatography on silica gel (Petroleum ether/EtOAc, 30:1) to yield product **2b'** (**A**: 11.3 mg, 27%; **B**: 20.8 mg, 50%) as a colorless oil; ^{1}H NMR

($CDCl_3$, 400 MHz) *δ* 7.25–7.14 (m, 5H), 7.13–7.06 (m, 3H), 7.00–6.98 (m, 2H), 4.72–4.66 (m, 1H), 1.97–1.93 (m, 2H), 1.78–1.74 (m, 2H), 1.61–1.57 (m, 1H), 1.45–1.38 (m, 2H), 1.25–1.16 (m, 2H), 1.01–0.89 (m, 1H); ^{13}C NMR ($CDCl_3$, 101 MHz) *δ* 170.7, 140.0, 137.4, 130.9 (2C), 128.9, 128.6 (2C), 128.1 (2C), 127.6 (2C), 127.4, 55.6, 31.8 (2C), 26.0 (2C), 25.5; HRMS (ESI) calcd for $C_{19}H_{22}NO$ 280.1695 ($[M+H]^+$), found 280.1686.

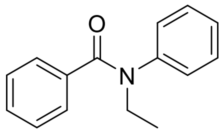

N-Ethyl-N-phenylbenzamide (2c')

Following the general procedure A and B, the crude product was purified by column chromatography on silica gel (Petroleum ether/EtOAc, 30:1) to yield product **2b'** (**A**: 11.5 mg, 34%; **B**: 12.8 mg, 38%) as a colorless oil; ^{1}H NMR ($CDCl_3$, 400 MHz) *δ* 7.24–7.21 (m, 2H), 7.13–7.07 (m, 3H), 7.05–7.00 (m, 3H), 6.95–6.93 (m, 2H), 3.90 (q, *J* = 7.1 Hz, 2H), 1.13 (t, *J* = 7.1 Hz, 3H); ^{13}C NMR ($CDCl_3$, 101 MHz) *δ* 170.1, 143.3, 136.4, 129.5, 129.2 (2C), 128.7 (2C), 128.0 (2C), 127.7(2C), 126.7, 45.4, 13.0; HRMS (ESI) calcd for $C_{15}H_{16}NO$ 226.1226 ($[M+H]^+$), found 226.1217.

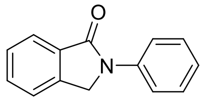

2-Phenylisoindolin-1-one (4a)

Following the general procedure A and B, the crude product was purified by column chromatography on silica gel (Petroleum ether/EtOAc, 10:1) to yield product **4a** (**A**: 24.2 mg, 77%; **B**: 24.5 mg, 78%) as a white solid; m.p. 157-159 ºC; ^{1}H NMR ($CDCl_3$, 400 MHz) *δ* 7.91 (dd, *J* = 7.2, 1.4 Hz, 1H), 7.87–7.84 (m, 2H), 7.60–7.56 (m, 1H), 7.51–7.47 (m, 2H), 7.44–7.39 (m, 2H), 7.18–7.14 (m, 1H), 4.83 (s, 2H); ^{13}C NMR ($CDCl_3$, 101 MHz) δ 167.6, 140.2, 139.6, 133.3, 132.2, 129.3 (2C), 128.5, 124.5, 124.2, 122.7, 119.5 (2C), 50.8; HRMS (ESI) calcd for $C_{14}H_{12}NO$ 210.0913 ($[M+H]^+$), found 210.0908.

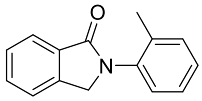

2-(o-Tolyl)isoindolin-1-one (4b)

Following the general procedure A and B (DMF as solvent), the crude product was purified by column chromatography on silica gel (Petroleum ether/EtOAc, 5:1) to yield product **4b** (**A**: 17.1 mg, 51%; **B**: 20.8 mg, 62%) as a colorless oil; ^{1}H NMR ($CDCl_3$, 400 MHz) δ 7.95 (d, J = 7.4 Hz, 1H), 7.60 (td, J = 7.4, 1.2 Hz, 1H), 7.54–7.49 (m, 2H), 7.34–7.23 (m, 4H), 4.72 (s, 2H), 2.26 (s, 3H); ^{13}C NMR ($CDCl_3$, 101 MHz) δ 167.7, 141.7, 137.1, 136.5, 132.6, 131.7, 131.3, 128.4, 128.3, 127.6, 127.0, 124.4, 122.9, 53.1, 18.3; HRMS (ESI) calcd for $C_{15}H_{14}NO$ 224.1070 ($[M+H]^+$), found 224.1066.

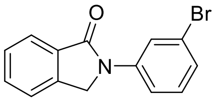

2-(3-Bromophenyl)isoindolin-1-one (4c)

Following the general procedure A and B, the crude product was purified by column chromatography on silica gel (Petroleum ether/EtOAc, 10:1) to yield product **4c** (**A**: 31.1 mg, 72%; **B**: 32.4 mg, 75%) as a white solid; m.p. 178-180 °C; ^{1}H NMR ($CDCl_3$, 400 MHz) δ 8.04–8.03 (m, 1H), 7.90–7.88 (m, 1H), 7.86–7.83 (m, 1H), 7.59 (td, J = 7.4, 1.3 Hz, 1H), 7.51–7.47 (m, 2H), 7.28–7.23 (m, 2H), 4.80 (s, 2H); ^{13}C NMR ($CDCl_3$, 101 MHz) δ 167.6, 140.9, 140.0, 132.9, 132.5, 130.5, 128.6, 127.3, 124.3, 123.0, 122.8, 122.1, 117.6, 50.7; HRMS (ESI) calcd for $C_{14}H_{11}BrNO$ 288.0019 ($[M+H]^+$), found 288.0010.

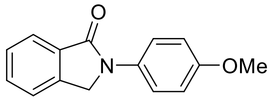

2-(4-Methoxyphenyl)isoindolin-1-one (4d)

Following the general procedure A and B (DMF as solvent), the crude product was purified by column chromatography on silica gel (Petroleum ether/EtOAc, 5:1) to yield product **4d** (**A**: 21.9 mg , 61%; **B**: 24.4 mg, 68%) as a white solid; m.p. 144-145 °C; ^{1}H NMR ($CDCl_3$, 400 MHz) δ 7.90 (d, J = 8.1 Hz, 1H), 7.74–7.70 (m, 2H), 7.56 (td, J = 7.4, 1.2

Hz, 1H), 7.51–7.45 (m, 2H), 6.97–6.93 (m, 2H), 4.79 (s, 2H), 3.81 (s, 3H); ^{13}C NMR ($CDCl_3$, 101 MHz) δ 167.3, 156.7, 140.2, 133.4, 132.8, 131.9, 128.4, 124.1, 122.6, 121.6 (2C), 114.4 (2C), 55.6, 51.3; HRMS (ESI) calcd for $C_{15}H_{14}NO_2$ 240.1025 ([M+H]$^+$), found 240.1031.

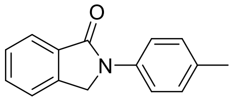

2-(p-Tolyl)isoindolin-1-one (4e)

Following the general procedure A and B, the crude product was purified by column chromatography on silica gel (Petroleum ether/EtOAc, 10:1) to yield product **4e** (**A**: 22.8 mg, 68%; **B**: 26.5 mg, 79%) as a white solid; m.p. 126-128 ºC; ^{1}H NMR ($CDCl_3$, 400 MHz) *δ* 7.90 (d, *J* = 8.2 Hz, 1H), 7.73–7.70 (m, 2H), 7.56 (td, *J* = 7.4, 1.2 Hz, 1H), 7.50–7.46 (m, 2H), 7.22–7.19 (m, 2H), 4.79 (s, 2H), 2.33 (s, 3H); ^{13}C NMR ($CDCl_3$, 101 MHz) δ 167.5, 140.2, 137.1, 134.3, 133.4, 132.0, 129.8 (2C), 128.4, 124.1, 122.7, 119.7 (2C), 50.9, 20.9; HRMS (ESI) calcd for $C_{15}H_{14}NO$ 224.1070 ([M+H]$^+$), found 224.1063.

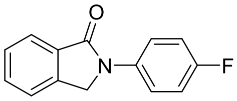

2-(4-Fluorophenyl)isoindolin-1-one (4f)

Following the general procedure A and B, the crude product was purified by column chromatography on silica gel (Petroleum ether/EtOAc, 10:1) to yield product **4f** (**A**: 24.9 mg, 73%; **B**: 25.2 mg, 74%) as a white solid; m.p. 170-171 ºC; ^{1}H NMR ($CDCl_3$, 400 MHz) *δ* 7.89 (d, *J* = 8.3 Hz, 1H), 7.81–7.78 (m, 2H), 7.57 (td, *J* = 7.4, 1.2 Hz, 1H), 7.50–7.46 (m, 2H), 7.11–7.06 (m, 2H), 4.79 (s, 2H); ^{13}C NMR ($CDCl_3$, 101 MHz) *δ* 167.5, 159.6 (d, J_{C-F} = 245.4 Hz, 1C), 140.1, 135.7 (d, J_{C-F} = 3.0 Hz, 1C), 133.1, 132.2, 128.5, 124.2, 122.7, 121.3 (d, J_{C-F} = 7.1 Hz, 2C), 115.9 (d, J_{C-F} = 22.2 Hz, 2C), 51.0; HRMS (ESI) calcd for $C_{14}H_{11}FNO$ 228.0819 ([M+H]$^+$), found 228.0812.

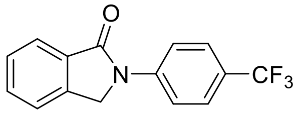

2-(4-(Trifluoromethyl)phenyl)isoindolin-1-one (4g)

Following the general procedure A and B, the crude product was purified by column chromatography on silica gel (Petroleum ether/EtOAc, 10:1) to yield product **4g** (**A**: 26.6 mg, 64%; **B**: 29.5 mg, 71%) as a white solid; m.p. 230-232 ºC; ^{1}H NMR ($CDCl_3$, 400 MHz) δ 8.02 (d, J = 8.3 Hz, 2H), 7.92 (d, J = 7.4 Hz, 1H), 7.68–7.60 (m, 3H), 7.54–7.50 (m, 2H), 4.88 (s, 2H); ^{13}C NMR ($CDCl_3$, 101 MHz) δ 167.9, 142.6, 140.0, 132.8, 132.8, 128.8, 126.5, 126.5, 126.4, 126.4, 124.5, 122.8, 118.7 (2C), 50.6; HRMS (ESI) calcd for $C_{15}H_{11}F_3NO$ 278.0787 ([M+H]$^+$), found 278.0779.

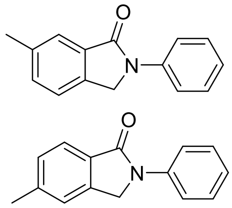

6-Methyl-2-phenylisoindolin-1-one/5-Methyl-2-phenylisoindolin-1-one (4h) Following the general procedure A and B, the crude product was purified by column chromatography on silica gel (Petroleum ether/EtOAc, 10:1) to yield product **4h** (**A**: 14.4 mg, 43%; **B**: 18.8 mg, 56%) as a white solid; **6-Methyl-2-phenylisoindolin-1-one (1:0.6)**; ^{1}H NMR ($CDCl_3$, 400 MHz) δ 7.86 (m, 2H), 7.71 (s, 1H), 7.43–7.38 (m, 4H), 7.18–7.13 (m, 1H), 4.80 (s, 2H), 2.47 (s, 3H); ^{13}C NMR ($CDCl_3$, 101 MHz) δ 167.7, 139.8, 138.5, 137.4, 133.5, 133.2, 129.2 (2C), 124.4, 124.4, 122.4, 119.5 (2C), 50.6, 21.5; **5-Methyl-2-phenylisoindolin-1-one (1:**0.6**)**; ^{1}H NMR ($CDCl_3$, 400 MHz) δ 7.84 (m, 2H), 7.79 (m, 1H), 7.43–7.38 (m, 4H), 7.30–7.29 (m, 1H), 4.79 (s, 2H), 2.45 (s, 3H); ^{13}C NMR ($CDCl_3$, 101 MHz) δ 167.7, 142.9, 140.6, 139.7, 130.8, 129.5, 129.2 (2C), 124.4, 124.0, 123.1, 119.4 (2C), 50.6, 22.1,; HRMS (ESI) calcd for $C_{15}H_{14}NO$ 224.1070 ([M+H]$^+$), found 224.1061.

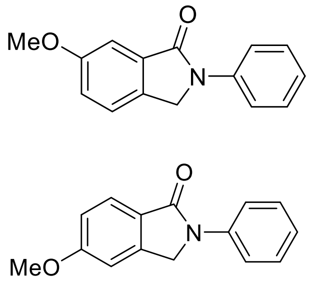

6-Methoxy-2-phenylisoindolin-1-one/5-Methoxy-2-phenylisoindolin-1-one (4i) Following the general procedure A and B, the crude product was purified by column chromatography on silica gel (Petroleum ether/EtOAc, 10:1) to yield product **6-Methoxy-2-phenylisoindolin-1-one** (**A**: 6.1 mg, 17%; **B**: 8.0 mg, 22%) as a white solid; m.p. 180-182 °C; ^{1}H NMR ($CDCl_3$, 400 MHz) δ 7.84 (d, J = 7.7 Hz, 2H), 7.43–7.37 (m, 4H), 7.18–7.12 (m, 2H), 4.77 (s, 2H), 3.87 (s, 3H); ^{13}C NMR ($CDCl_3$, 101 MHz) δ 167.6, 160.3, 139.7, 134.6, 132.4, 129.2 (2C), 124.6, 123.6, 120.8, 119.5 (2C), 106.7, 56.3, 50.4; HRMS (ESI) calcd for $C_{15}H_{14}NO_2$ 240.1019 ([M+H]$^+$), found 240.1010. **5-Methoxy-2-phenylisoindolin-1-one** (**A**: 12.5 mg, 35%; **B**: 14.1 mg, 39%) as a white solid; m.p. 140-142 °C; ^{1}H NMR ($CDCl_3$, 400 MHz) δ 7.85–7.81 (m, 3H), 7.43–7.39 (m, 2H), 7.17–7.13 (m, 1H), 7.01 (dd, J = 8.4, 2.2 Hz, 1H), 6.98 (m, 1H), 4.79 (s, 2H), 3.89 (s, 3H); ^{13}C NMR ($CDCl_3$, 101 MHz) δ 167.5, 163.4, 142.5, 139.8, 129.2 (2C), 126.0, 125.7, 124.2, 119.3 (2C), 115.2, 107.4, 55.8, 50.6; HRMS (ESI) calcd for $C_{15}H_{14}NO_2$ 240.1019 ([M+H]$^+$), found 240.1010.

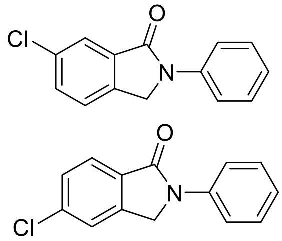

6-Chloro-2-phenylisoindolin-1-one/5-Chloro-2-phenylisoindolin-1-one (4j) Following the general procedure A and B, the crude product was purified by column chromatography on silica gel (Petroleum ether/EtOAc, 10:1) to yield product **4j** (**A**: 21.6 mg, 59%; **B**: 24.1 mg, 66%) as a white solid; **6-Chloro-2-phenylisoindolin-1-one (1**:1**)**; ^{1}H NMR ($CDCl_3$, 400 MHz) δ 7.88 (d, J = 2.0 Hz, 1H), 7.83–7.81 (m, 2H), 7.54 (dd, J = 8.0, 2.0 Hz, 1H), 7.46–7.39 (m, 3H), 7.21–7.16 (m, 1H), 4.82 (s, 2H); ^{13}C NMR ($CDCl_3$, 101 MHz) δ 166.5, 141.7, 139.2, 138.3, 135.1, 134.8, 132.4, 129.2 (2C), 124.8, 124.4, 119.6 (2C), 50.4; **5-Chloro-2-**

phenylisoindolin-1-one (1:**1)**; ^{1}H NMR ($CDCl_3$, 400 MHz) δ 7.84 (d, J = 2.9 Hz, 1H), 7.83–7.81 (m, 2H), 7.50–7.48 (m, 1H), 7.46–7.39 (m, 3H), 7.21–7.16 (m, 1H), 4.82 (s, 2H); ^{13}C NMR ($CDCl_3$, 101 MHz) δ 166.2, 141.7, 139.3, 138.5, 131.8, 129.3 (2C), 125.5, 124.9, 124.0, 123.2, 119.6 (2C), 50.5; HRMS (ESI) calcd for $C_{14}H_{11}ClNO$ 244.0523 ([M+H]$^{+}$), found 244.0516.

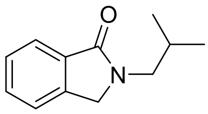

2-Isobutylisoindolin-1-one (4k)

Following the general procedure A and B, the crude product was purified by column chromatography on silica gel (Petroleum ether/EtOAc, 5:1) to yield product **4k** (**A**: 9.6 mg, 34%; **B**: 13.0 mg, 46%) as a colorless oil; ^{1}H NMR ($CDCl_3$, 400 MHz) δ 7.84 (d, J = 7.4 Hz, 1H), 7.51 (td, J = 7.4, 1.3 Hz, 1H), 7.45–7.41 (m, 2H), 4.36 (s, 2H), 3.41 (d, J = 7.5 Hz, 2H), 2.10–1.99 (m, 1H), 0.95 (s, 3H), 0.93 (s, 3H); ^{13}C NMR ($CDCl_3$, 101 MHz) δ 168.9, 141.2, 133.0, 131.2, 128.1, 123.9, 122.7, 50.7, 50.2, 27.9, 20.2 (2C); HRMS (ESI) calcd for $C_{12}H_{15}NONa$ 212.1046 ([M+Na]$^{+}$), found 212.1046.

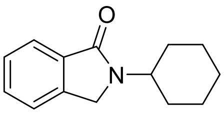

2-Cyclohexylisoindolin-1-one (4l)

Following the general procedure A and B, the crude product was purified by column chromatography on silica gel (Petroleum ether/EtOAc, 10:1) to yield product **4l** (**A**: 11.7 mg, 37%; **B**: 16.0 mg, 50%) as a colorless oil; ^{1}H NMR ($CDCl_3$, 400 MHz) δ 7.86–7.84 (m, 1H), 7.52–7.48 (m, 1H), 7.45–7.42 (m, 2H), 4.34 (s, 2H), 4.27–4.21 (m, 1H), 1.88–1.83 (m, 4H), 1.74–1.69 (m, 1H), 1.52–1.43 (m, 4H), 1.19–1.13 (m, 1H); ^{13}C NMR ($CDCl_3$, 101MHz) δ 168.0, 141.4, 133.4, 131.1, 128.0, 123.8, 122.7, 50.7, 46.1, 31.5 (2C), 25.7 (2C), 25.6; HRMS (ESI) calcd for $C_{14}H_{17}NONa$ 238.1202 ([M+Na]$^{+}$), found 238.1201.

2-Cyclopropylisoindolin-1-one (4m)

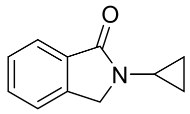

Following the general procedure A and B, the crude product was purified by column chromatography on silica gel (Petroleum ether/EtOAc, 10:1) to yield product **4m** (**A**: 7.3 mg, 28%; **B**: 8.5 mg, 32%) as a colorless oil; ^{1}H NMR ($CDCl_3$, 400 MHz) δ 7.80 (d, J = 7.4 Hz, 1H), 7.49 (td, J = 7.4, 1.2 Hz, 1H), 7.43–7.37 (m, 2H), 4.29 (s, 2H), 2.94–2.89 (m, 1H), 0.91–0.88 (m, 2H), 0.86–0.82 (m, 2H); ^{13}C NMR ($CDCl_3$, 101 MHz) δ 169.8, 141.2, 133.3, 131.4, 128.1, 123.7, 122.7, 50.5, 25.3, 5.7 (2C); HRMS (ESI) calcd for $C_{11}H_{12}NO$ 174.0913 ($[M+H]^+$), found 174.0909.

2-Benzylisoindolin-1-one (4n)

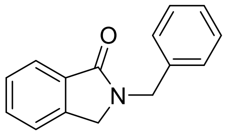

Following the general procedure A and B, the crude product was purified by column chromatography on silica gel (Petroleum ether/EtOAc, 10:1) to yield product **4n** (**A**: 8.0 mg, 24%; **B**: 10.0 mg, 30%) as a colorless oil; ^{1}H NMR ($CDCl_3$, 400 MHz) δ 7.89 (d, J = 6.6 Hz, 1H), 7.53–7.45 (m, 2H), 7.38–7.35 (m, 1H), 7.34–7.27 (m, 5H), 4.80 (s, 2H), 4.26 (s, 2H); ^{13}C NMR ($CDCl_3$, 101 MHz) δ 168.6, 141.3, 137.1, 132.7, 131.5, 128.9 (2C), 128.3 (2C), 128.2, 127.8, 124.0, 122.8, 49.5, 46.5; HRMS (ESI) calcd for $C_{15}H_{14}NO$ 224.1069 ($[M+H]^+$), found 224.1062.

2-Phenethylisoindolin-1-one (4o)

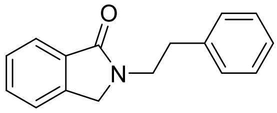

Following the general procedure A and B, the crude product was purified by column chromatography on silica gel (Petroleum ether/EtOAc, 5:1) to yield product **4o** (**A**: 10.2 mg, 29%; **B**: 11.0 mg, 31%) as a colorless oil; ^{1}H NMR ($CDCl_3$, 400 MHz) δ 7.84 (d, J = 7.5 Hz, 1H), 7.49 (td, J = 7.4, 1.3 Hz, 1H), 7.43 (t, J = 7.5 Hz, 1H), 7.36 (d, J = 7.3 Hz, 1H), 7.30–7.26 (m, 2H), 7.25–7.19 (m, 3H), 4.19 (s, 2H),

3.87 (t, J = 7.3 Hz, 2H), 2.99 (t, J = 7.3 Hz, 2H); ^{13}C NMR ($CDCl_3$, 101 MHz) δ 168.6, 141.3, 138.9, 133.0, 131.3, 128.8 (2C), 128.7 (2C), 128.1, 126.6, 123.7, 122.7, 50.7, 44.2, 35.0; HRMS (ESI) calcd for $C_{16}H_{16}NO$ 238.1226 ($[M+H]^+$), found 238.1219.

Reaction procedure for the preparation of compound 2a and 4a using 5.0 mmol scale:

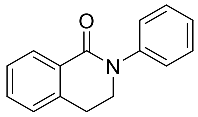

A mixture of substrates **1a** (1.05 g, 5 mmol), DBU (375 μL, 2.5 mmol) and BHT (551 mg, 2.5 mmol) were mixed in a 150 mL two-neck bottle containing a magnetic stirring bar, then adding dry CH_3CN (100 mL). The resulting mixture was stirred at rt with the irradiation of a 72 W blue LED light under O_2 atmosphere. After completion, the reaction mixture was concentrated in vacuum and the crude product was purified using column chromatography on silica gel (Petroleum ether/EtOAc, 10:1) to yield product **2a** (0.88 g, 79%).

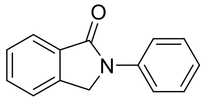

A mixture of substrates **3a** (1.0 g, 5.1 mmol), DBU (380 μL, 2.55 mmol) and BHT (562 mg, 2.55 mmol) were mixed in a 150 mL two-neck bottle containing a magnetic stirring bar, then adding dry CH_3CN (100 mL). The resulting mixture was stirred at rt with the irradiation of a 72 W blue LED light under O_2 atmosphere. After completion, the reaction mixture was concentrated in vacuum and the crude product was purified using column chromatography on silica gel (Petroleum ether/EtOAc, 10:1) to yield product **4a** (0.68 g, 64%).

6. Mechanistic Studies

6.1 Radical Trapping Experiments

Radical trapped experiment (TEMPO):

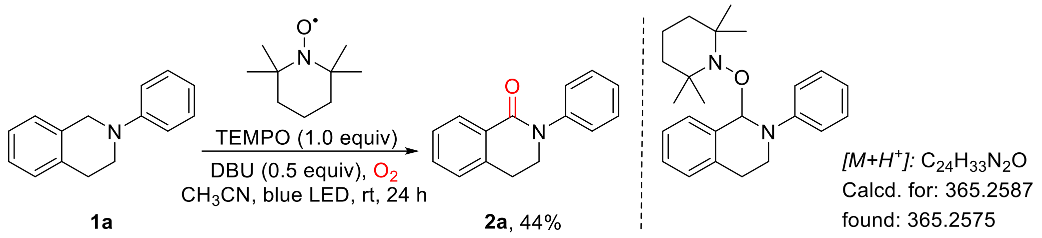

A mixture of **1a** (31.4 mg, 0.15 mmol), DBU (11 μL, 0.075 mmol) and TEMPO (23.4 mg, 0.15 mmol) in a 25 mL Schlenk tube was added CH_3CN (3 mL). The reaction mixture was stirred under O_2 and 72 W blue LED for 24 h. ESI-HRMS analysis of the reaction mixture showed that radical adduct was formed.

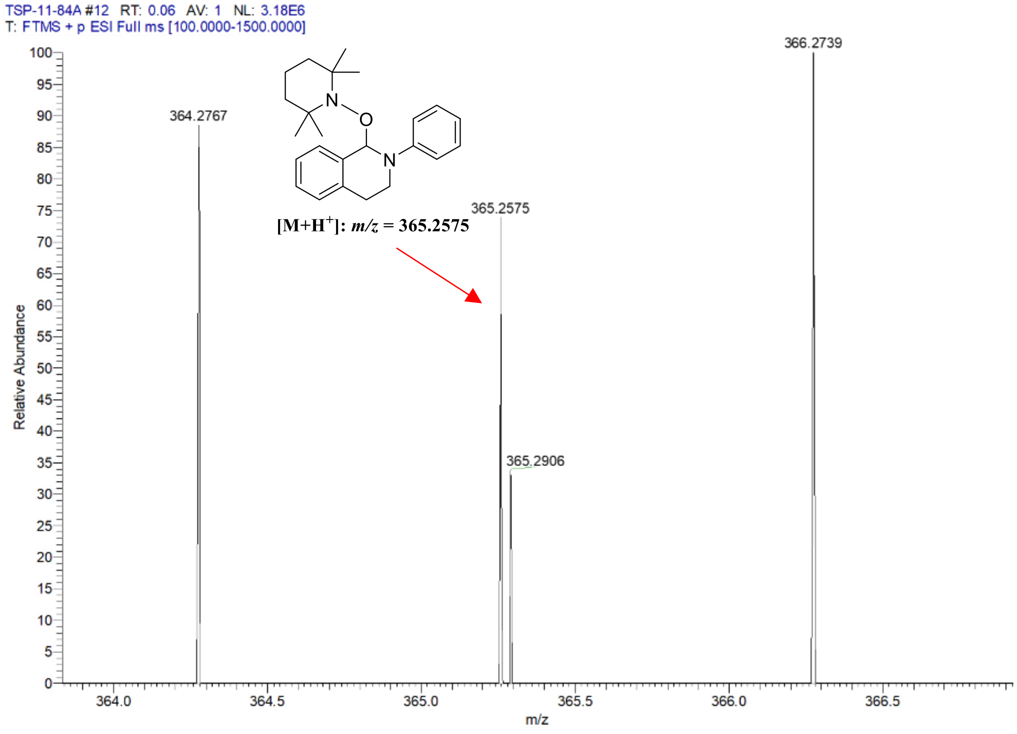

Radical trapped experiment (BHT):

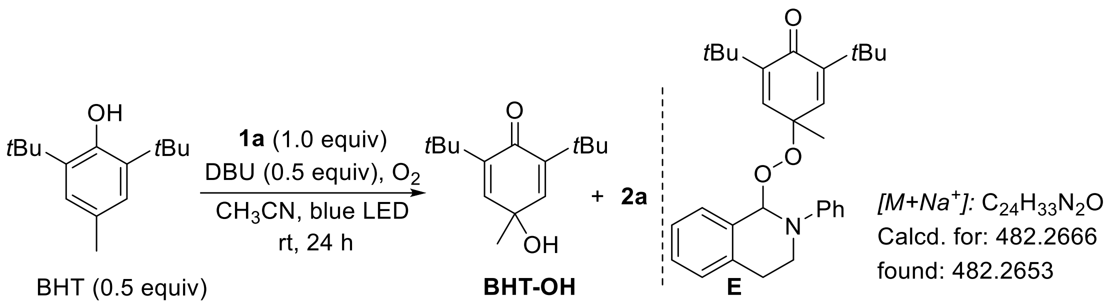

A mixture of **1a** (31.4 mg, 0.15 mmol), DBU (11 μL, 0.075 mmol) and BHT (16.5 mg, 0.075 mmol) in a 25 mL Schlenk tube was added CH_3CN (3 mL). The reaction mixture was stirred under O_2 and 72 W blue LED for 24 h. ESI-HRMS analysis of the reaction mixture showed that radical adduct **E** was formed. In addition, **BHT-OH** (10.3 mg, 61%) was isolated from the reaction mixture using column chromatography on silica gel (Petroleum ether/EtOAc,5:1). ^{1}H NMR ($CDCl_3$, 400 MHz) δ 6.53 (s, 2H), 1.92 (s, 1H), 1.39 (s, 3H), 1.19 (s, 18H); ^{13}C NMR ($CDCl_3$, 101 MHz) δ 186.2, 145.5 (2C), 143.3 (2C), 67.5, 34.6 (2C), 29.5 (6C), 28.1; HRMS (ESI) calcd for $C_{15}H_{25}O_2$ 237.1849 ($[M+H]^+$), found 237.1844.

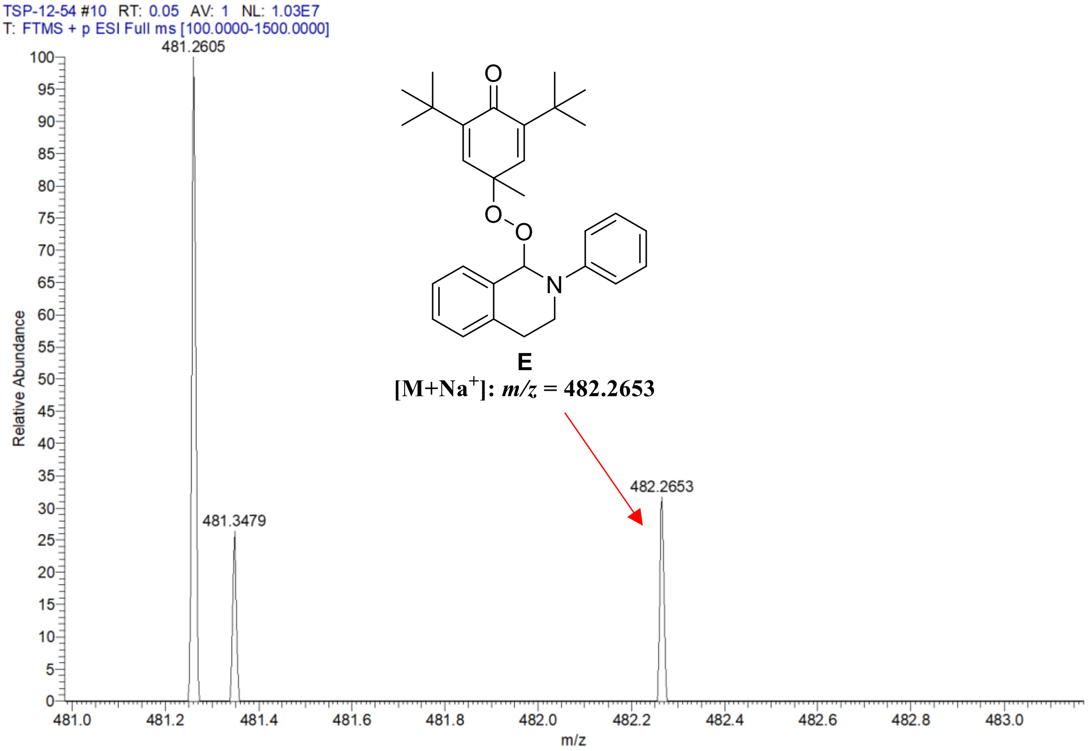

6.2 ^{18}O Labeling Experiments

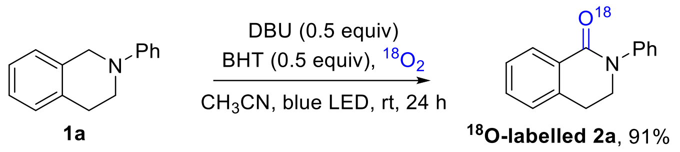

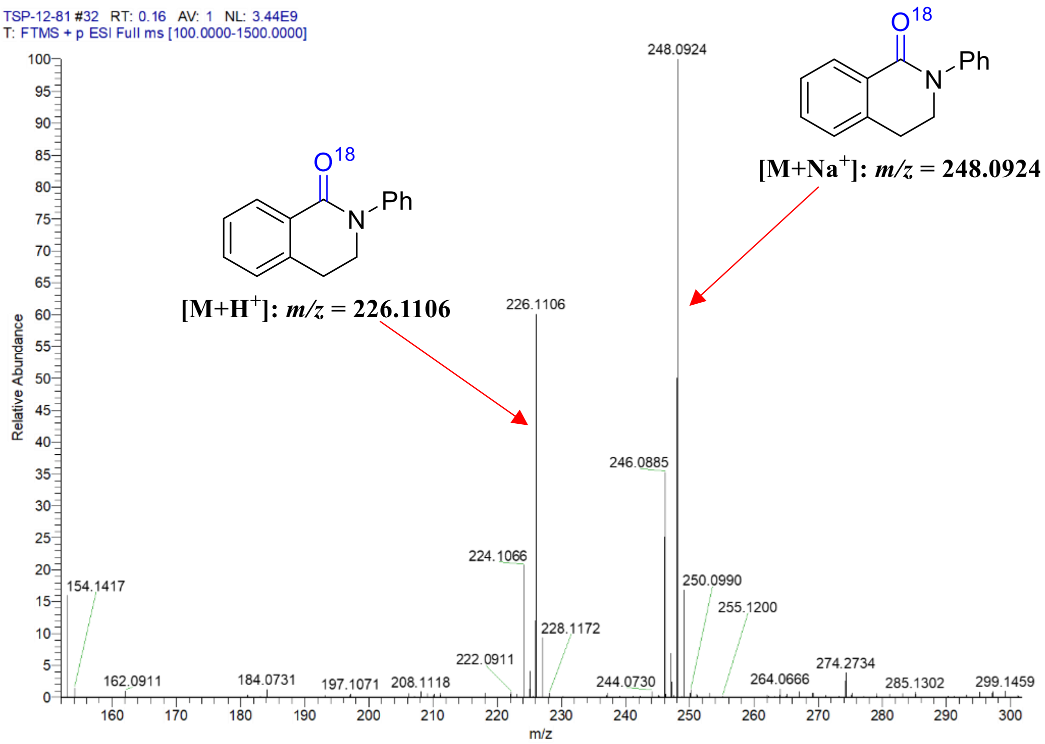

6.3 UV-Vis Absorption and Fluorescence Properties

All UV/vis absorption and fluorescence spectra were recorded using a MD SpectraMax Plus384 and a HITACHI F-4700. Stock solutions of **1** and **3** (0.1 mM) were prepared in CH_3CN. Time-resolved photoluminescence spectra were measured at room temperature by using a laser-scanned fluorescence lifetime/intensity imaging system (FLRM300, Time-Tech Spectra, LLC) with a 365 nm picosecond pulsed diode laser as an excitation source.

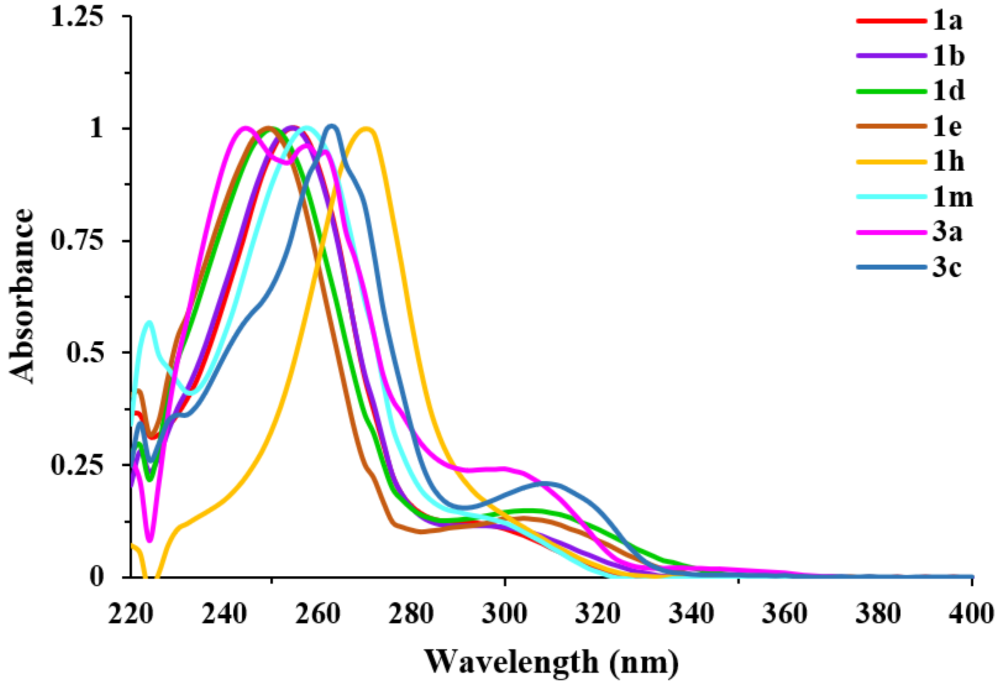

Figure S2 UV/vis absorption spectra of **1a, 1b, 1d, 1e, 1h, 1m, 3a** and **3c** (0.2 μM) in CH_3CN.

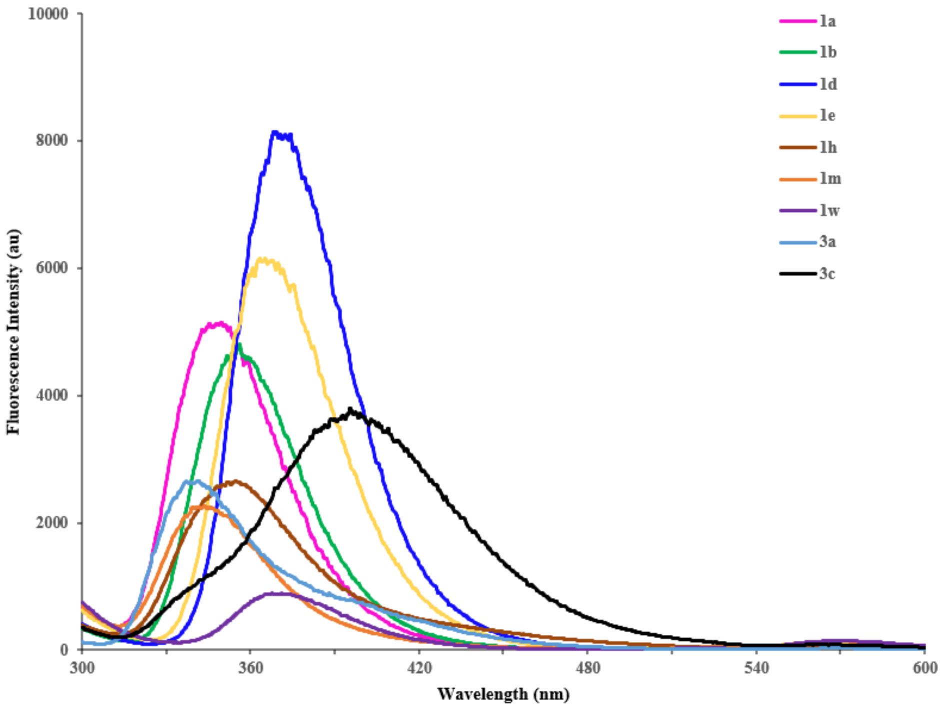

Figure S3 Fluorescence emission spectra of **1a, 1b, 1d, 1e, 1h, 1m, 1w, 3a** and **3c** (0.2 μM) in CH_3CN (No normalization).

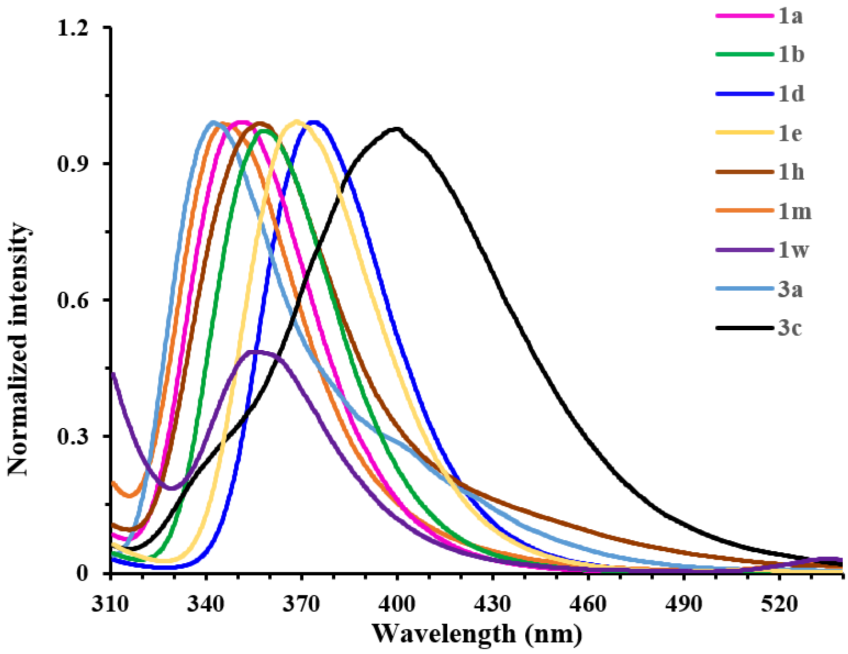

Figure S4 Normalized fluorescence emission spectra of **1a, 1b, 1d, 1e, 1h, 1m, 1w, 3a** and **3c** (0.2 μM) in CH_3CN (Normalization).

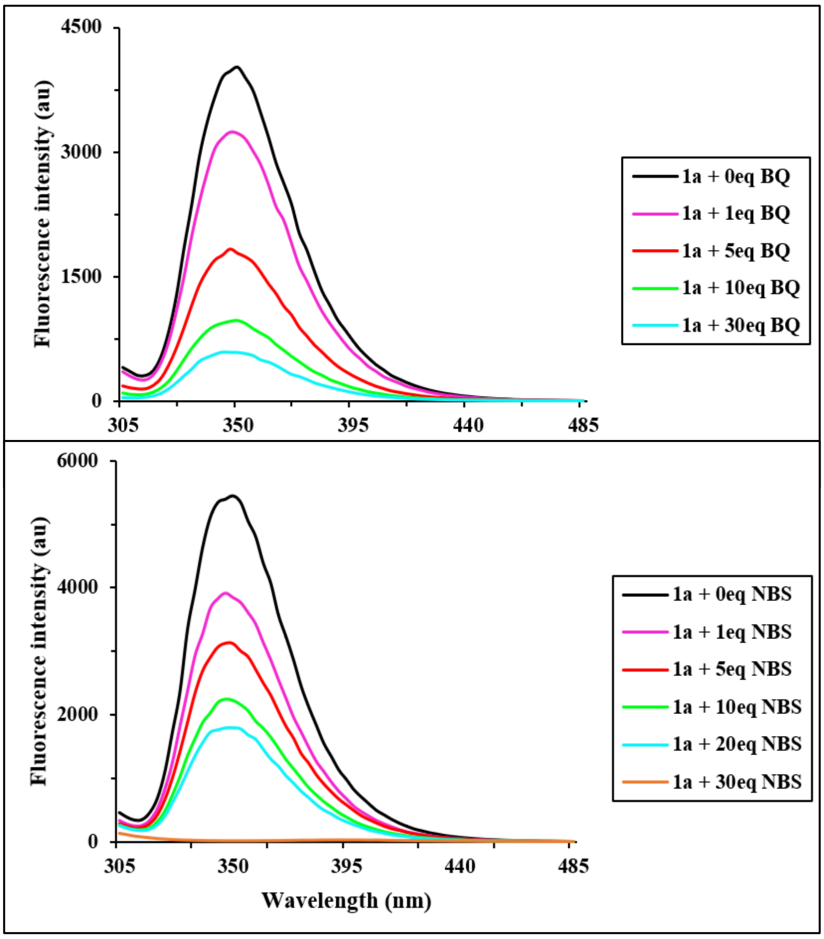

Figure S5 Fluorescence spectra of **1a** (0.2 μM) with different concentrations of BQ (0, 1, 5, 10 or 30 equiv) or NBS (0, 1, 5, 10, 20 or 30 equiv) in CH_3CN (excitation at 254 nm).

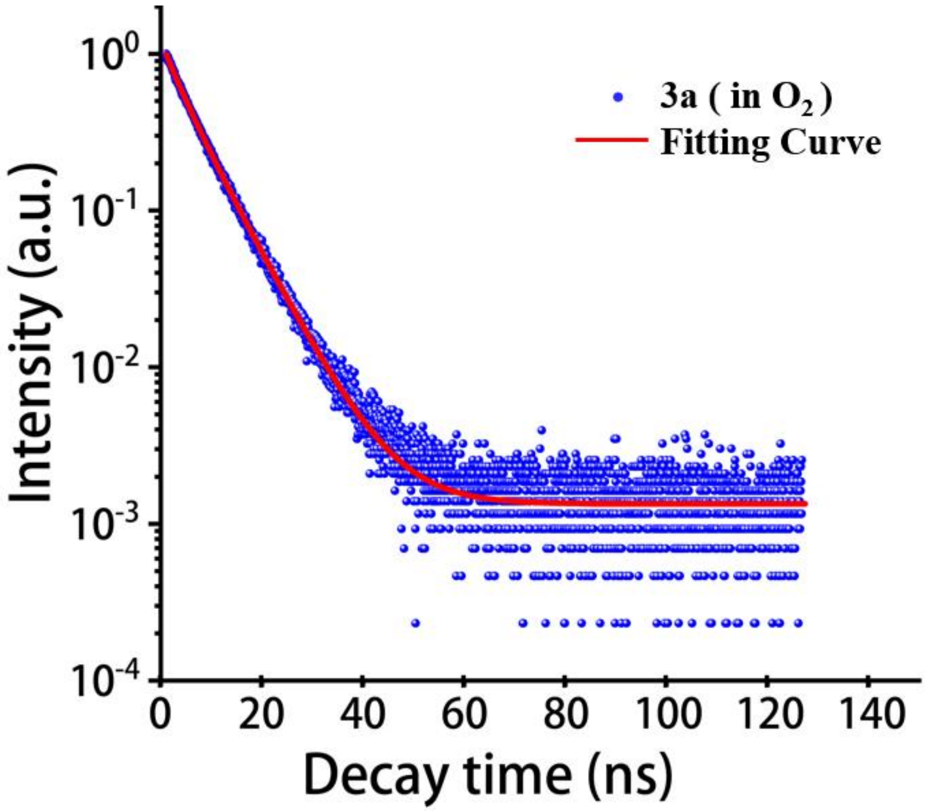

Figure S6 Time-dependent delayed fluorescence lifetime decay spectra of **3a** in O_2.

Parameter	Value	Std. Dev.
y0	0.002	8.23636E-5
A_1	0.600	0.02077
τ_1	2.7440	0.1262
A_2	0.002	0.02119
τ_2	7.2085	0.07228

$$\tau_{avg} = (A_1\tau_1^2 + A_2\tau_2^2)/(A_1\tau_1 + A_2\tau_2) = 2.7 \text{ ns}$$

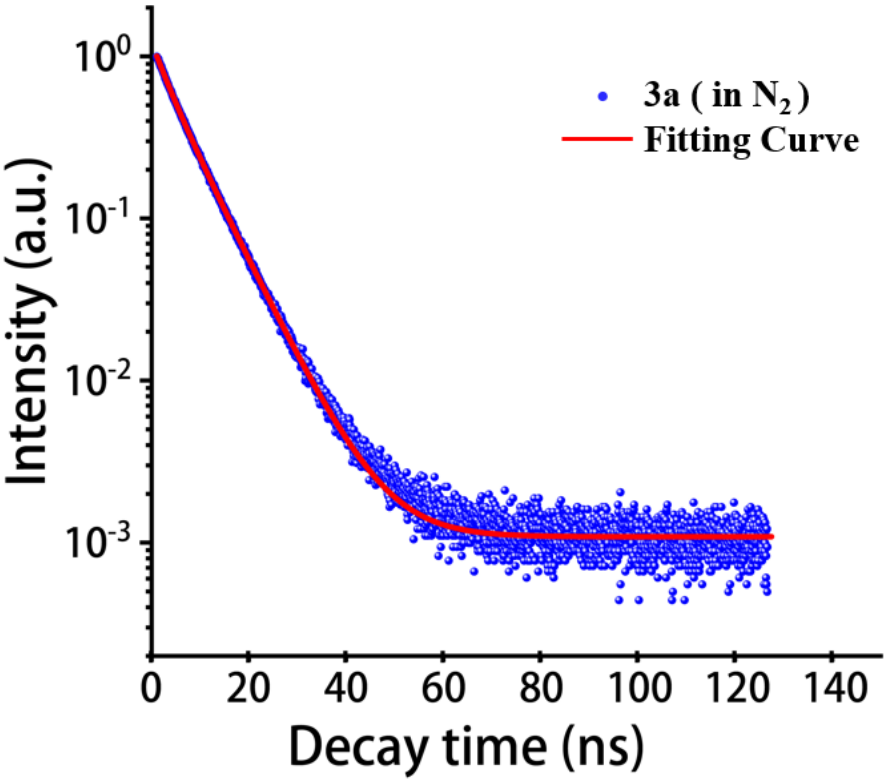

Figure S6 Time-dependent delayed fluorescence lifetime decay spectra of **3a** in N_2.

Parameter	Value	Std. Dev.
y0	0.00109	4.06374E-5
A_1	0.23972	0.00866
τ_1	3.26496	0.0618
A_2	0.76844	0.00887
τ_2	7.16059	0.02975

$$\tau_{avg} = (A_1\tau_1^2 + A_2\tau_2^2)/(A_1\tau_1 + A_2\tau_2) = 6.8 \text{ ns}$$

7. Applications in the Synthesis of Indoprofen and 8-Oxoberber ine

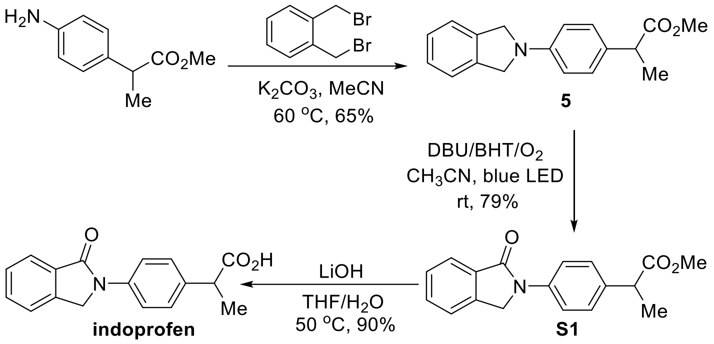

Follow the procedure reported previously[44], methyl 2-(4-aminophenyl)propanoate (358.4 mg, 2.0 mmol), 1,2-bis(bromomethyl) benzene (527.9 mg, 2.0 mmol) and K_2CO_3 (828 mg, 6.0 mmol) were added in CH_3CN (5 mL) under N_2 atmosphere. The reaction mixture was heated to 60 °C (oil bath) for 20 h, and then cooled to rt. The reaction was quenched by water (10 mL) and extracted with EA (30 mL) three times. The combined organic phases were washed with brine, dried over anhydrous Na_2SO_4 and concentrated in vacuo. The crude product was purified by silica gel chromatography (Petroleum ether/EtOAc, 10:1) to furnish the product **5** (562.7 mg, 65%) as a white solid.

A mixture of **5** (50 mg, 0.18 mmol), DBU (13 μL, 0.09 mmol), and BHT (19.6 mg, 0.09 mmol) were mixed in a 25 mL Schlenk tube containing a magnetic stirring bar, then adding dry CH_3CN (3.6 mL). The resulting mixture was stirred at rt with the irradiation of a 72 W blue LED light under O_2 atmosphere. After completion, the reaction mixture was concentrated in vacuum and the crude product was purified by flash column chromatography on silica gel (Petroleum ether/EtOAc, 5:1) to yield product **S1** (42.1 mg, 79%). ^{1}H NMR ($CDCl_3$, 400 MHz) δ 7.89 (d, J = 7.4 Hz, 1H), 7.82–7.79 (m, 2H), 7.59–7.55 (m, 1H), 7.50–7.46 (m, 2H), 7.36–

7.32 (m, 2H), 4.82 (s, 2H), 3.72 (q, *J* = 7.2 Hz, 1H), 3.65 (s, 3H), 1.50 (d, *J* = 7.2 Hz, 3H); ^{13}C NMR ($CDCl_3$, 101 MHz) δ 175.0, 167.5, 140.2, 138.6, 136.7, 133.2, 132.2, 128.5, 128.3 (2C), 124.2, 122.7, 119.8 (2C), 52.2, 50.8, 44.9, 18.6; HRMS (ESI) calcd for $C_{18}H_{18}NO_3$ 296.1281 ($[M+H]^+$), found 296.1277.

S1 (30 mg, 0.1 mmol) was dissolved in a 1:1 mixture of THF and water (4 mL), followed by addition of the LiOH (19.1 mg, 0.8 mmol). The reaction was heated up to 50 °C (oil bath) for 10 h. Reaction mixture was then cooled to 0 °C, quenched with 15 mL of 10% aqueous HCl, extracted with ethyl acetate (20 mL), washed with brine, dried over Na_2SO_4. The organic layer was concentrated in vacuum. The desired product **indoprofen** (28.1 mg, 90%) was obtained as a white solid; m.p. 213-214 °C; ^{1}H NMR ($CDCl_3$, 400 MHz) δ 7.91 (d, *J* = 7.2 Hz, 1H), 7.83 (d, *J* = 8.6 Hz, 2H), 7.61–7.57 (m, 1H), 7.51 (d, *J* = 7.4 Hz, 2H), 7.38(d, *J* = 8.7 Hz, 2H), 4.84 (s, 2H), 3.77 (q, *J* = 7.1 Hz, 1H), 1.53 (d, *J* = 7.2 Hz, 3H); ^{13}C NMR ($CDCl_3$, 101 MHz) δ 177.9, 167.6, 140.2, 138.8, 136.0, 133.3, 132.2, 128.5 (2C), 128.5, 124.3, 122.7, 119.8 (2C), 50.8, 44.5, 18.3.; HRMS (ESI) calcd for $C_{17}H_{16}NO_3$ 282.1130 ($[M+H]^+$), found 282.1125.

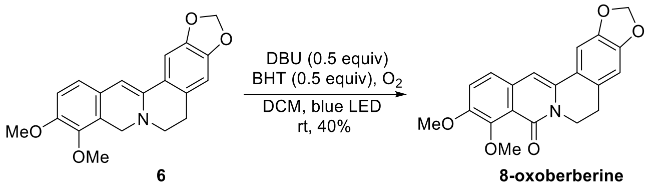

A mixture of **6** (51 mg, 0.15 mmol), DBU (11 μL, 0.075 mmol), and BHT (16.5 mg, 0.075 mmol) were mixed in a 25 mL Schlenk tube containing a magnetic stirring bar, then adding dry DCM (3 mL). The resulting mixture was stirred at rt with the irradiation of a 72 W blue LED light under O_2 atmosphere. After completion, the reaction mixture was concentrated in vacuum and the crude product was purified by flash column chromatography on silica gel (Petroleum

ether/EtOAc, 1:1) to yield **8-oxoberberine** (21 mg, 40%). ^{1}H NMR ($CDCl_3$, 400 MHz) δ 7.31 (d, J = 8.7 Hz, 1H), 7.26 (d, J = 8.7 Hz, 1H), 7.20 (s, 1H), 6.70 (s, 1H), 6.69 (s, 1H), 5.99 (s, 2H), 4.28 (t, J = 6.0 Hz, 2H), 4.00 (s, 3H), 3.94 (s, 3H), 2.88 (t, J = 6.0 Hz, 2H); ^{13}C NMR ($CDCl_3$, 101 MHz) δ 160.2, 151.5, 149.6, 148.5, 147.4, 135.7, 132.4, 130.1, 123.8, 122.4, 119.5, 119.1, 108.0, 104.8, 101.5, 101.4, 61.7, 56.9, 39.4, 28.8; HRMS (ESI) calcd for $C_{20}H_{18}NO_5$ 352.1179 ($[M+H]^+$), found 352.1168.

8. Reference

(37) W. Zhang, S. Yang and Z. Shena. *Adv. Synth. Catal.*, 2016, **358**, 2392–2397.

(38) M. Shigeno, K. Hayashi, K. Nozawa-Kumada and Y. Kondo. *Org. Lett.* 2019, **21**, 5505–5508.

(39) C. Xu, Z. Zhu and Y. Wang, *J Org Chem.*, 2019, **84**, 2234–2242.

(40) G. Oss, S. D. de Vos, K. N. H. Luc, J. B. Harper and T. V. Nguyen. *J Org Chem.*, 2018, **83**, 1000–1010.

(41) M. Asamdi, P. M. Chauhan, J. J. Patel and K. H. Chikhalia. *Tetrahedron*, 2019, **75**, 3485–3494.

(42) T. Wen, B. Liang, J. Liang, D. Wang, J. Shi, S. Xu, W. Zhu, X. Chen and Z. Zhu. *J Org Chem.*, 2022, **87**, 12214–12224.

(43) Q. Wang, X. Tao, S. Ni, Y. Pan and Y. Wang. *Org. Lett.* 2023, **25**, 5822–5826.

(11) P. Thapa, E. Corral, S Sardar, B. S. Pierce and F. W. Foss, *J Org Chem.*, 2019, **84**, 1025–1034.

(44) Z. Shen, Z. Ni, S. Mo, J. Wang and Y. Zhu. *Chem. Eur. J.*, 2012, **18**, 4859–4865.

9. ^{1}H NMR and ^{13}C NMR Spectra

^{1}H NMR spectrum of 1z (400 MHz, CDCl$_3$)

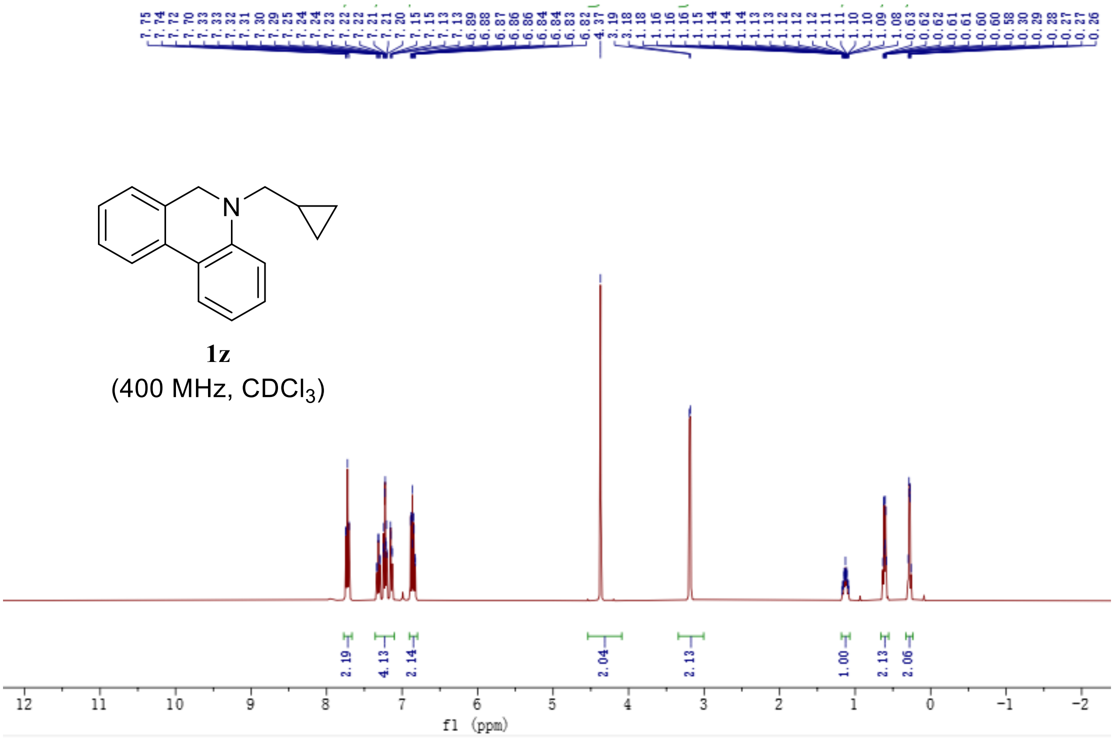

^{13}C NMR spectrum of 1z (101 MHz, CDCl$_3$)

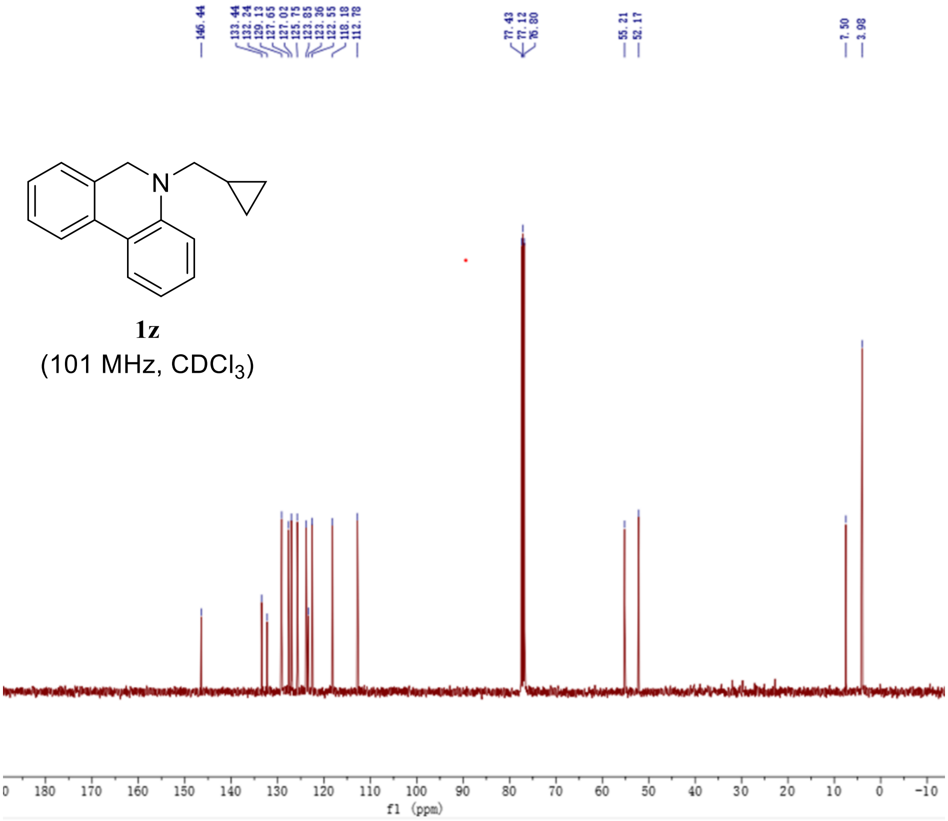

^{1}H NMR spectrum of 3k (400 MHz, $CDCl_3$)

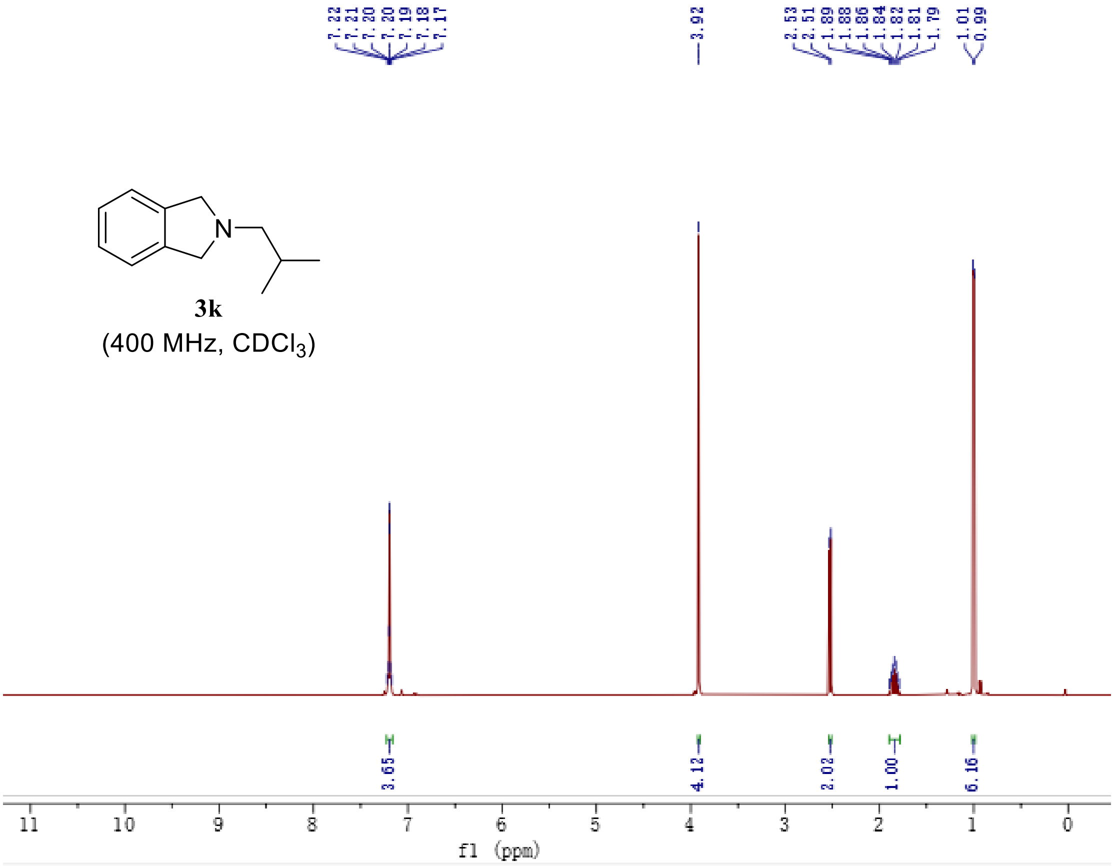

^{13}C NMR spectrum of 1z (101 MHz, $CDCl_3$)

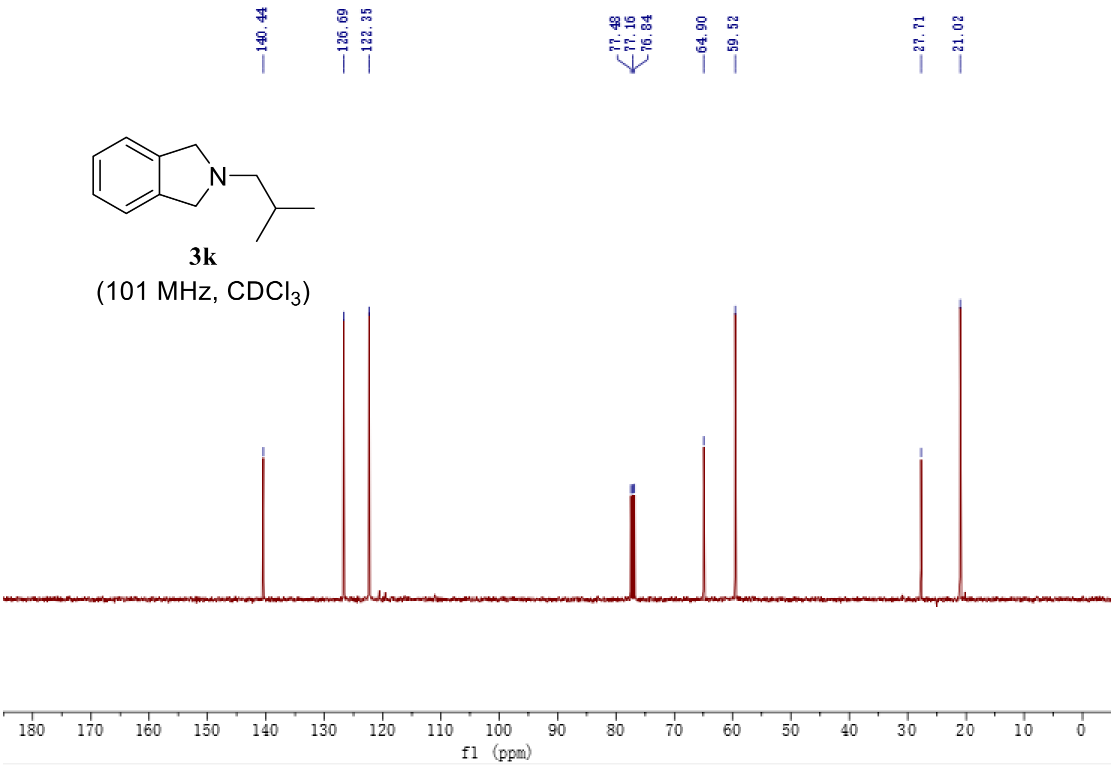

^{1}H NMR spectrum of 2a (400 MHz, $CDCl_3$)

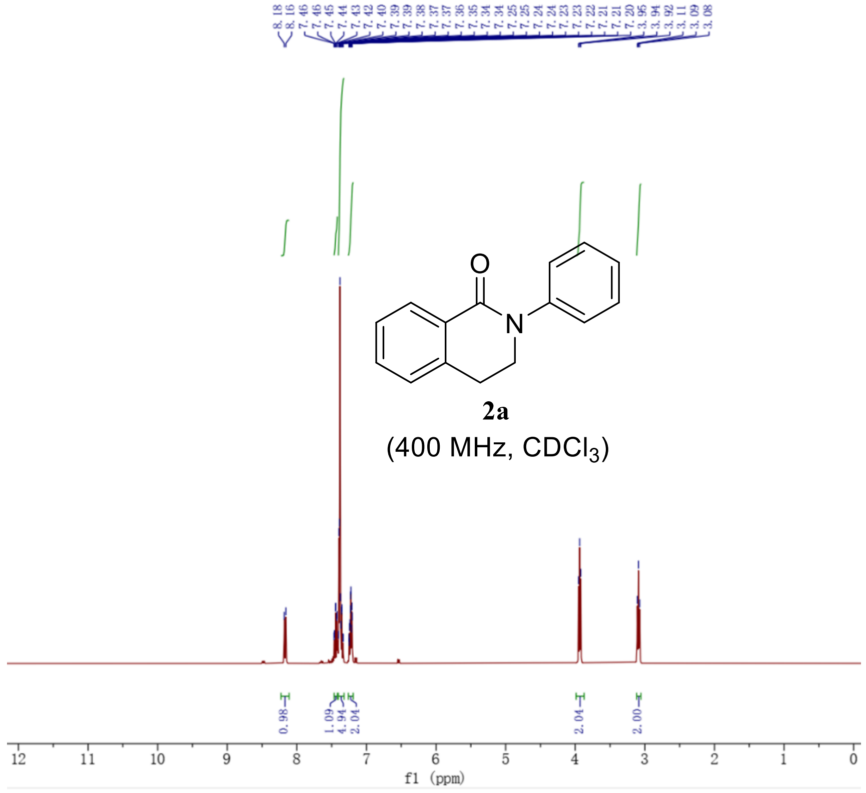

^{13}C NMR spectrum of 2a (101 MHz, $CDCl_3$)

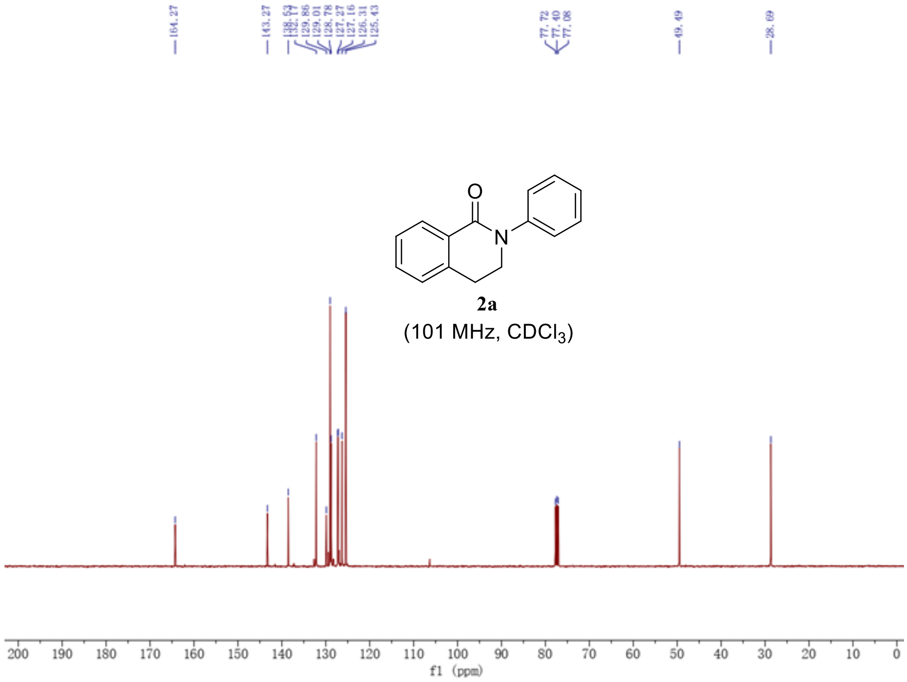

^{1}H NMR spectrum of 2b (400 MHz, $CDCl_3$)

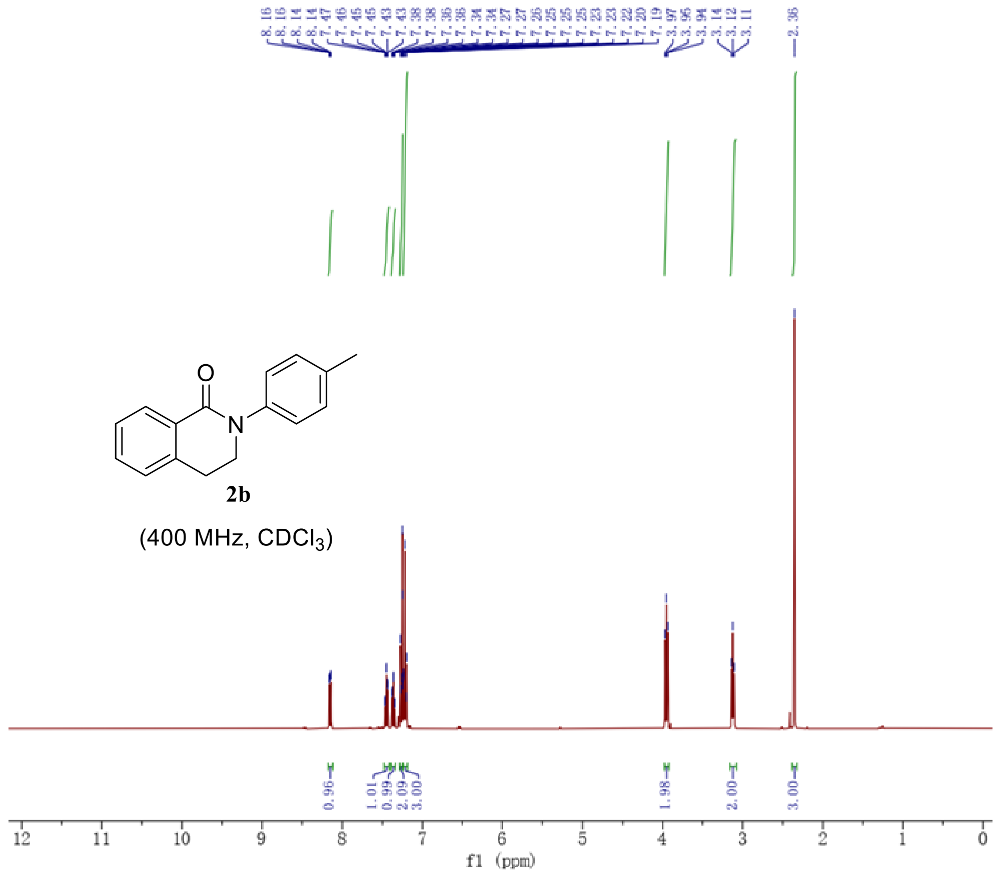

^{13}C NMR spectrum of 2b (101 MHz, $CDCl_3$)

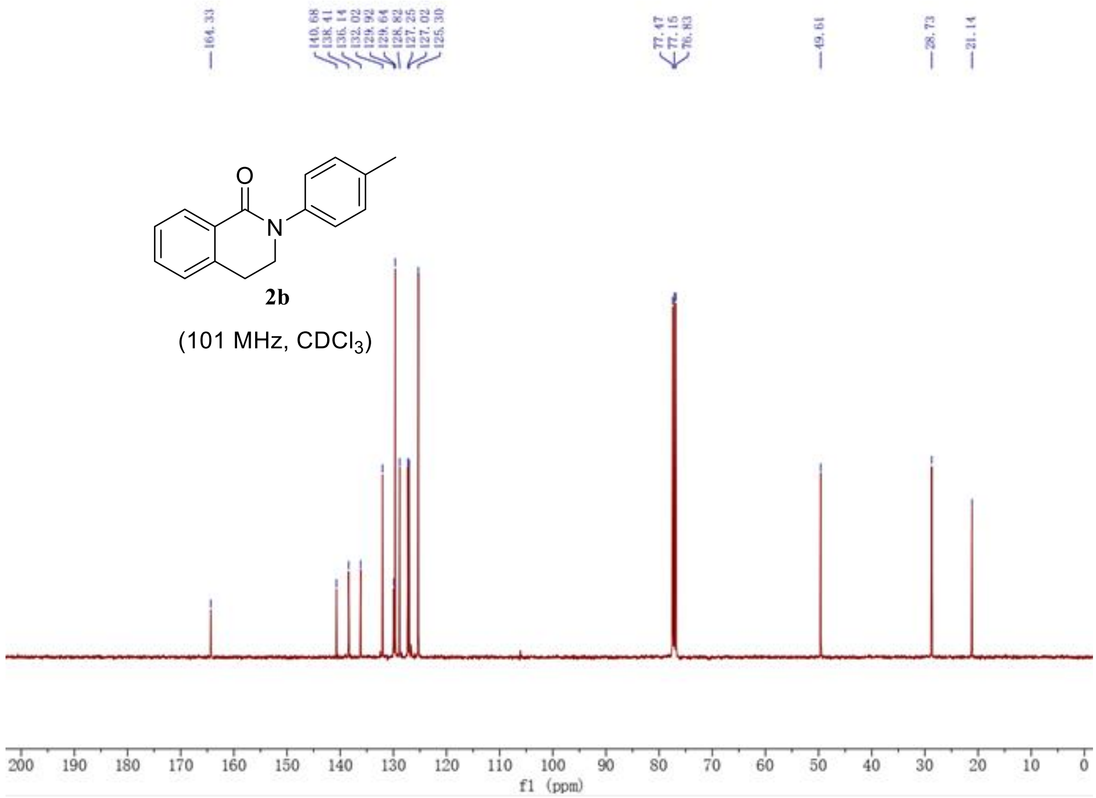

^{1}H NMR spectrum of 2c (400 MHz, $CDCl_3$)

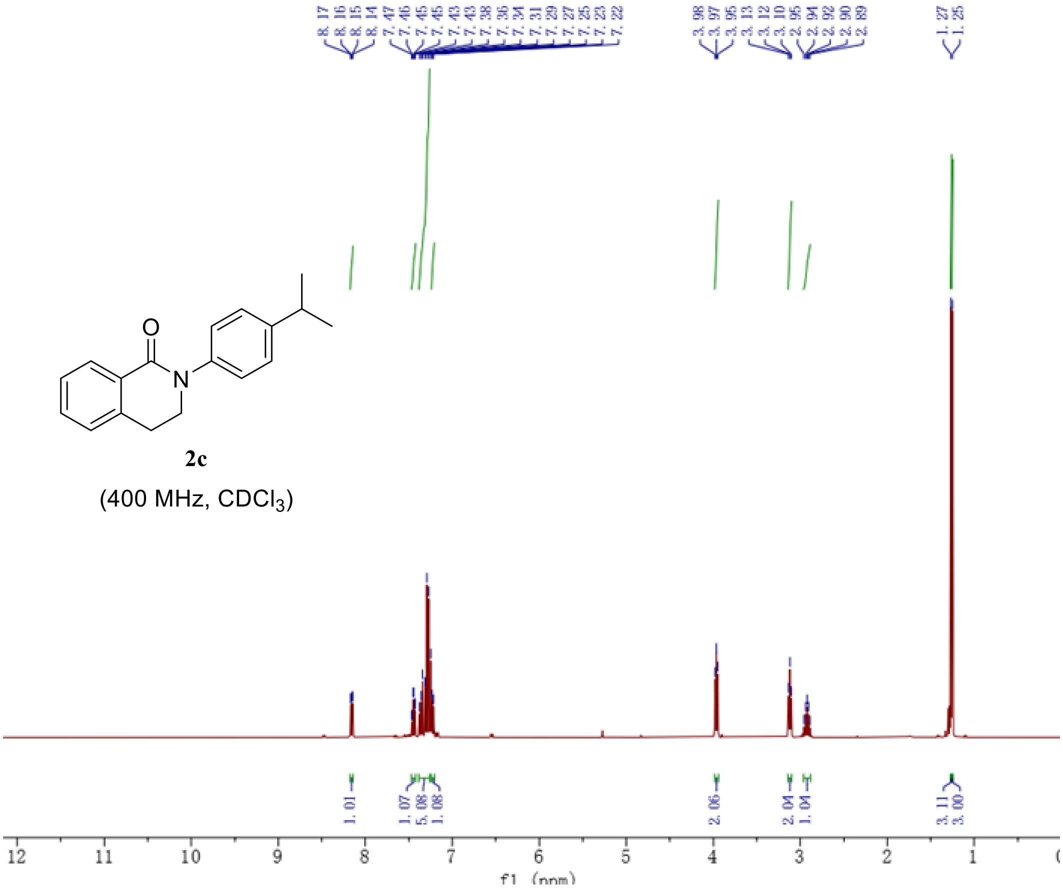

^{13}C NMR spectrum of 2c (101 MHz, $CDCl_3$)

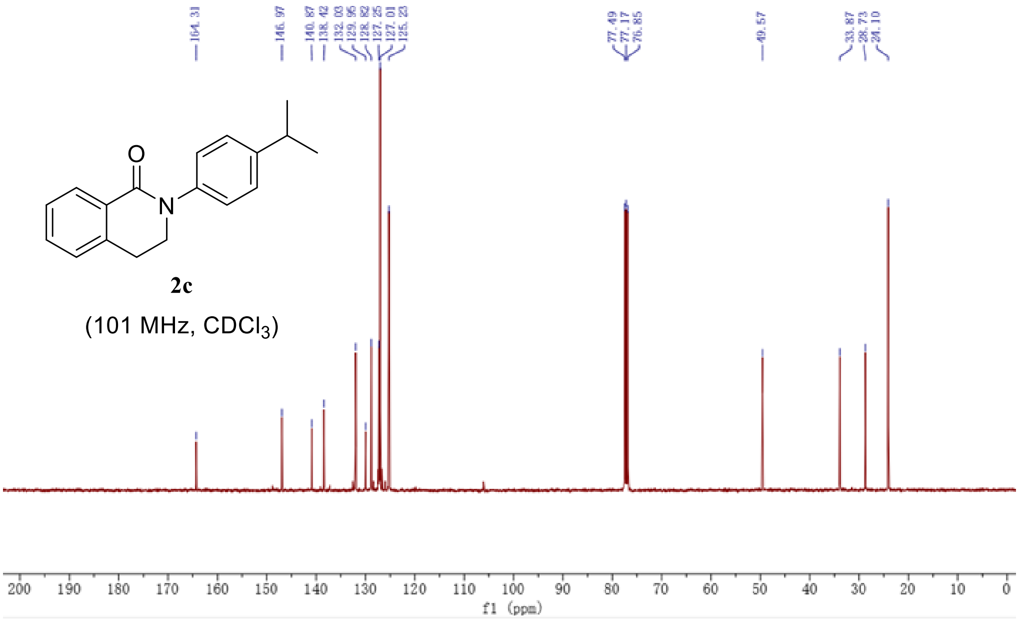

^{1}H NMR spectrum of 2d (400 MHz, $CDCl_3$)

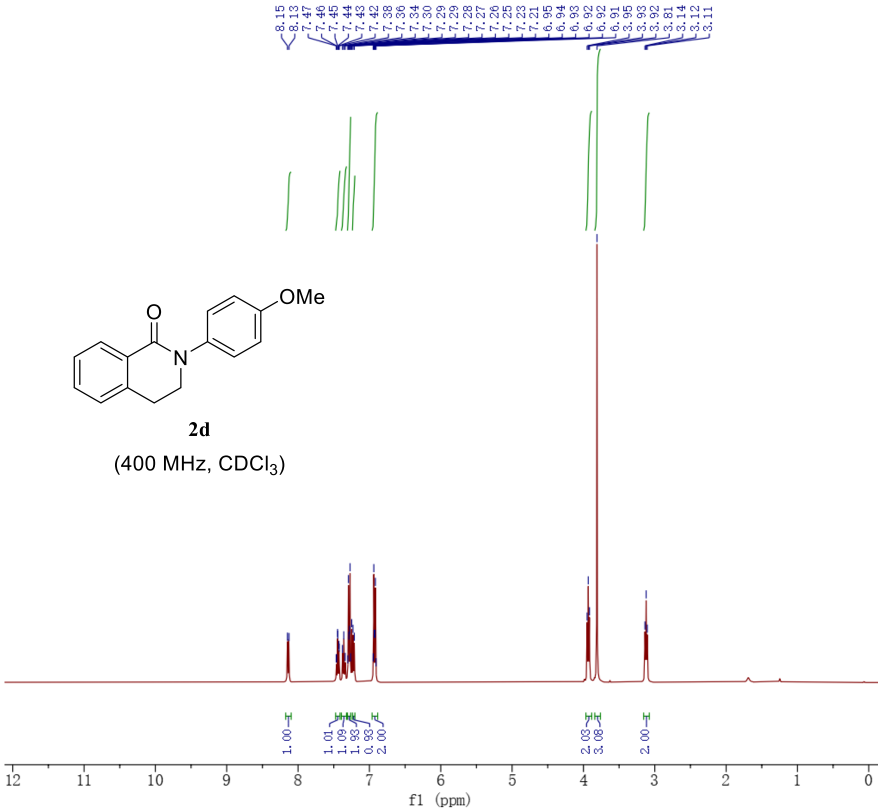

^{13}C NMR spectrum of 2d (101 MHz, $CDCl_3$)

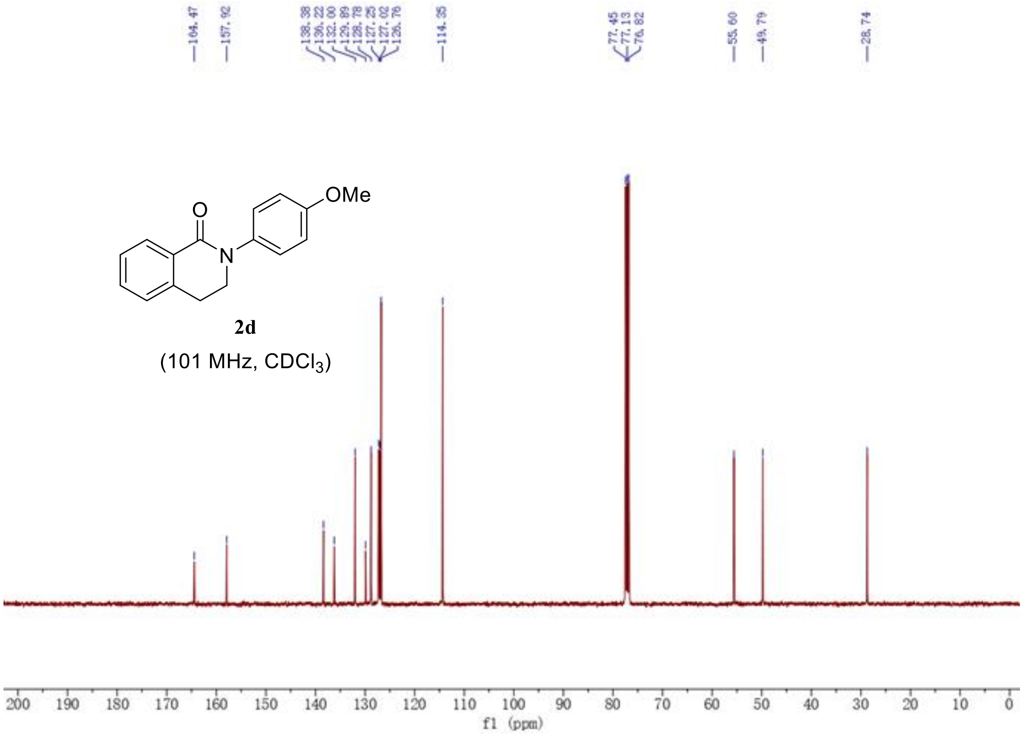

^{1}H NMR spectrum of 2e (400 MHz, $CDCl_3$)

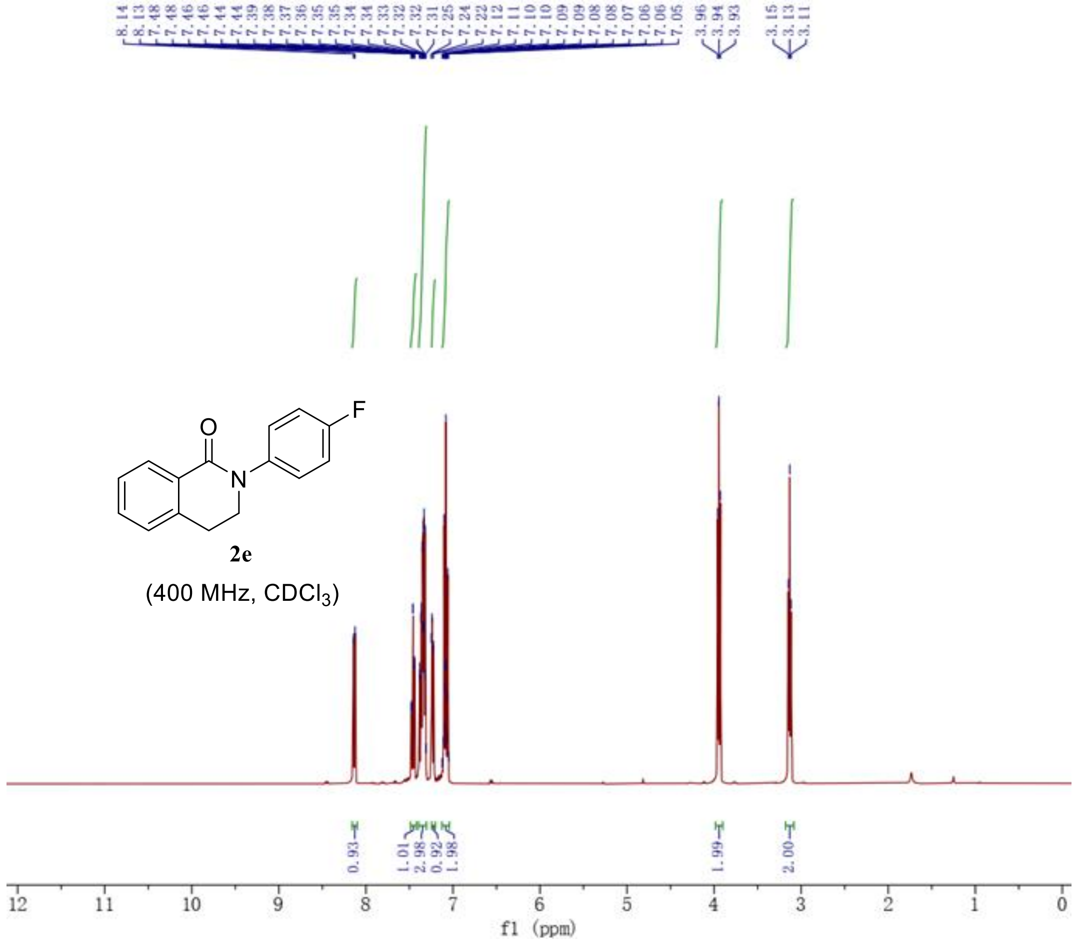

^{13}C NMR spectrum of 2e (101 MHz, $CDCl_3$)

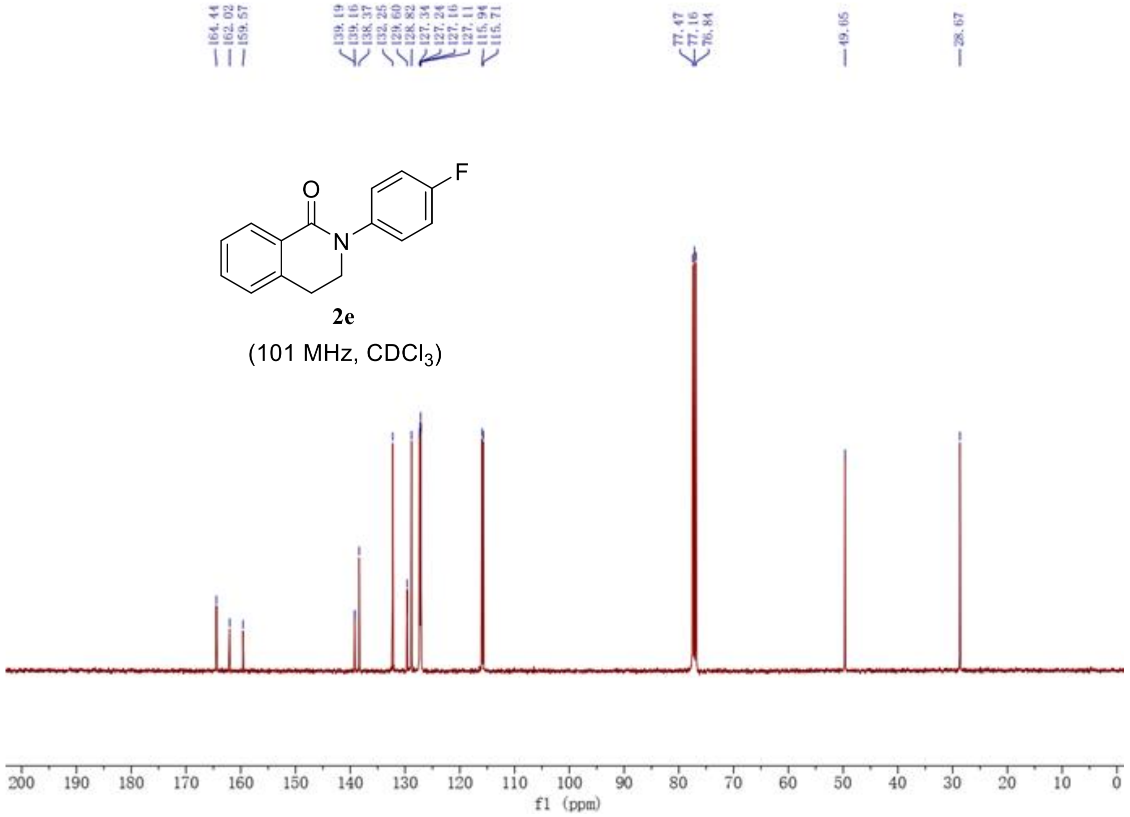

^{1}H NMR spectrum of 2f (400 MHz, $CDCl_3$)

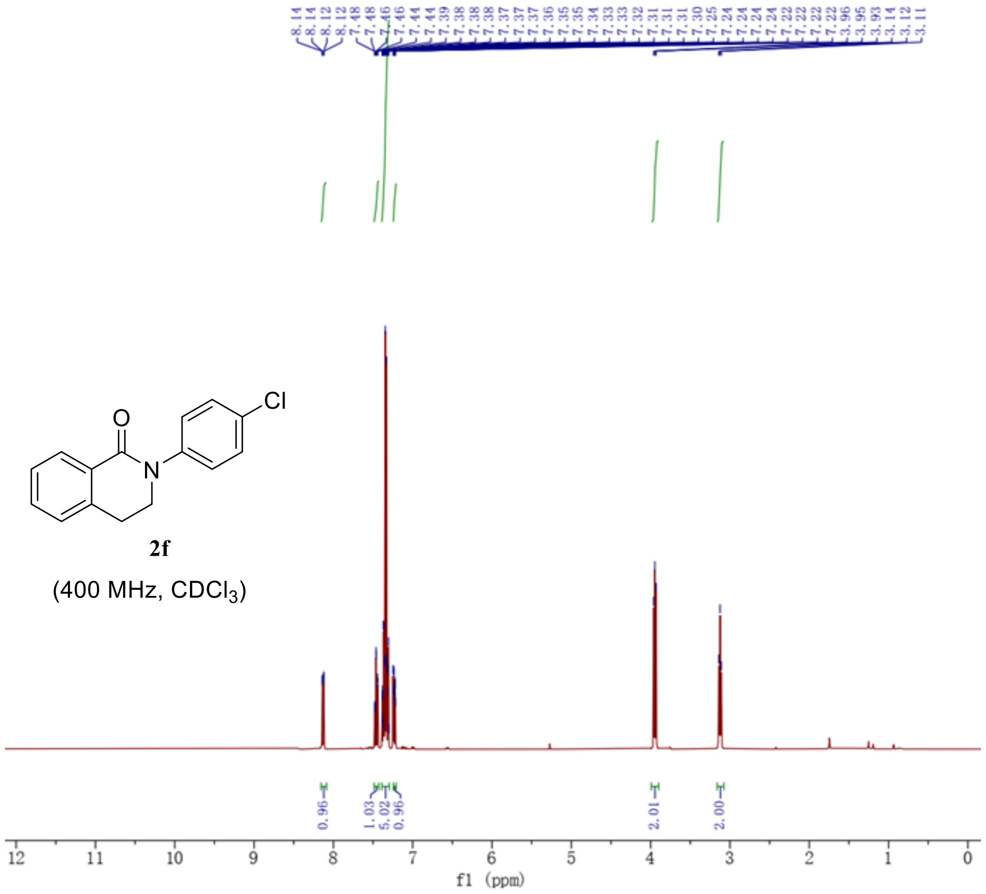

^{13}C NMR spectrum of 2f (101 MHz, $CDCl_3$)

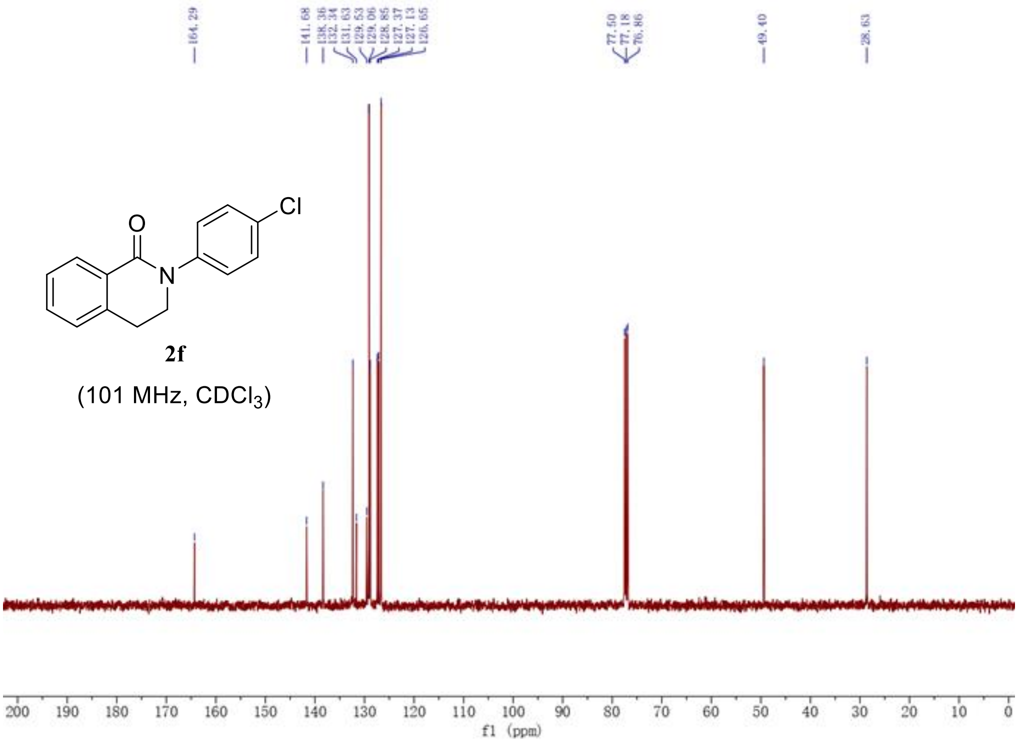

^{1}H NMR spectrum of 2g (400 MHz, $CDCl_3$)

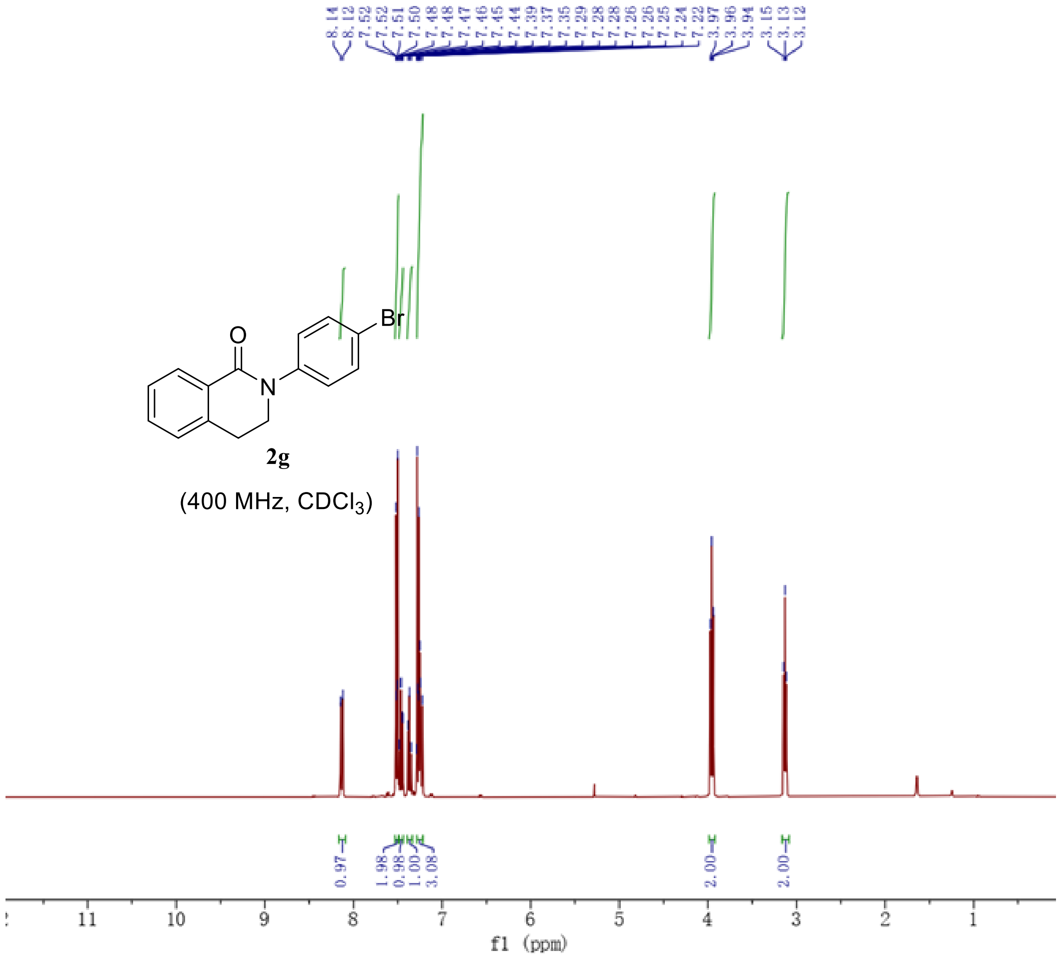

^{13}C NMR spectrum of 2g (101 MHz, $CDCl_3$)

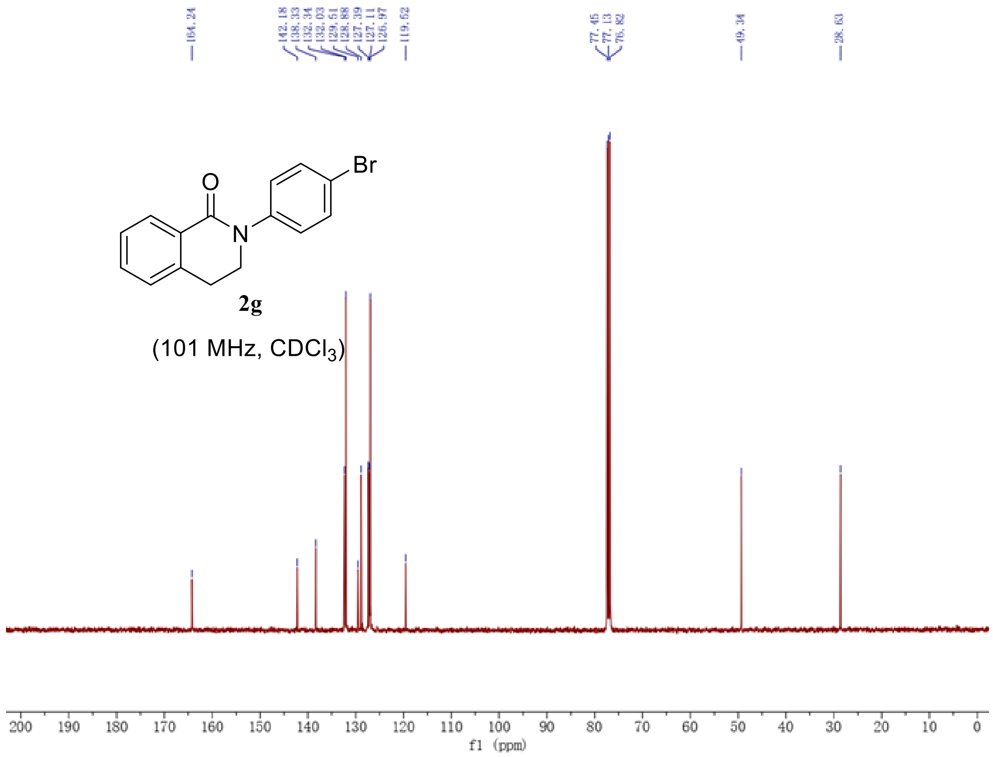

^{1}H NMR spectrum of 2h (400 MHz, $CDCl_3$)

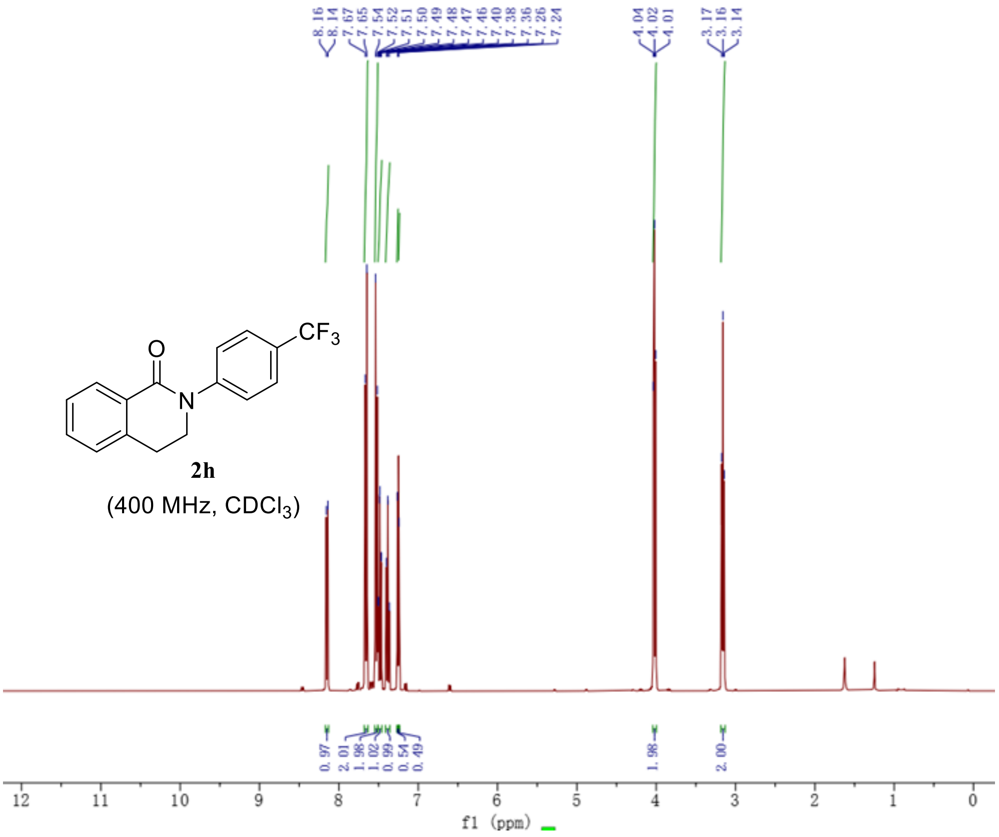

^{13}C NMR spectrum of 2h (101 MHz, $CDCl_3$)

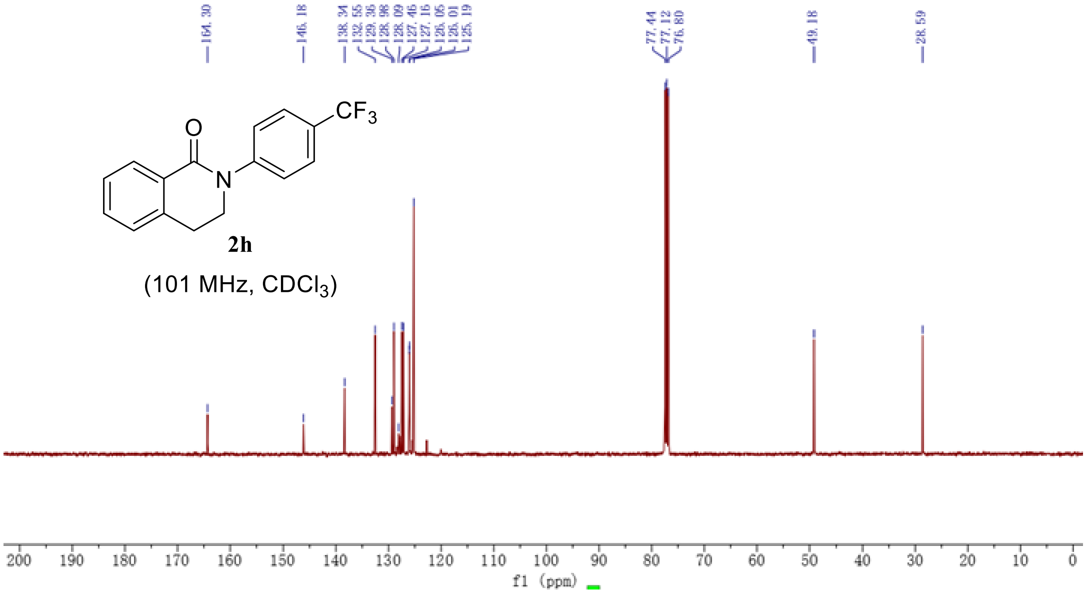

^{1}H NMR spectrum of 2i (400 MHz, $CDCl_3$)

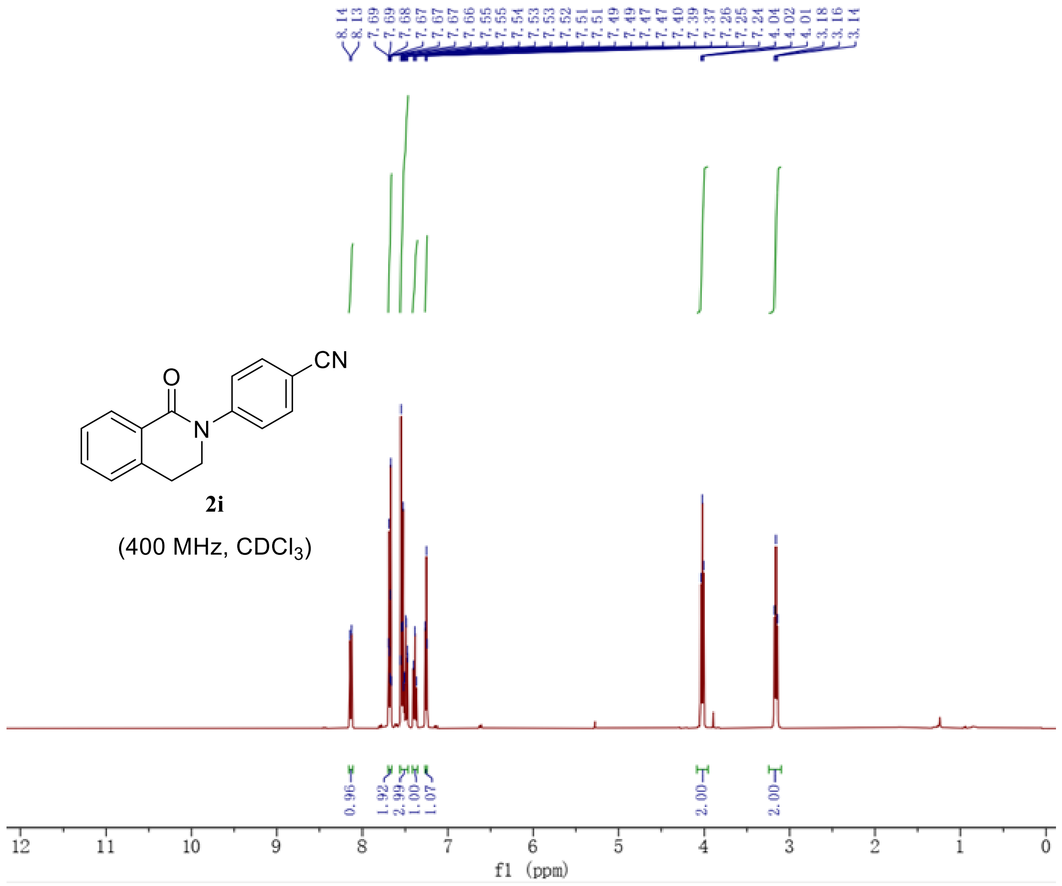

^{13}C NMR spectrum of 2i (101 MHz, $CDCl_3$)

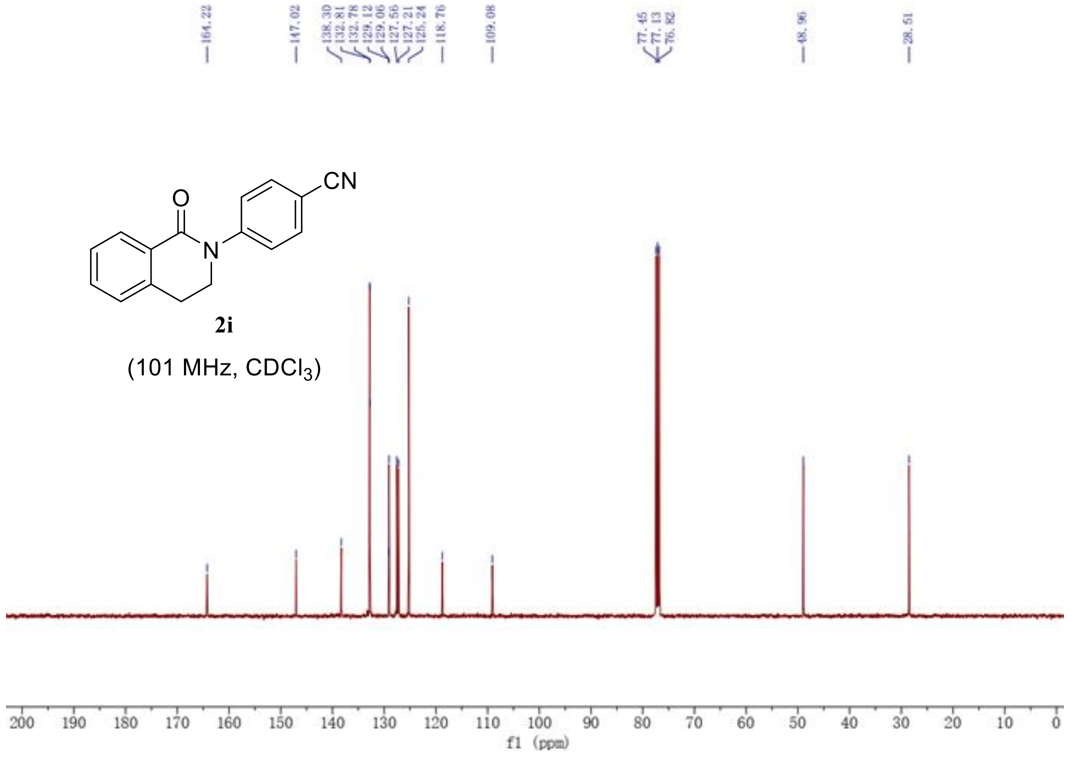

^{1}H NMR spectrum of 2j (400 MHz, $CDCl_3$)

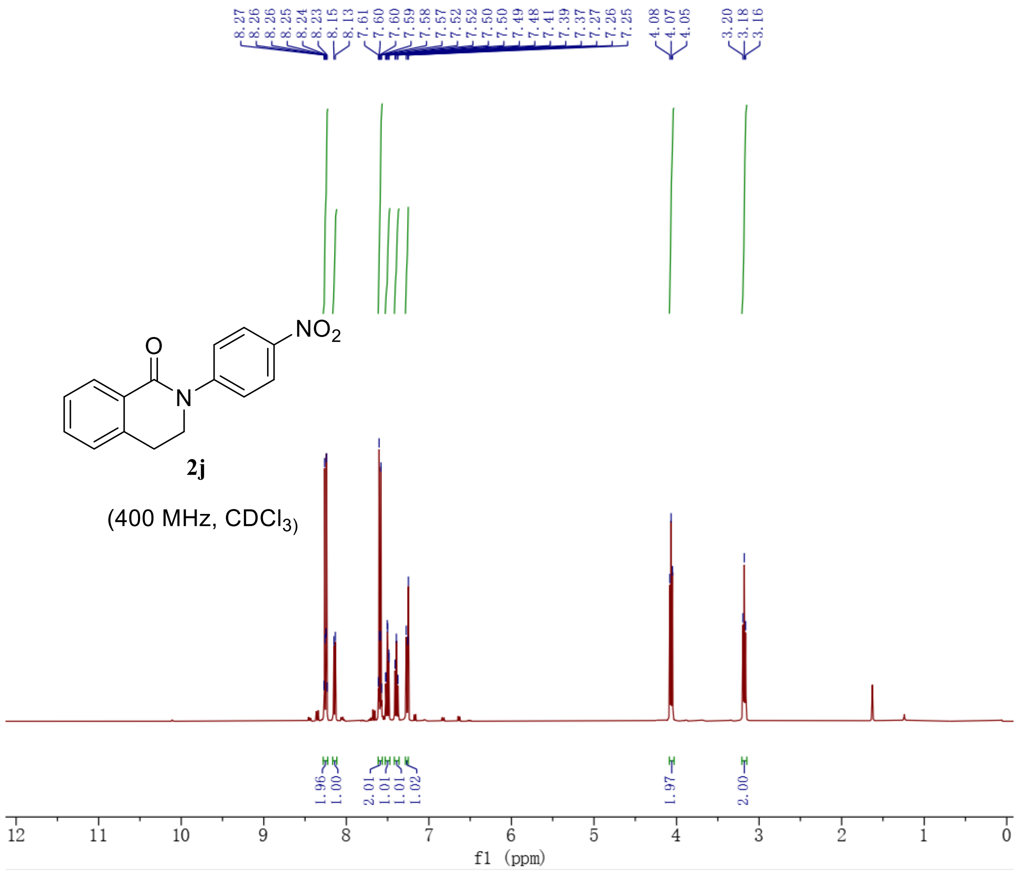

^{13}C NMR spectrum of 2j (101 MHz, $CDCl_3$)

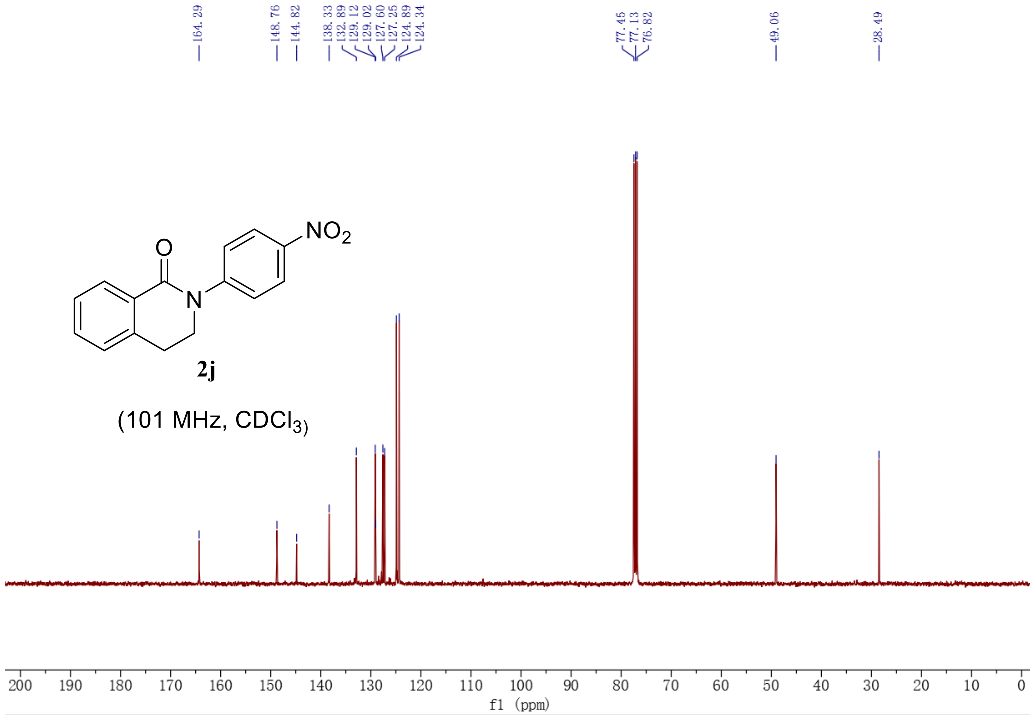

^{1}H NMR spectrum of 2k (400 MHz, $CDCl_3$)

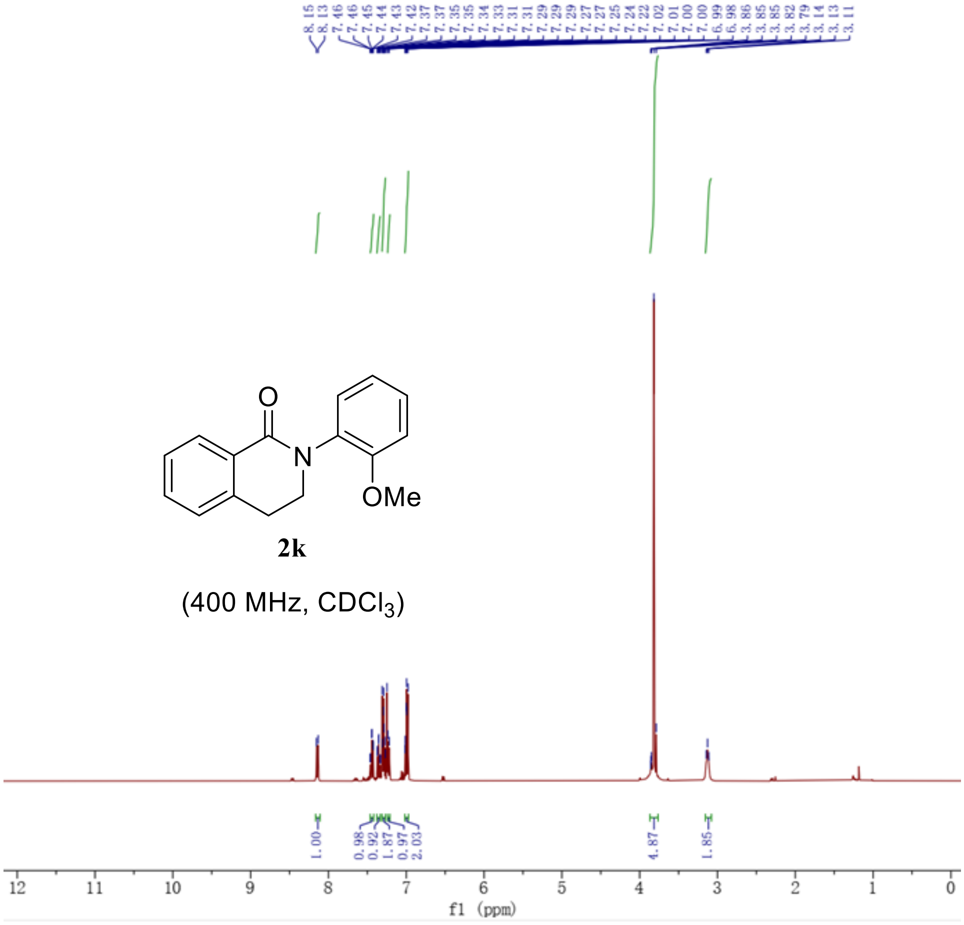

^{13}C NMR spectrum of 2k (101 MHz, $CDCl_3$)

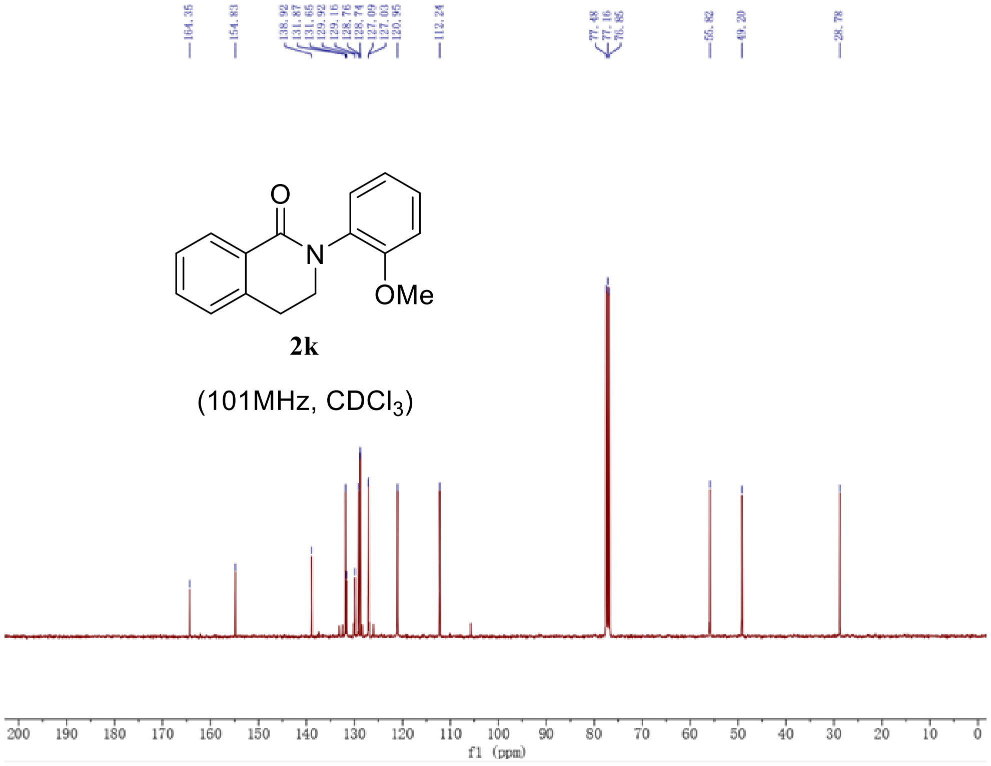

^{1}H NMR spectrum of 2l (400 MHz, CDCl$_3$)

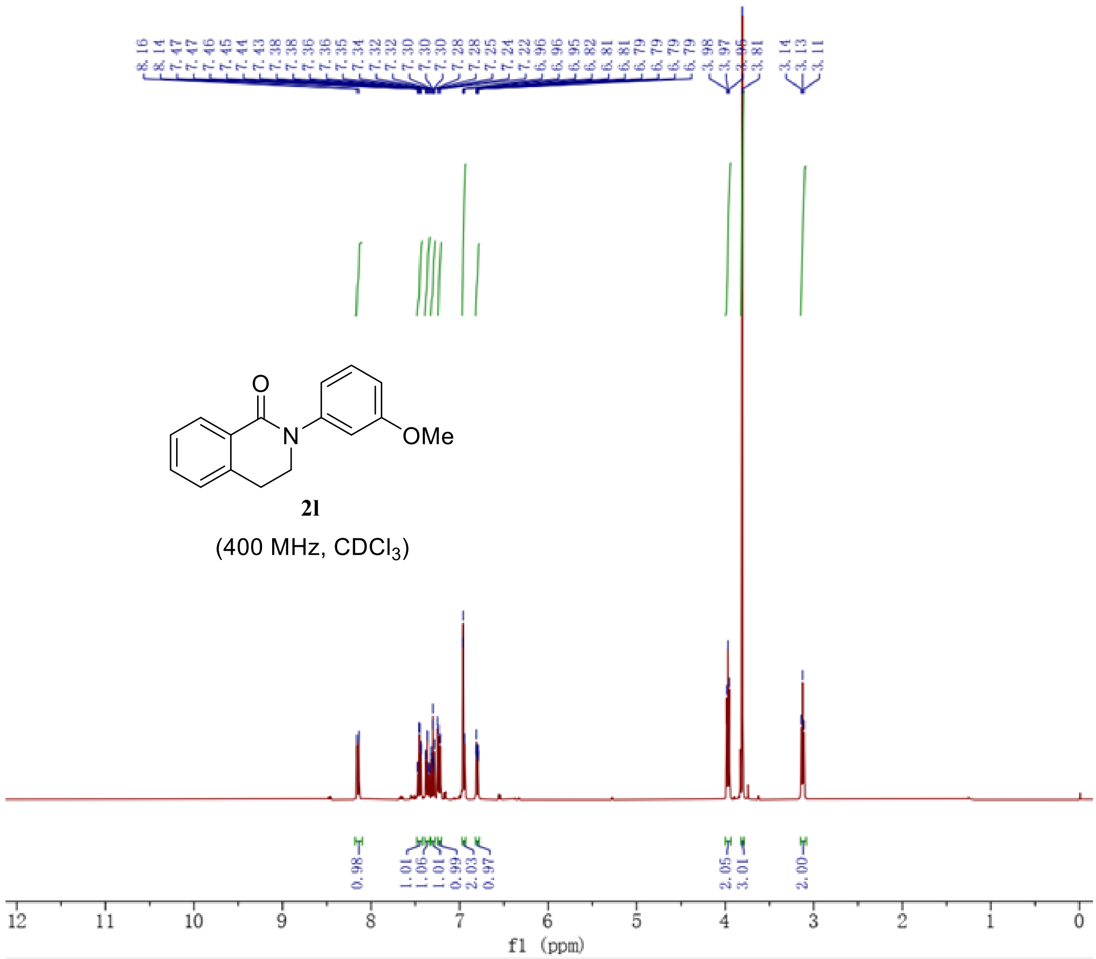

^{13}C NMR spectrum of 2l (101 MHz, CDCl$_3$)

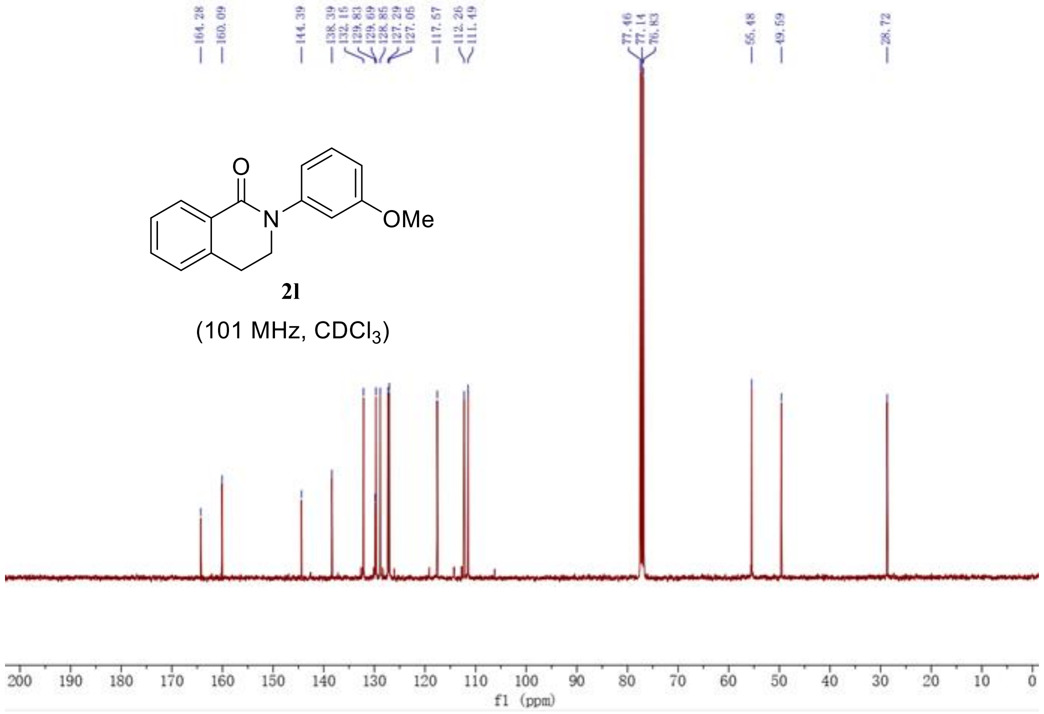

^{1}H NMR spectrum of 2m (400 MHz, $CDCl_3$)

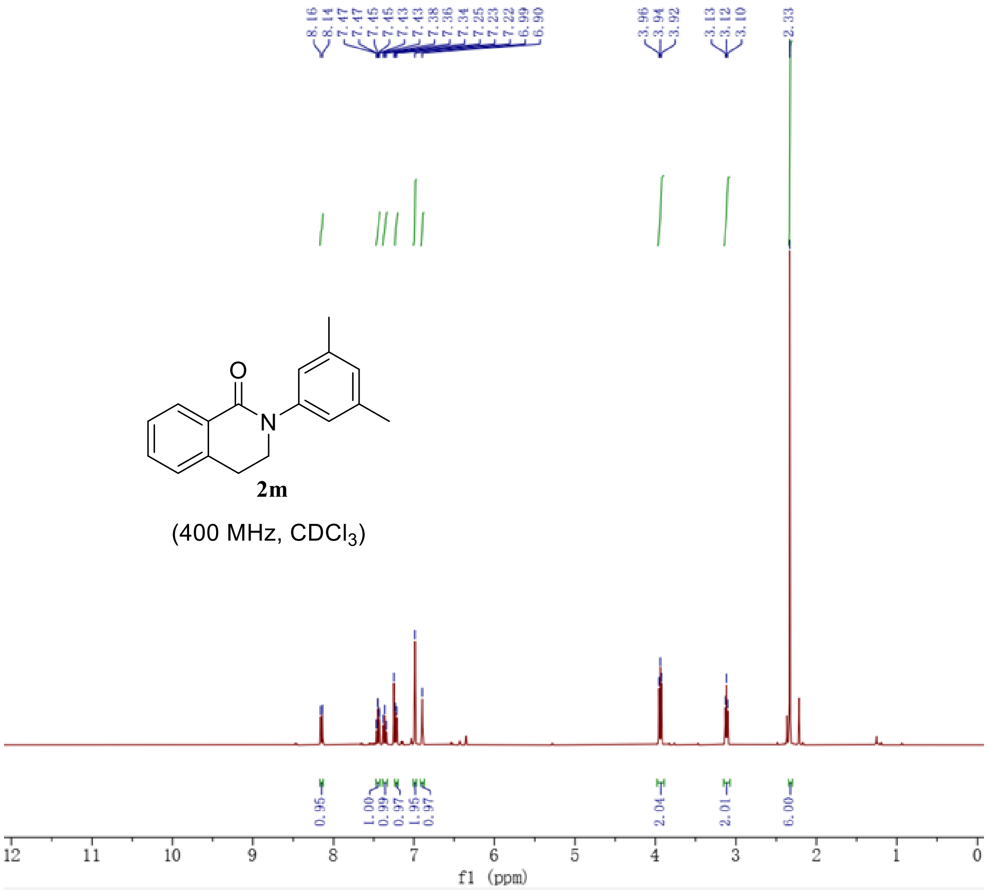

^{13}C NMR spectrum of 2m (101 MHz, $CDCl_3$)

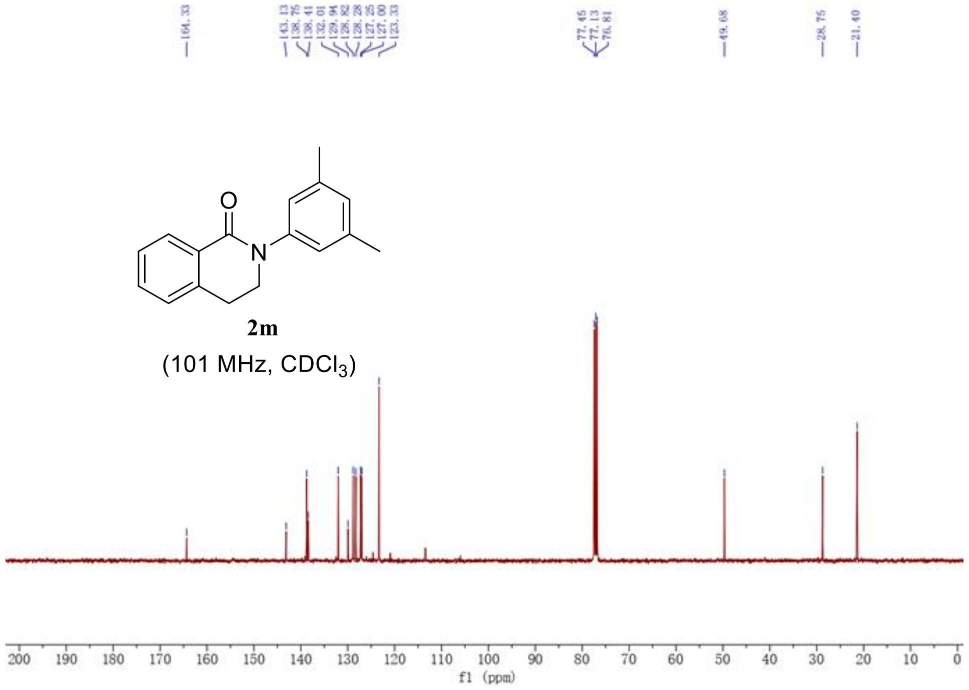

^{1}H NMR spectrum of 2n (400 MHz, $CDCl_3$)

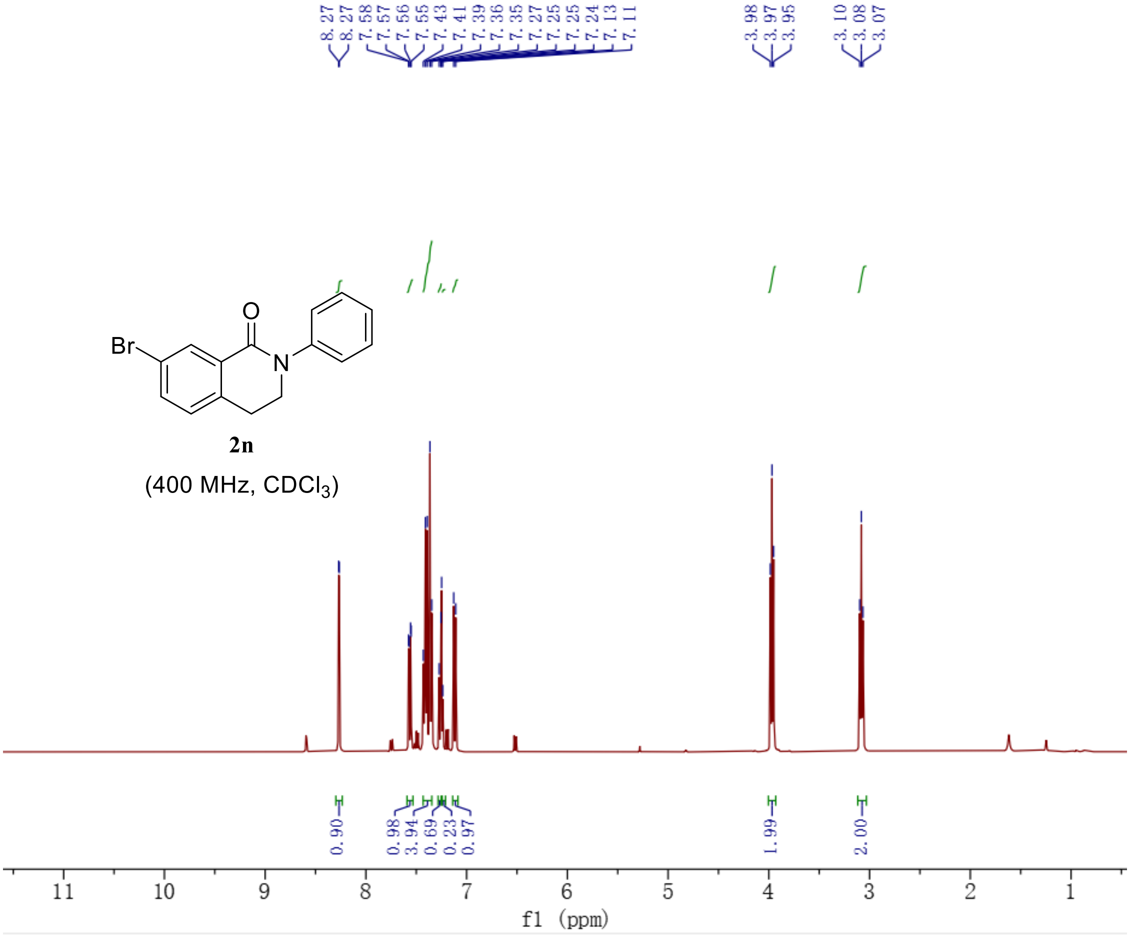

^{13}C NMR spectrum of 2n (101 MHz, $CDCl_3$)

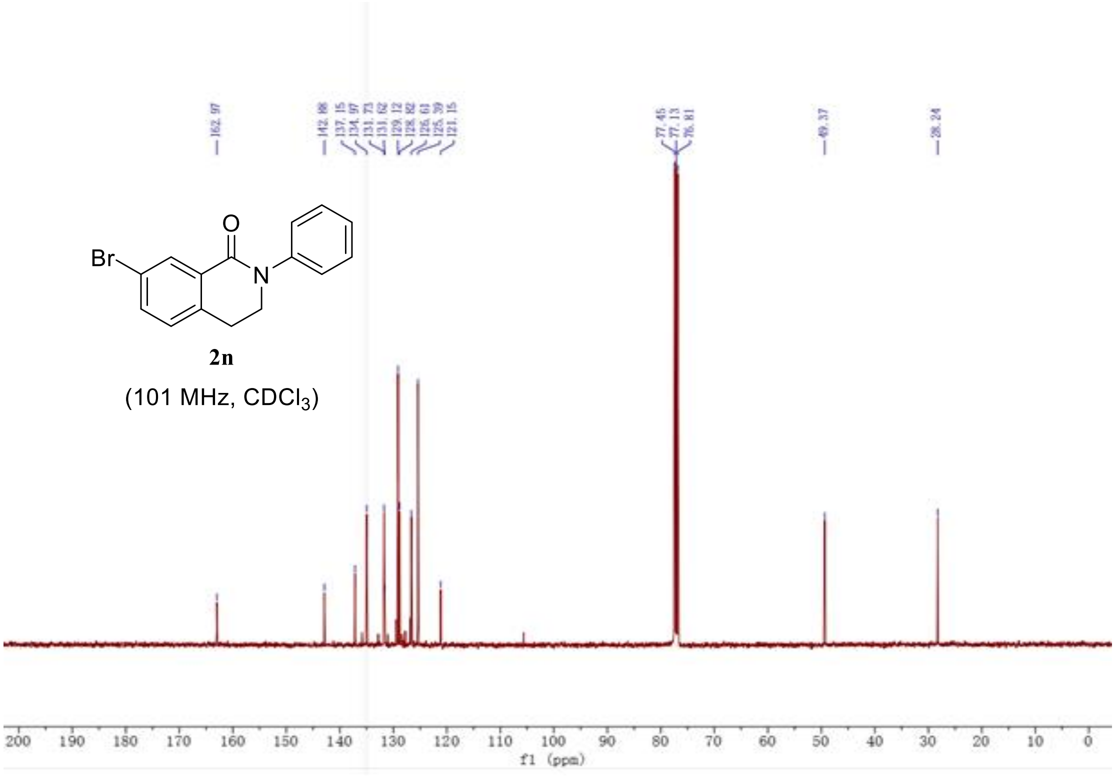

^{1}H NMR spectrum of 2o (400 MHz, $CDCl_3$)

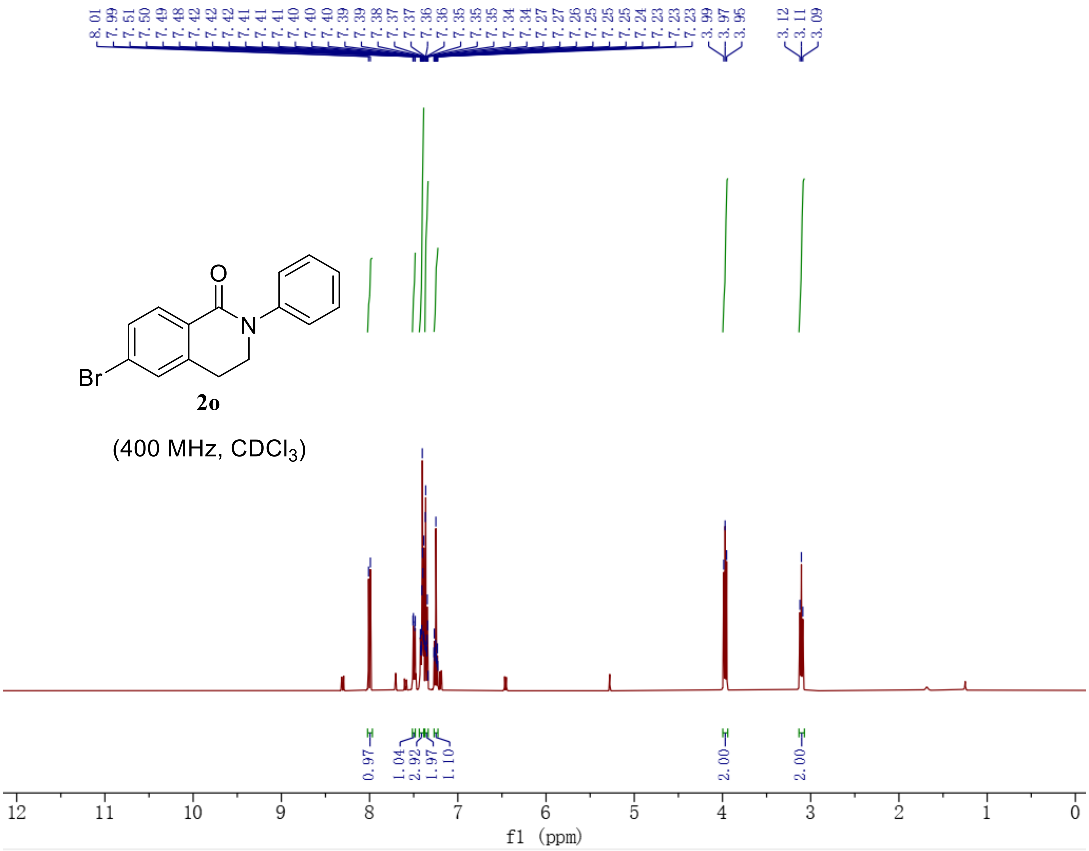

^{13}C NMR spectrum of 2o (101 MHz, $CDCl_3$)

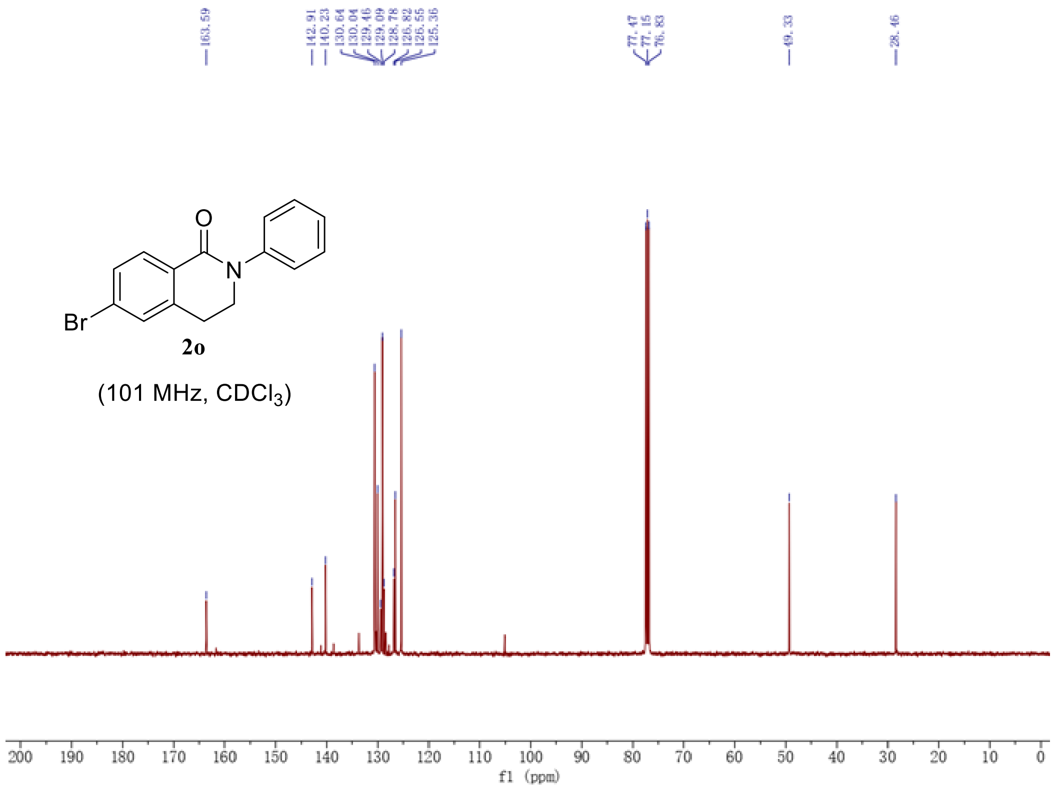

^{1}H NMR spectrum of 2p (400 MHz, $CDCl_3$)

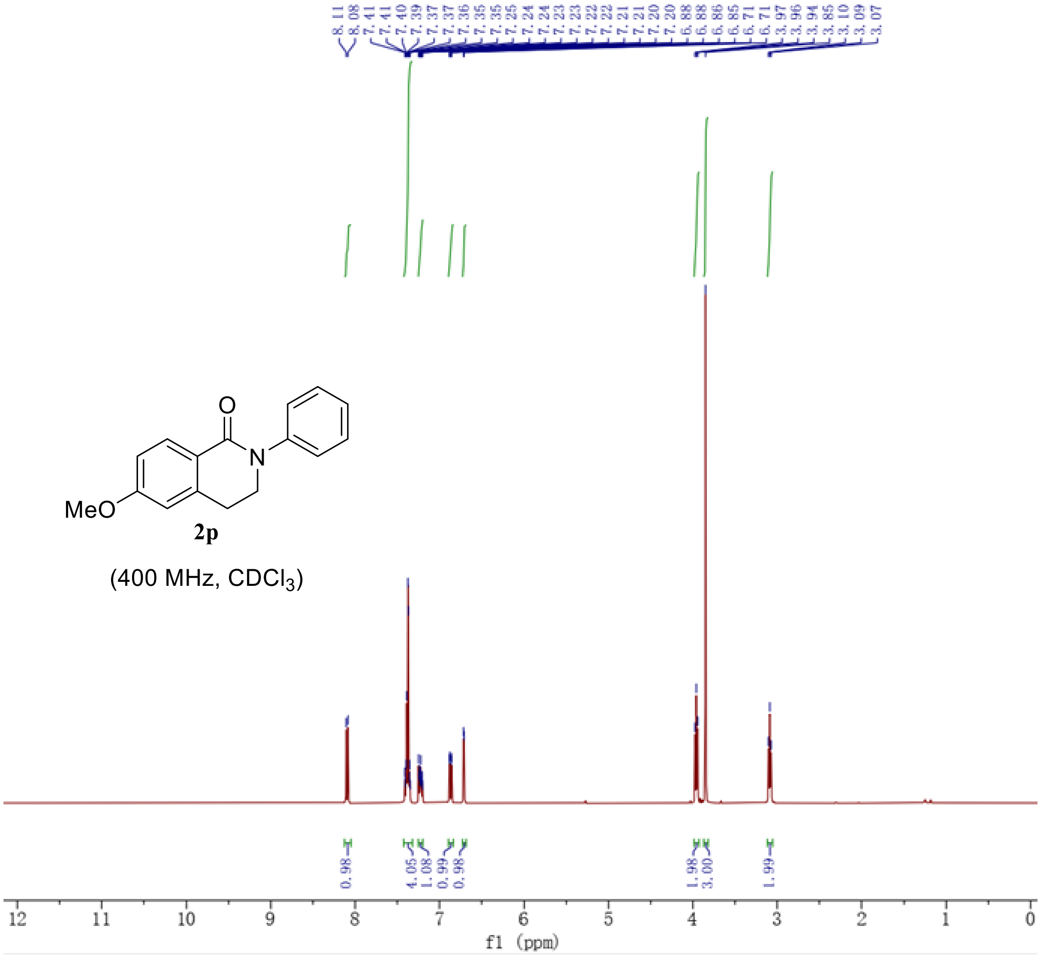

^{13}C NMR spectrum of 2p (101 MHz, $CDCl_3$)

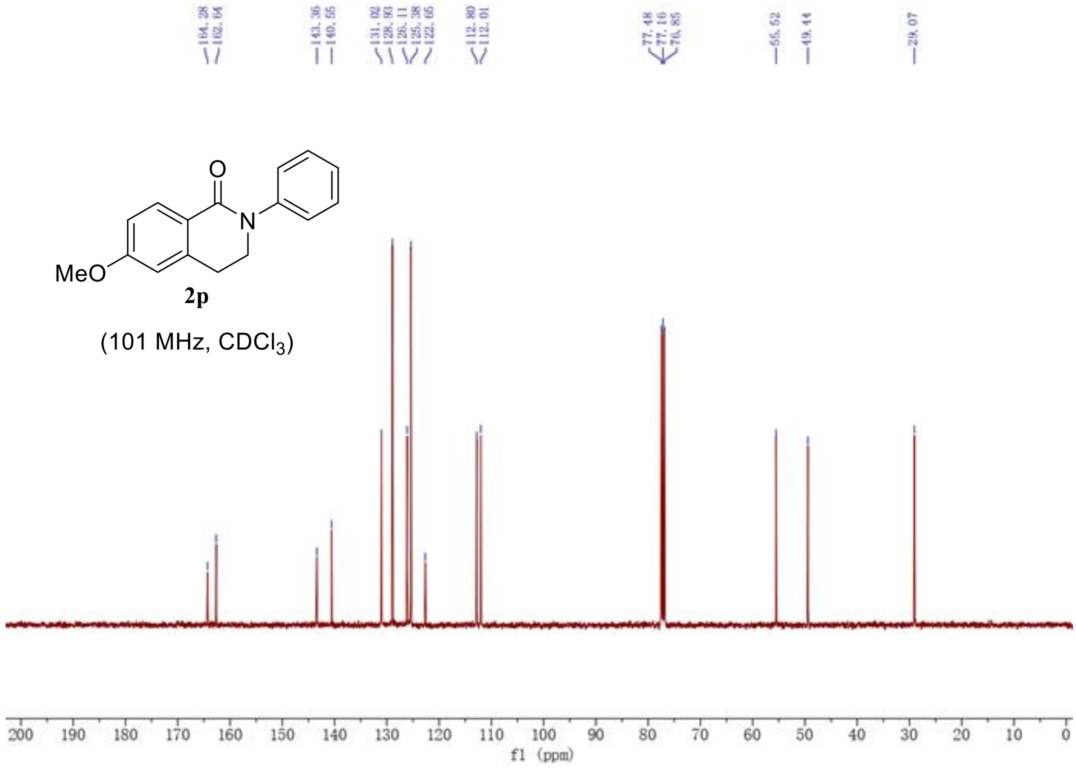

^{1}H NMR spectrum of 2q (400 MHz, $CDCl_3$)

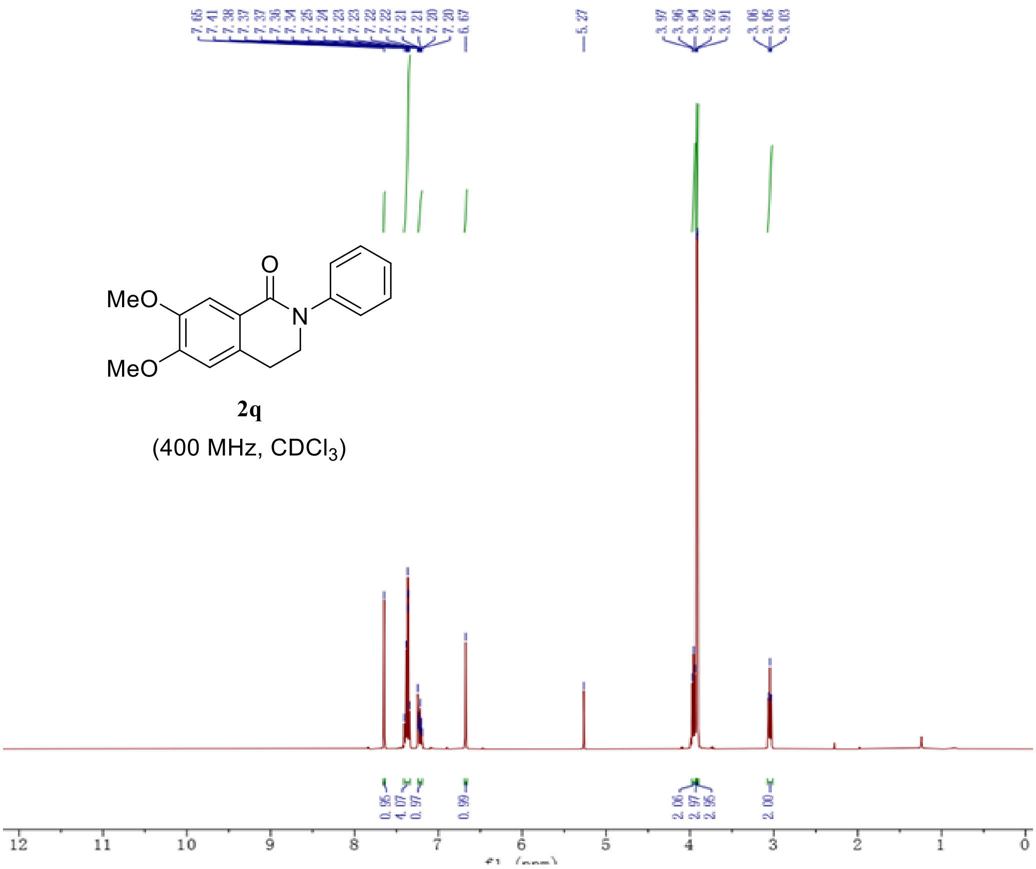

^{13}C NMR spectrum of 2q (101 MHz, $CDCl_3$)

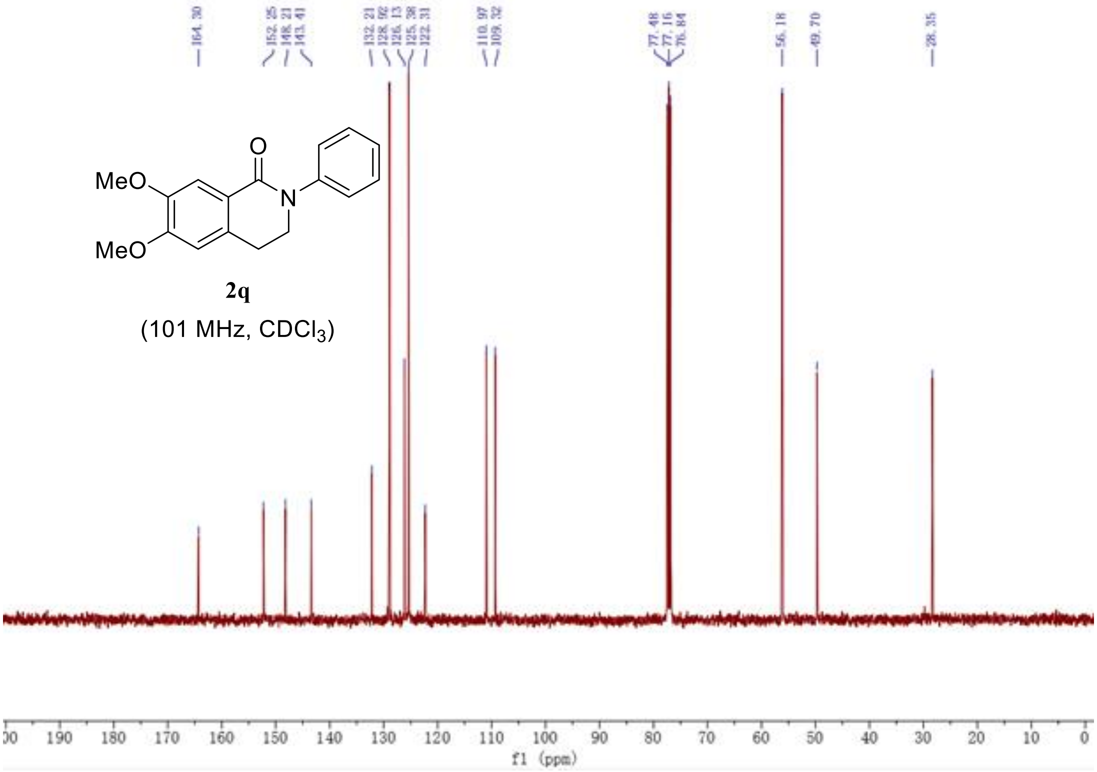

^{1}H NMR spectrum of 2r (400 MHz, $CDCl_3$)

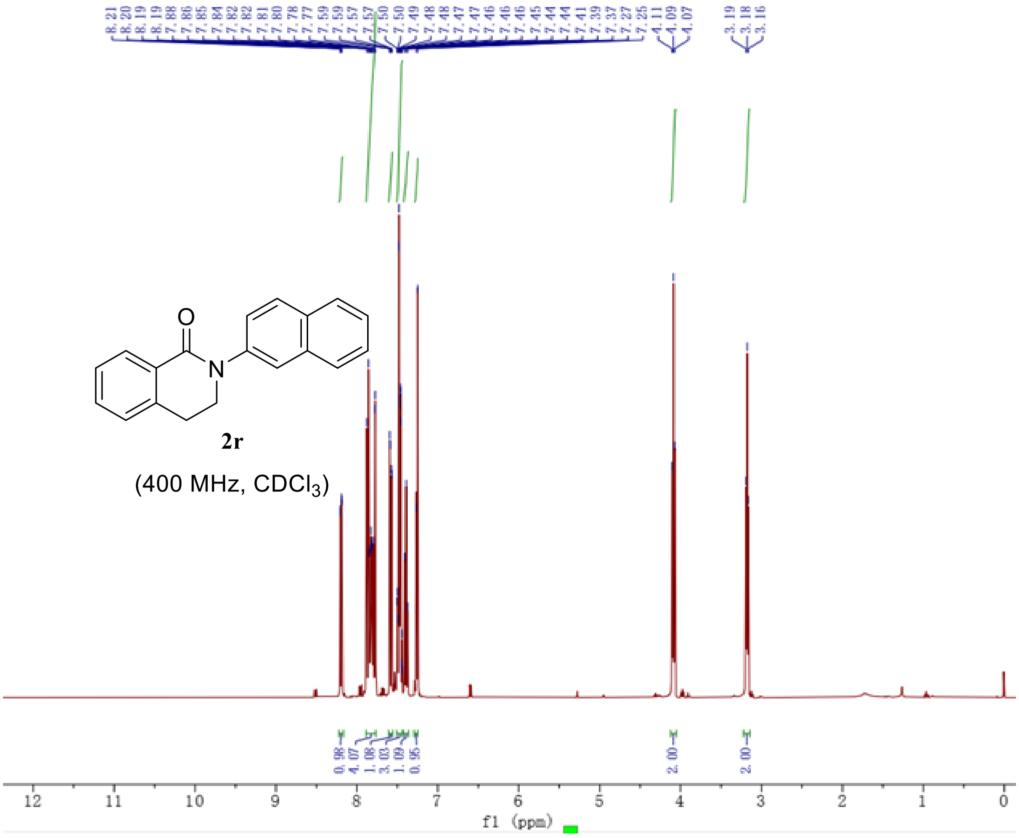

^{13}C NMR spectrum of 2r (101 MHz, $CDCl_3$)

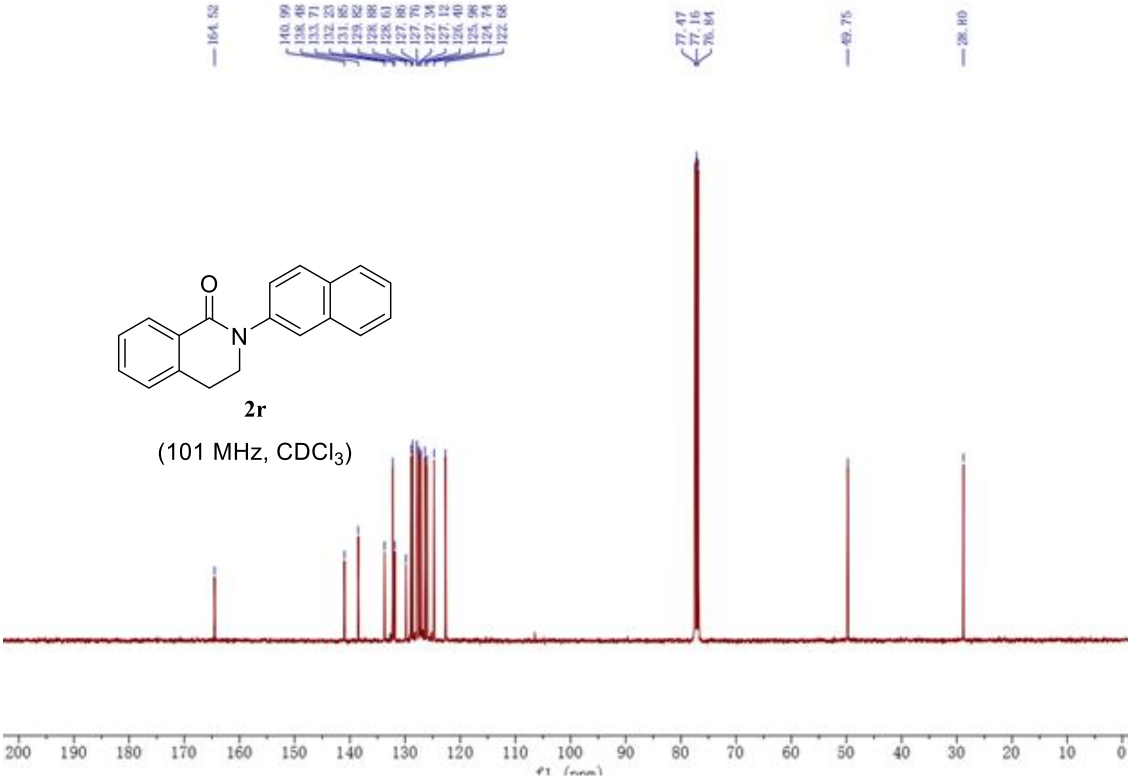

^{1}H NMR spectrum of 2s (400 MHz, CDCl$_3$)

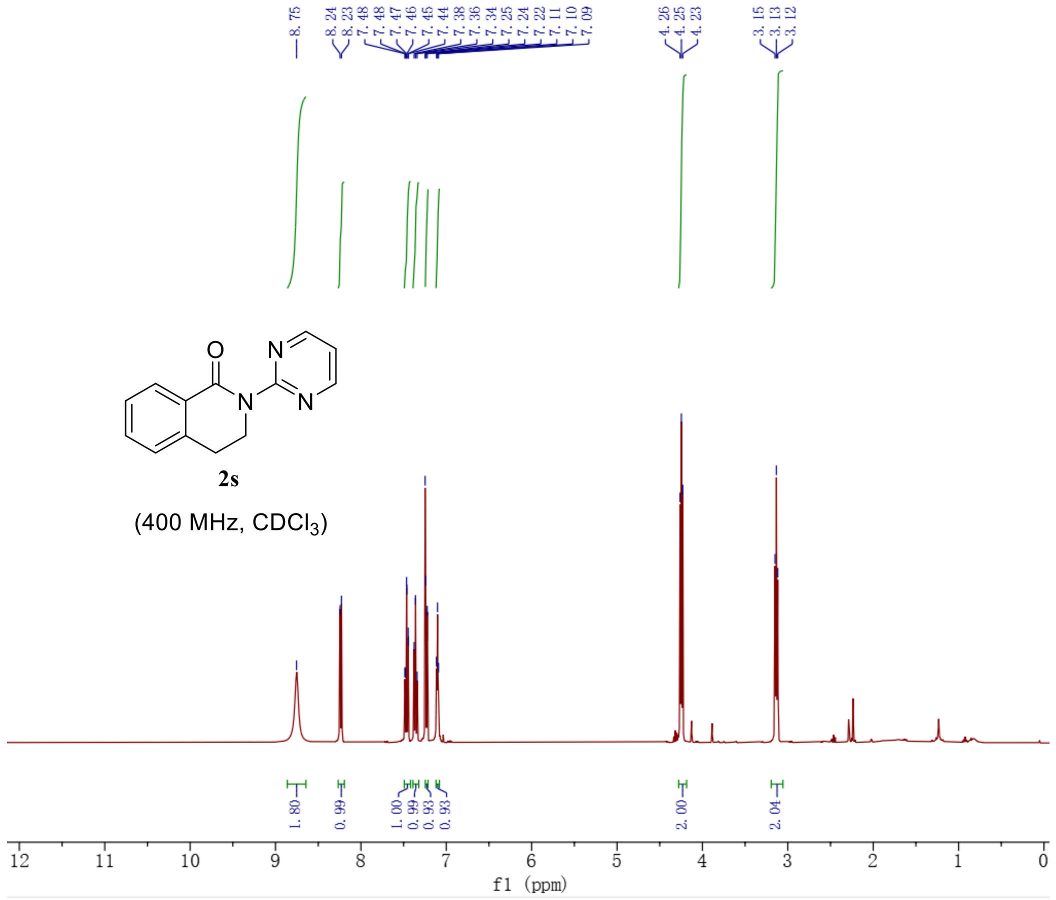

^{13}C NMR spectrum of 2s (101 MHz, CDCl$_3$)

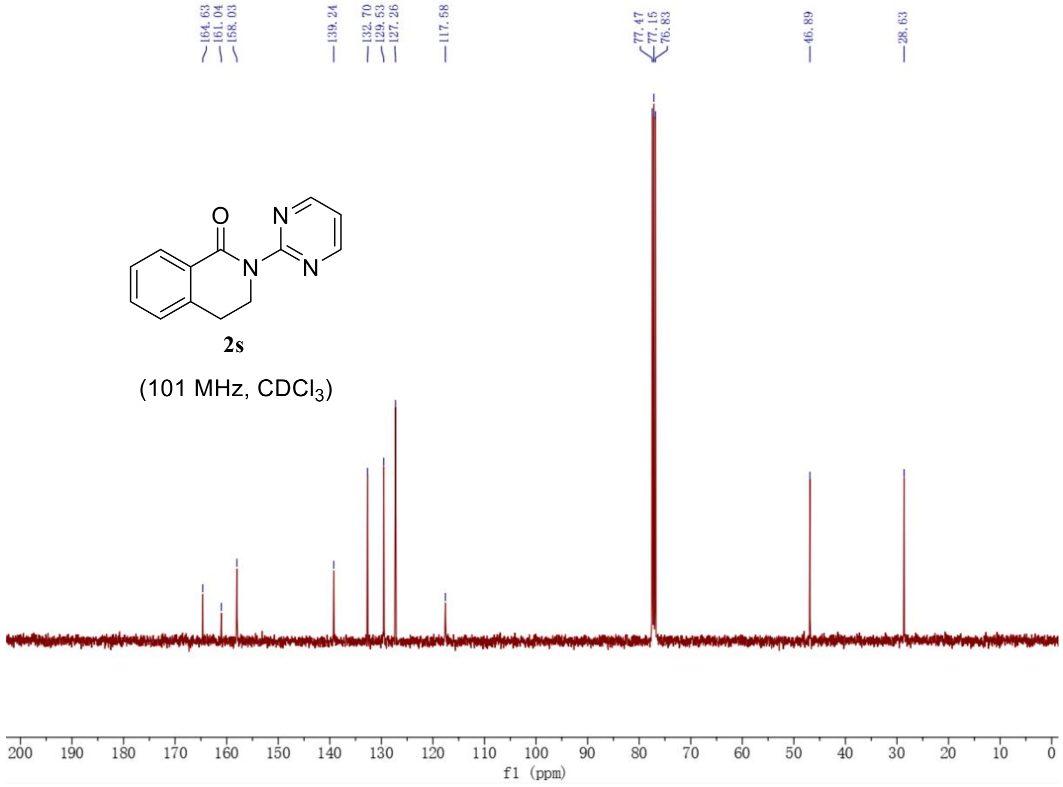

^{1}H NMR spectrum of 2t (400 MHz, $CDCl_3$)

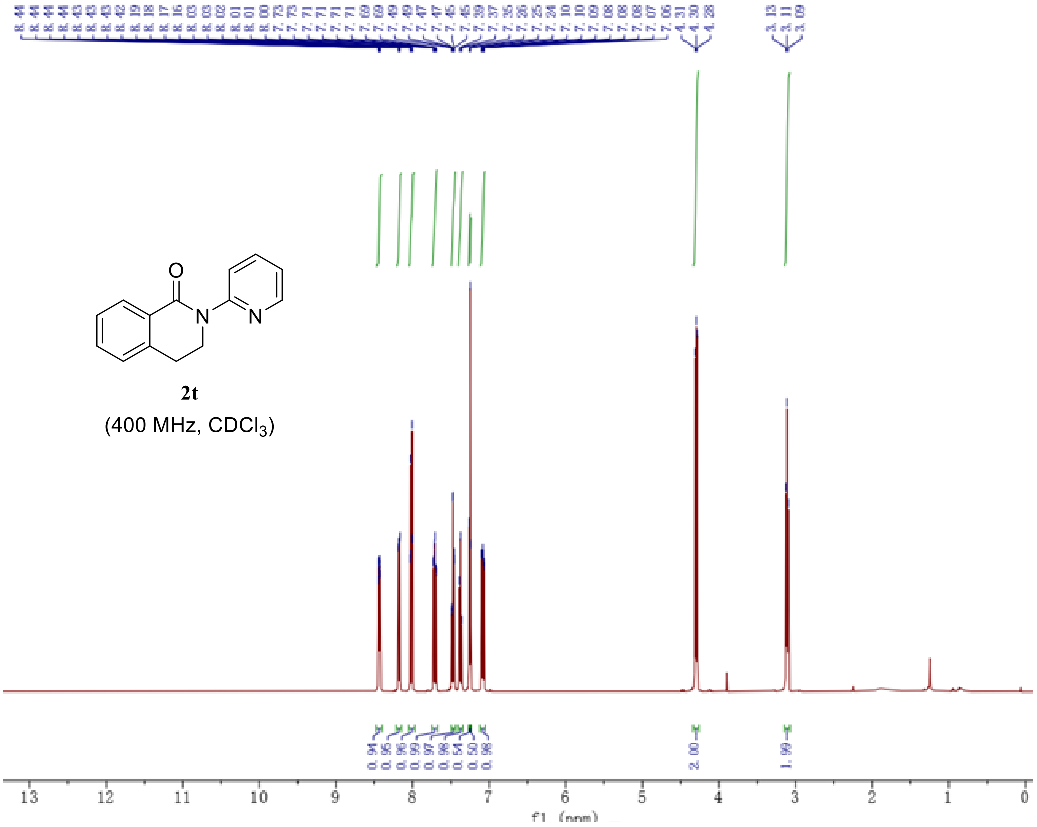

^{13}C NMR spectrum of 2t (101 MHz, $CDCl_3$)

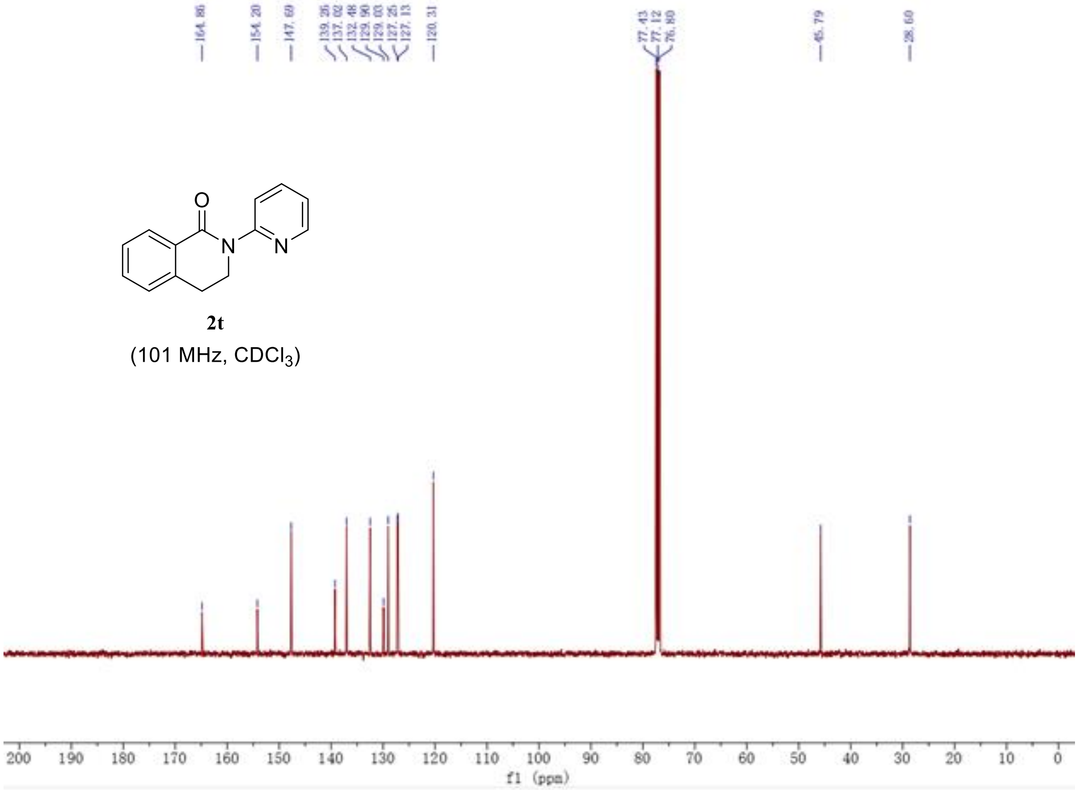

^{1}H NMR spectrum of 2u (400 MHz, $CDCl_3$)

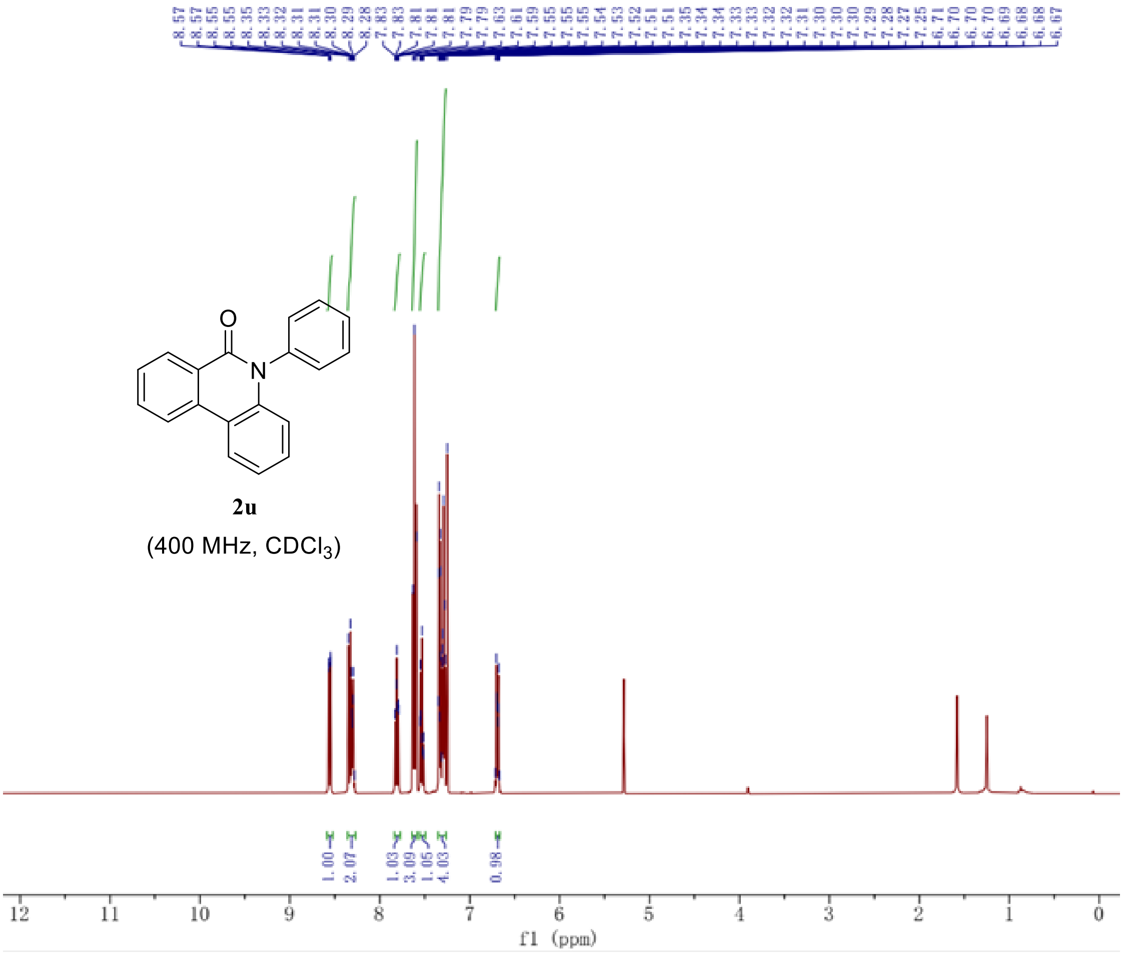

^{13}C NMR spectrum of 2u (101 MHz, $CDCl_3$)

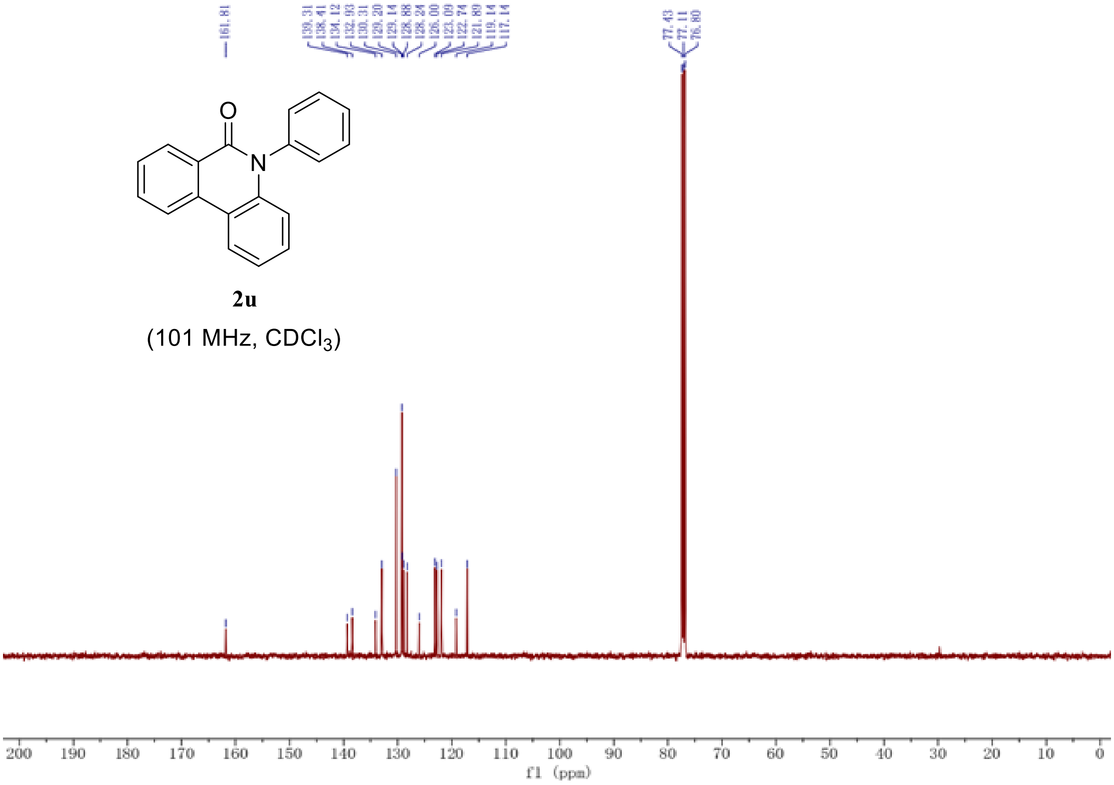

^{1}H NMR spectrum of 2v (400 MHz, $CDCl_3$)

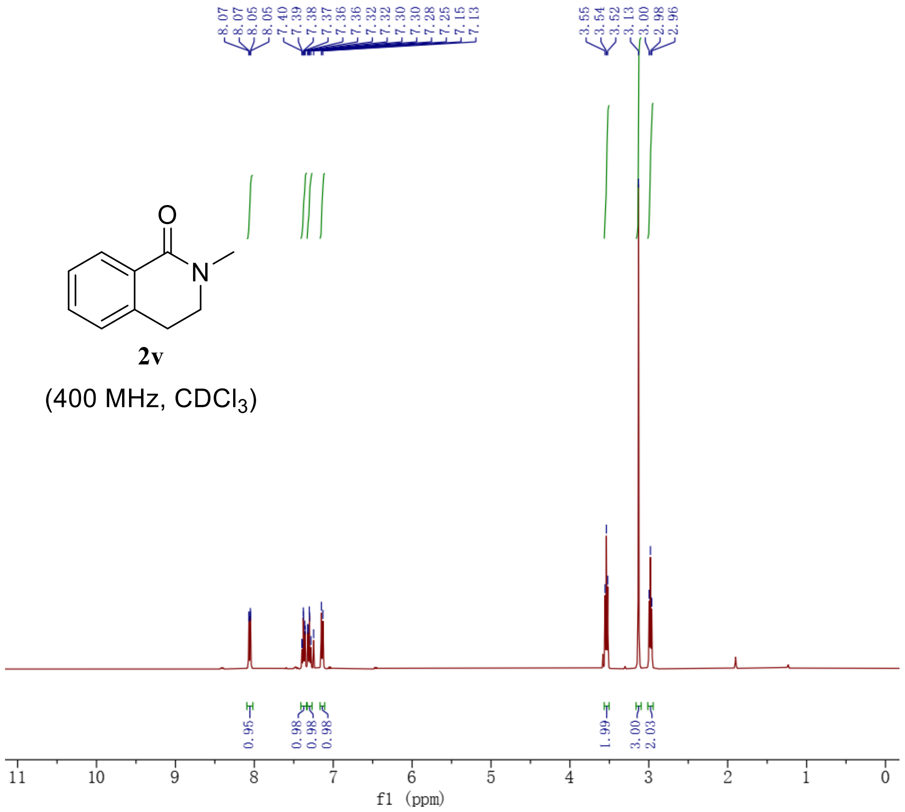

^{13}C NMR spectrum of 2v (101 MHz, $CDCl_3$)

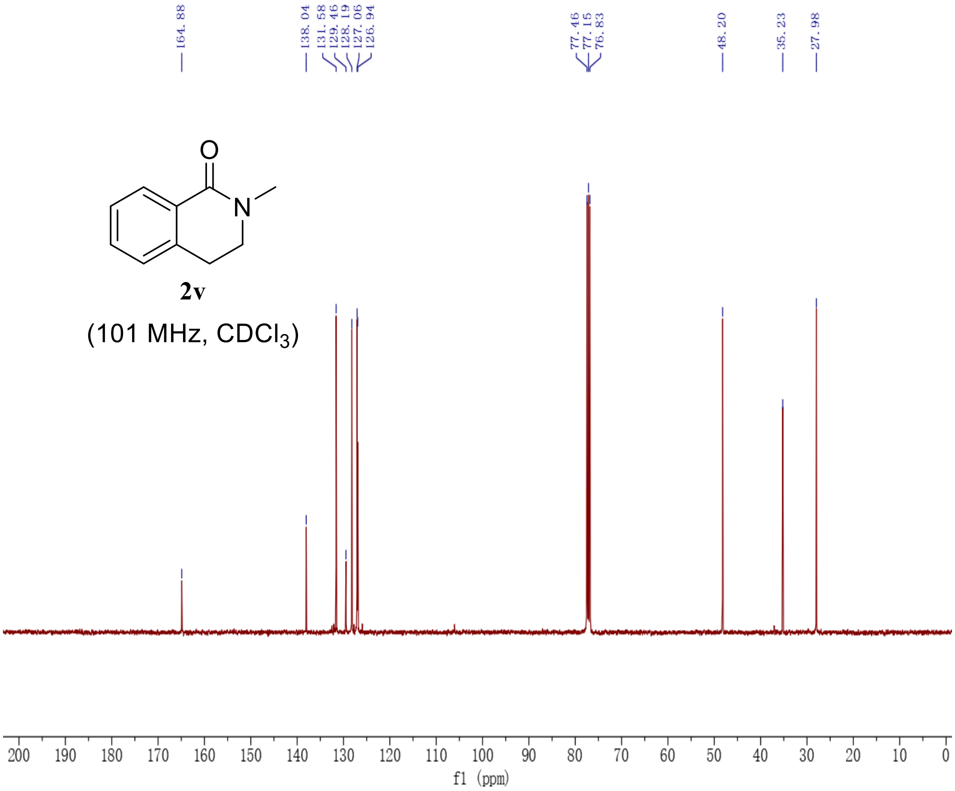

^{1}H NMR spectrum of 2w (400 MHz, $CDCl_3$)

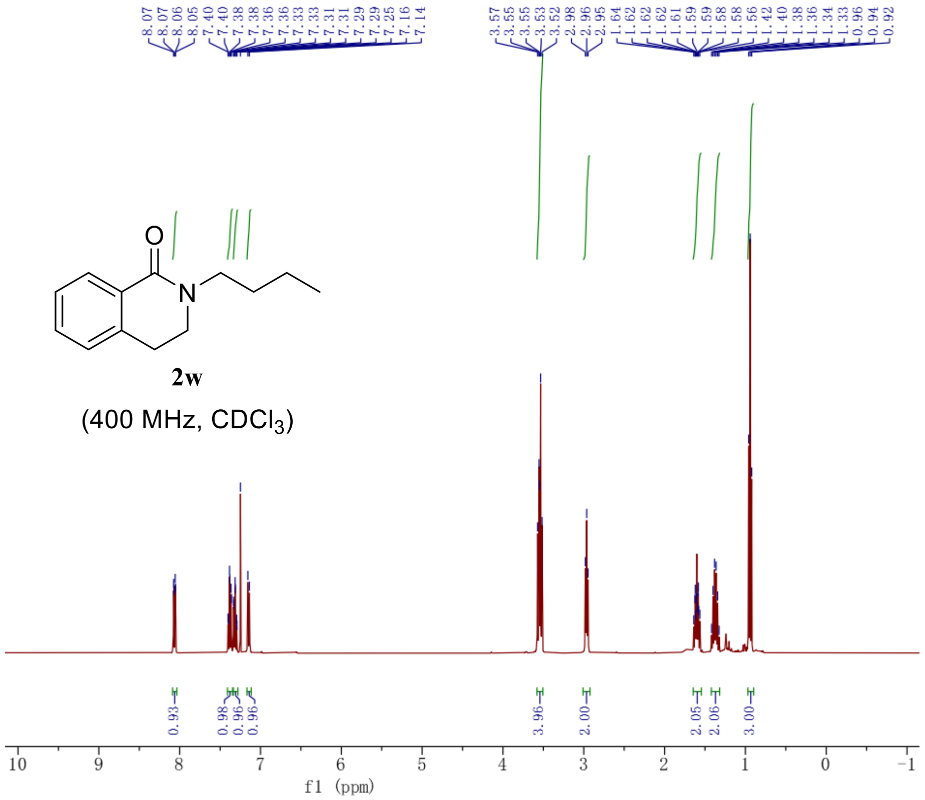

^{13}C NMR spectrum of 2w (101 MHz, $CDCl_3$)

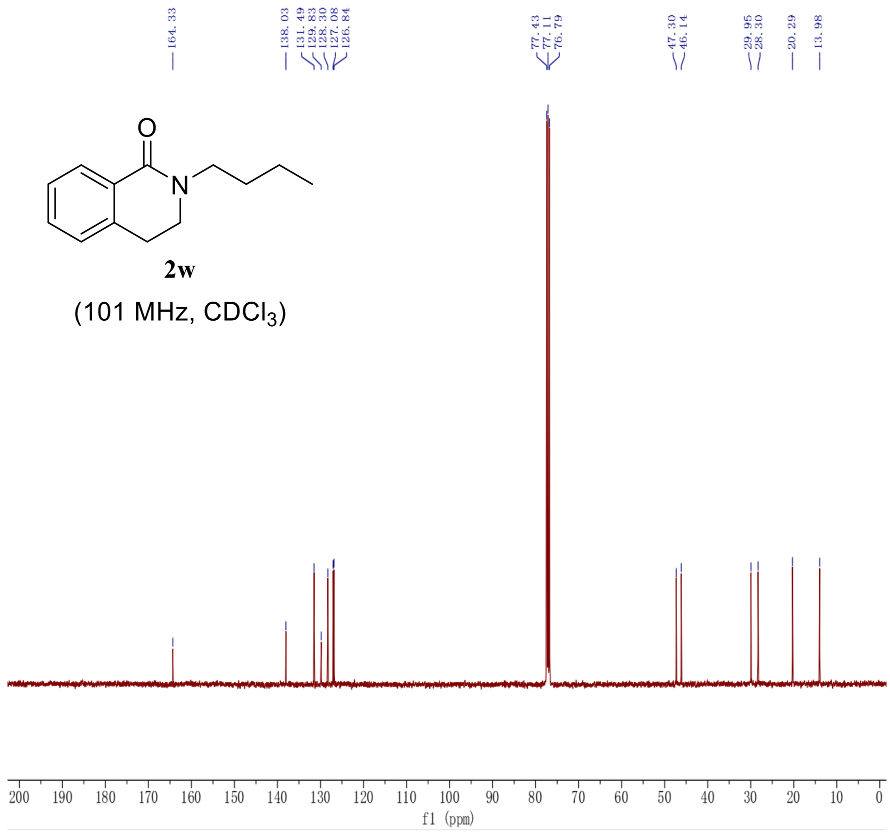

^{1}H NMR spectrum of 2x (400 MHz, $CDCl_3$)

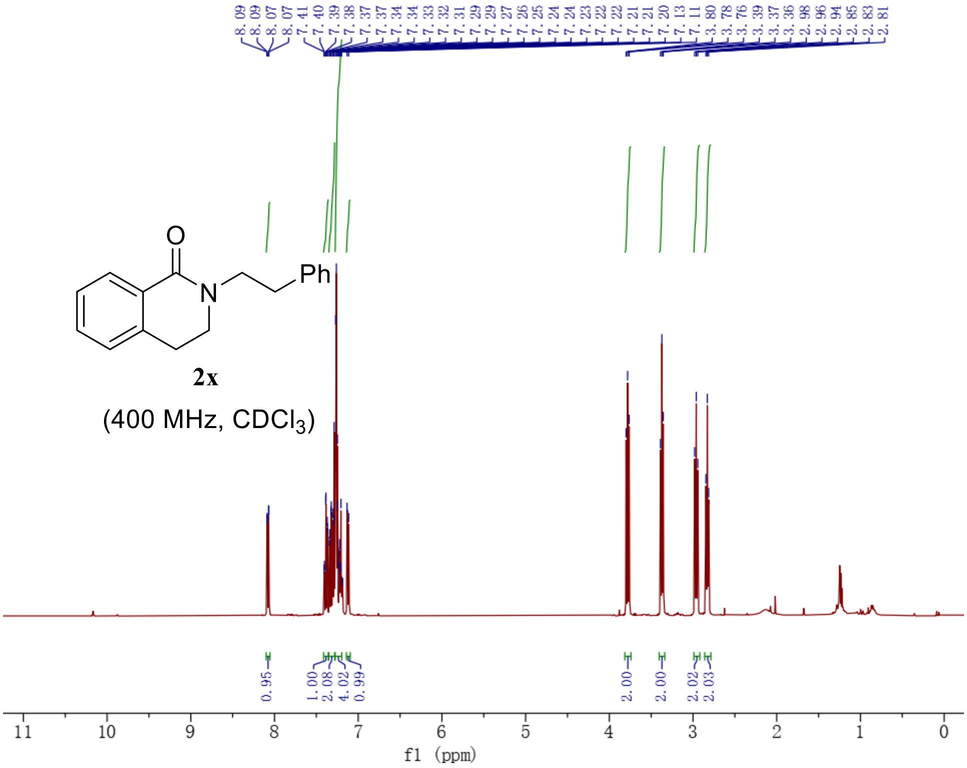

^{13}C NMR spectrum of 2x (101 MHz, $CDCl_3$)

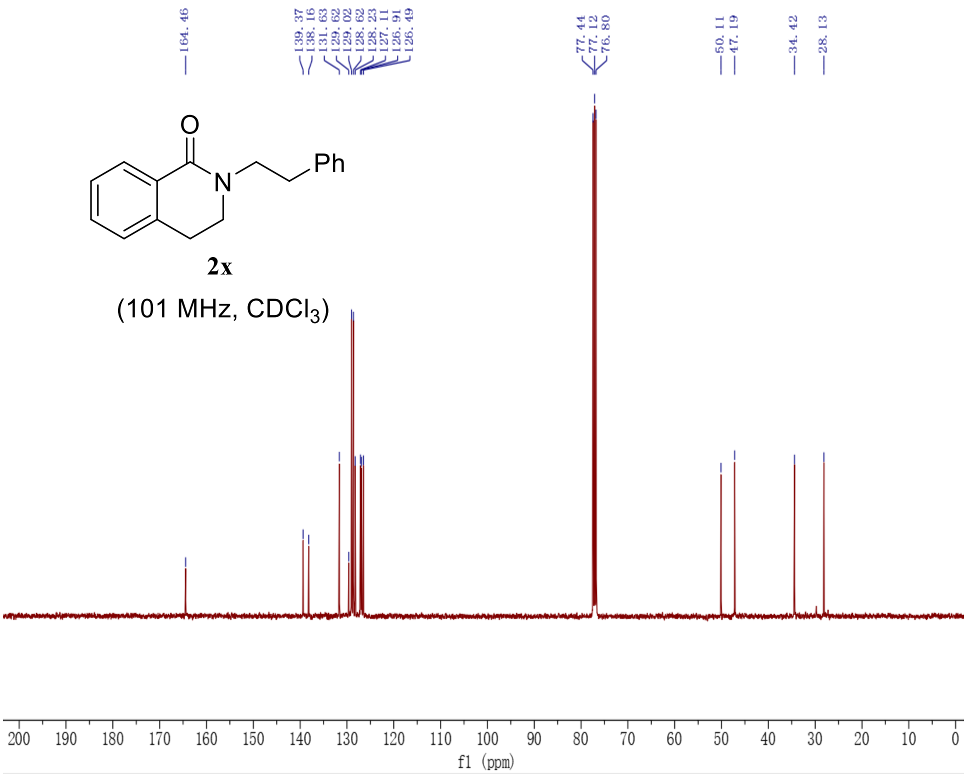

^{1}H NMR spectrum of 2y (400 MHz, $CDCl_3$)

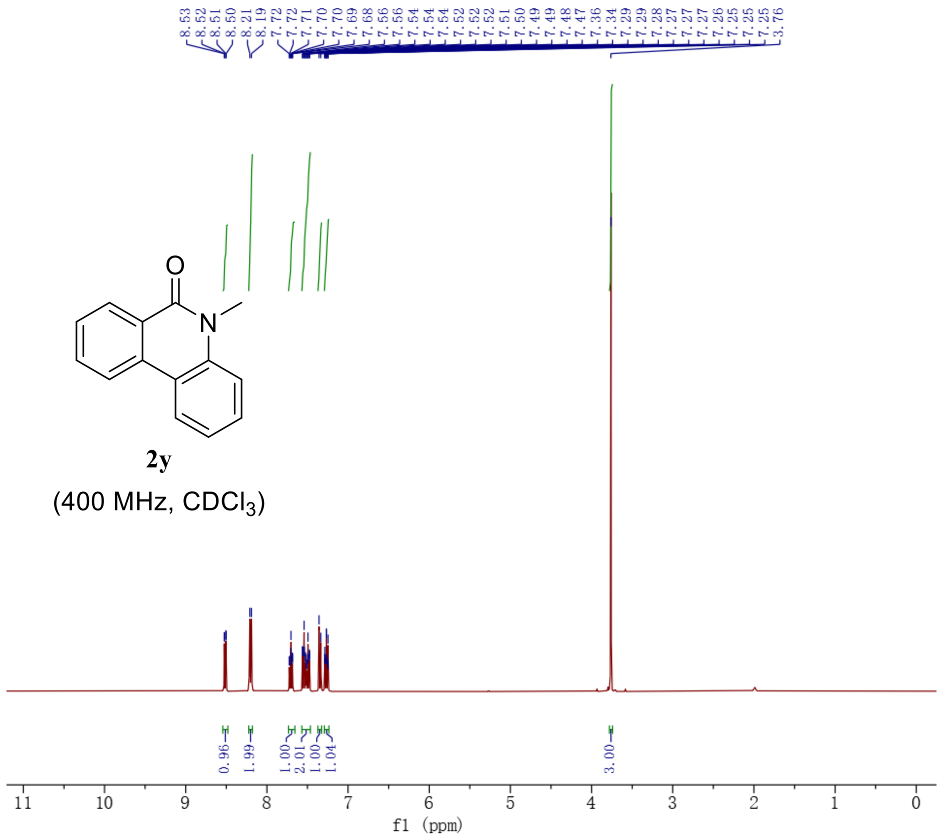

^{13}C NMR spectrum of 2y (101 MHz, $CDCl_3$)

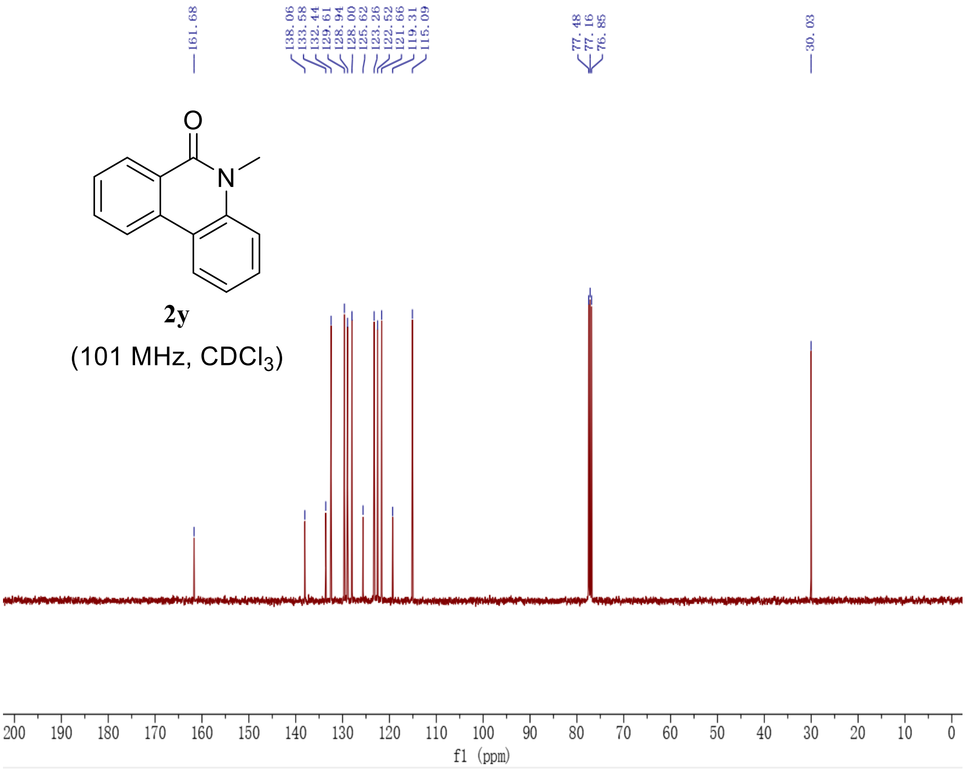

^{1}H NMR spectrum of 2z (400 MHz, $CDCl_3$)

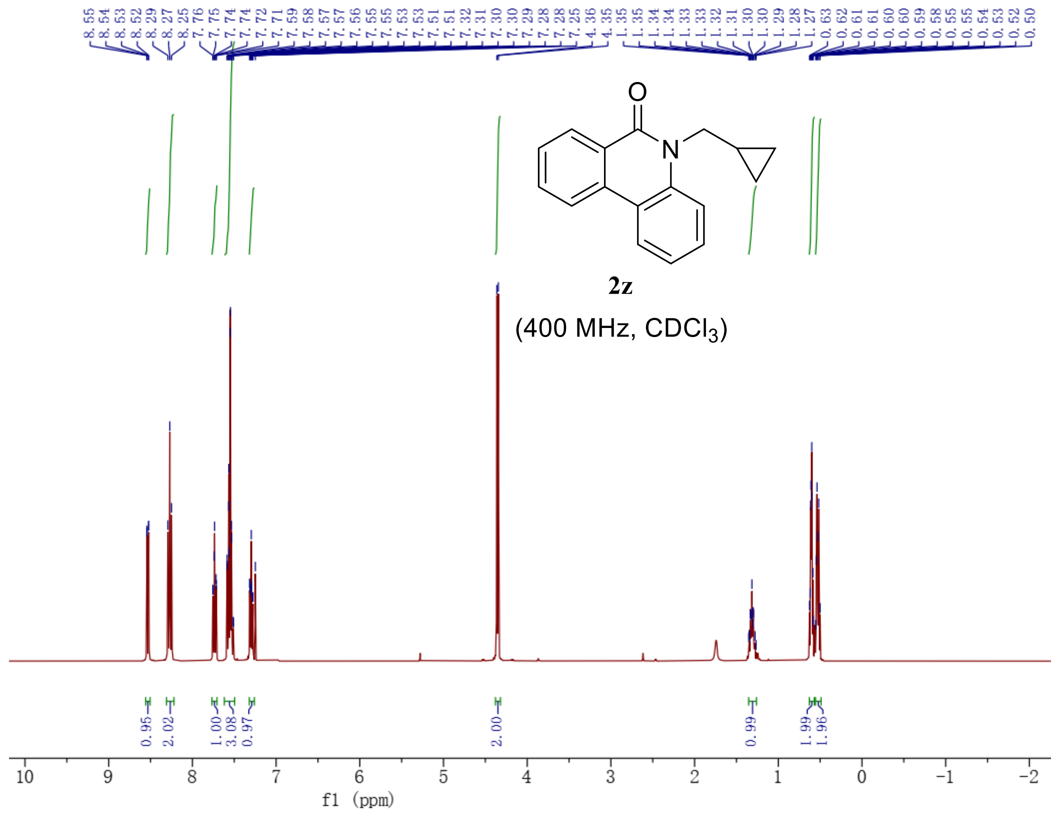

^{13}C NMR spectrum of 2z (101 MHz, $CDCl_3$)

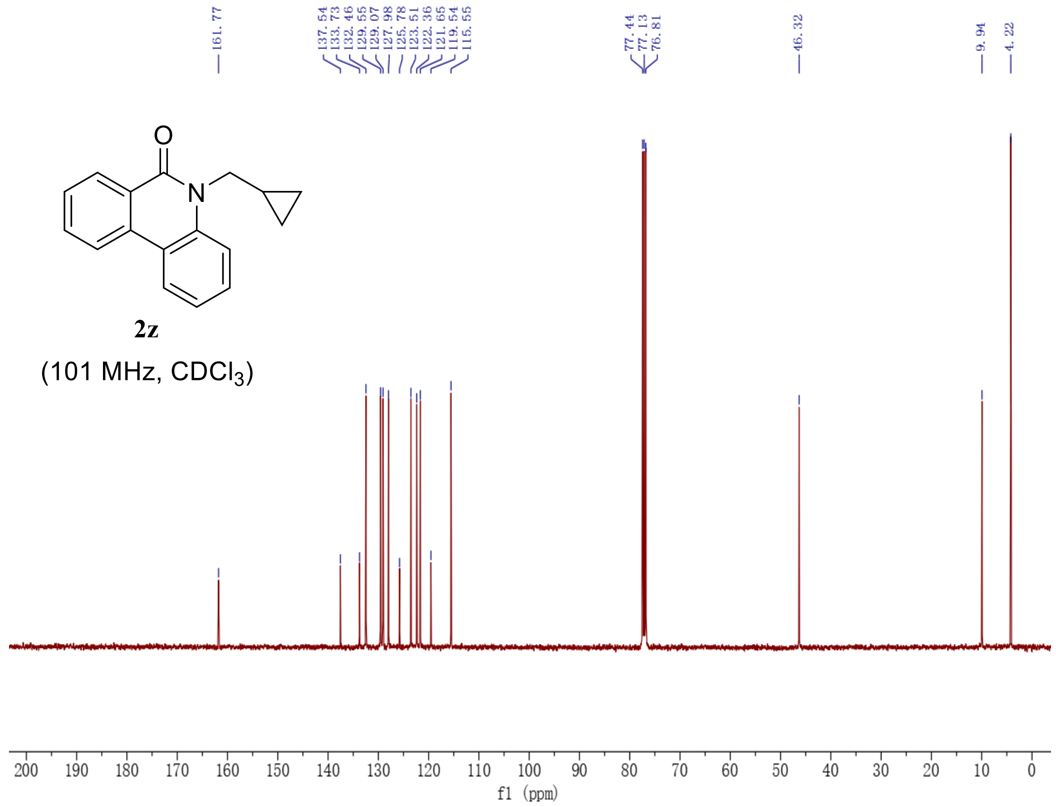

^{1}H NMR spectrum of 2a' (400 MHz, $CDCl_3$)

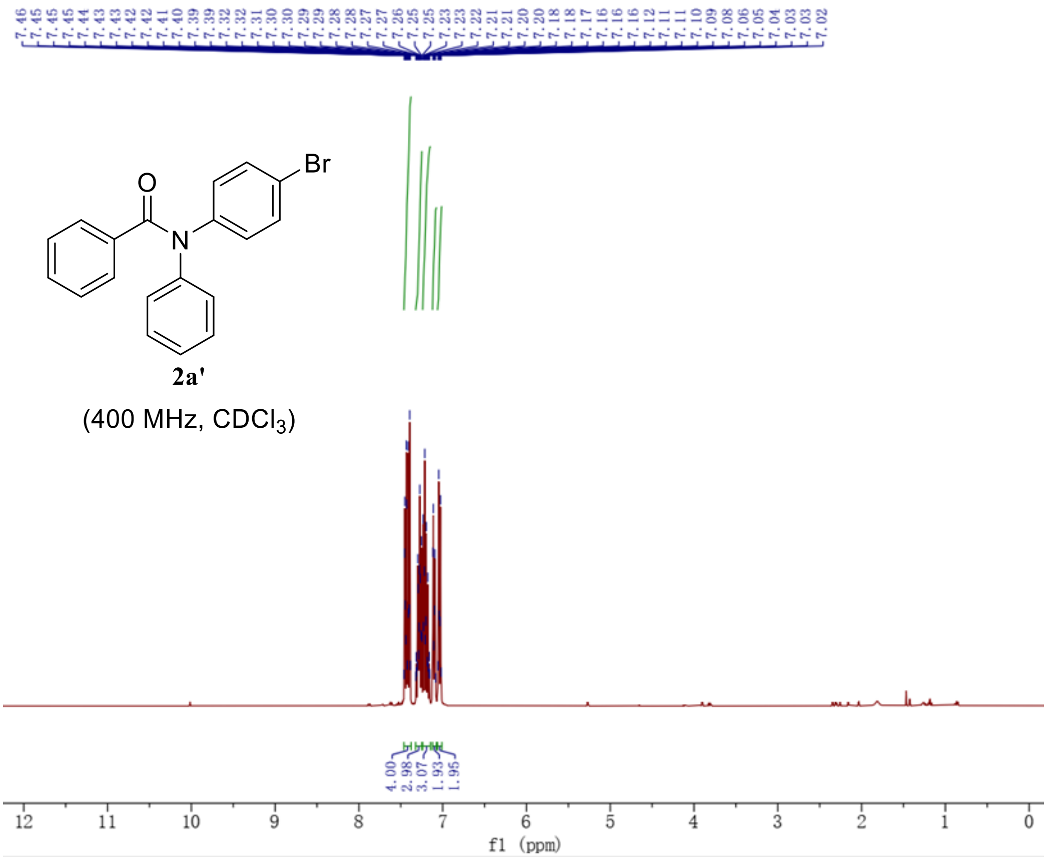

^{13}C NMR spectrum of 2a' (101 MHz, $CDCl_3$)

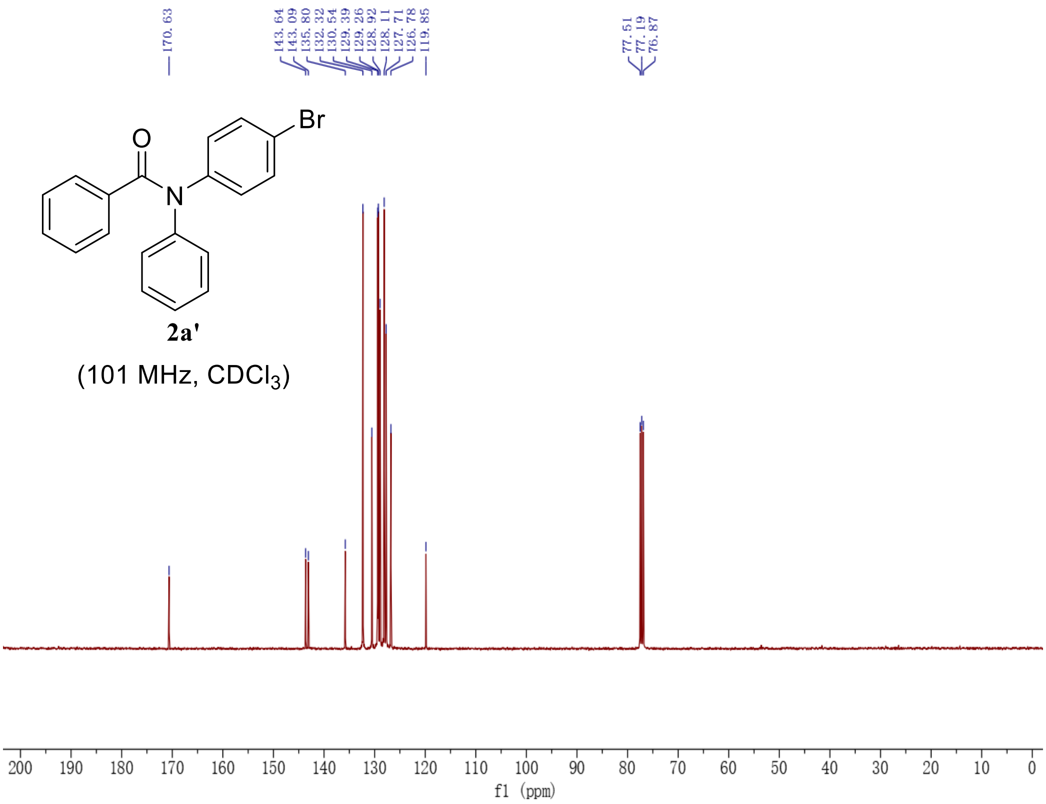

^{1}H NMR spectrum of 2b' (400 MHz, $CDCl_3$)

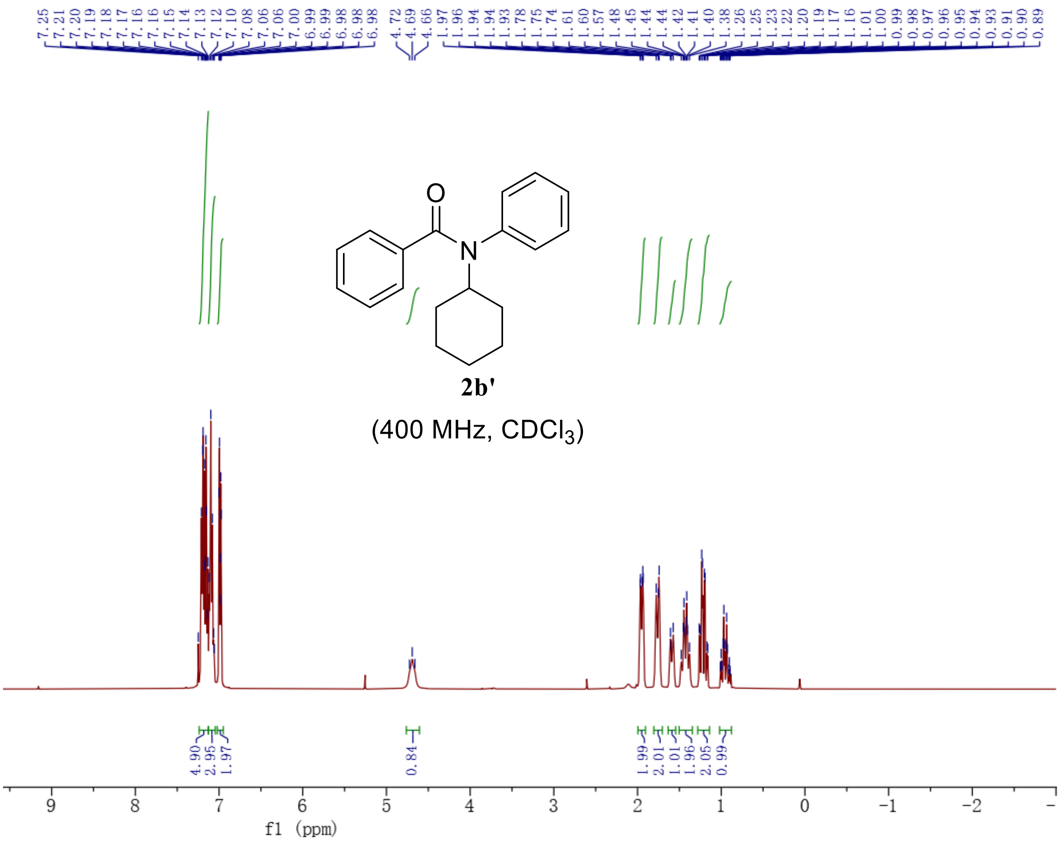

^{13}C NMR spectrum of 2b' (101 MHz, $CDCl_3$)

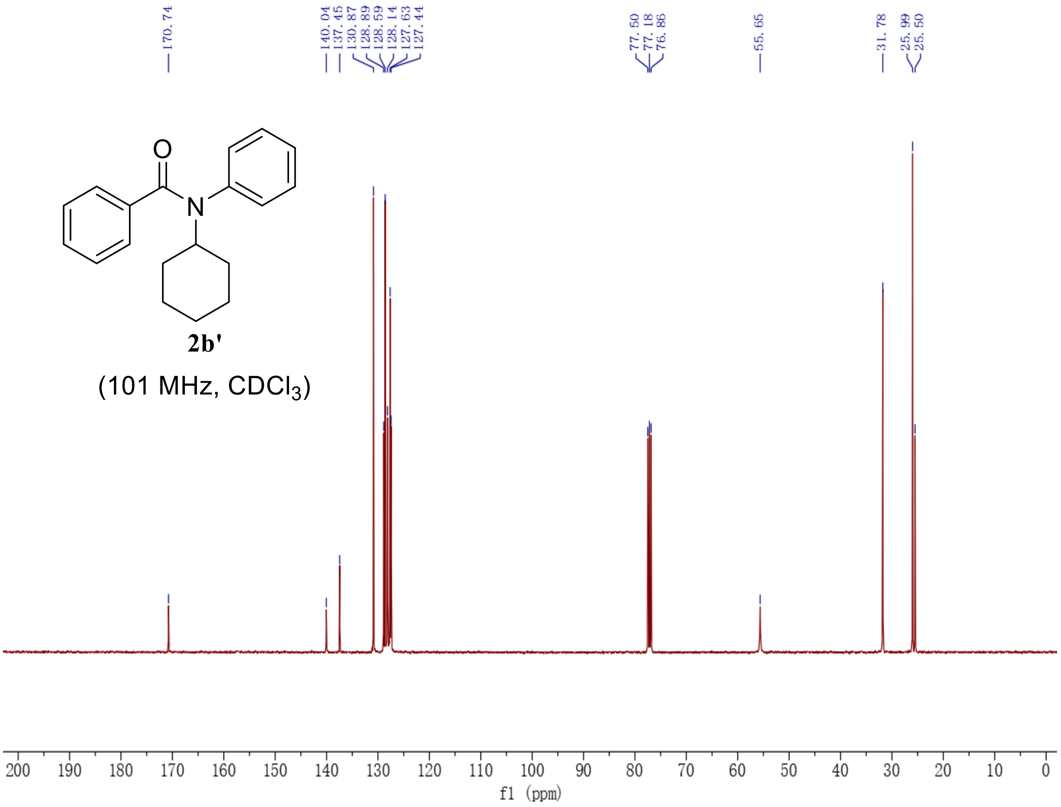

^{1}H NMR spectrum of 2c' (400 MHz, $CDCl_3$)

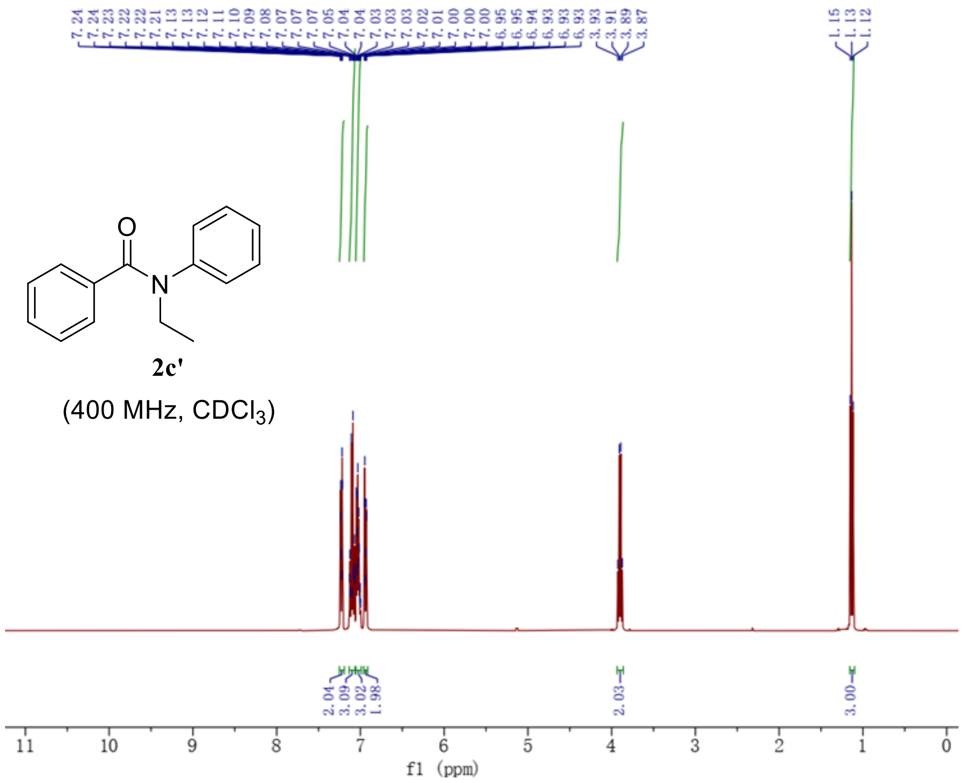

^{13}C NMR spectrum of 2c' (101 MHz, $CDCl_3$)

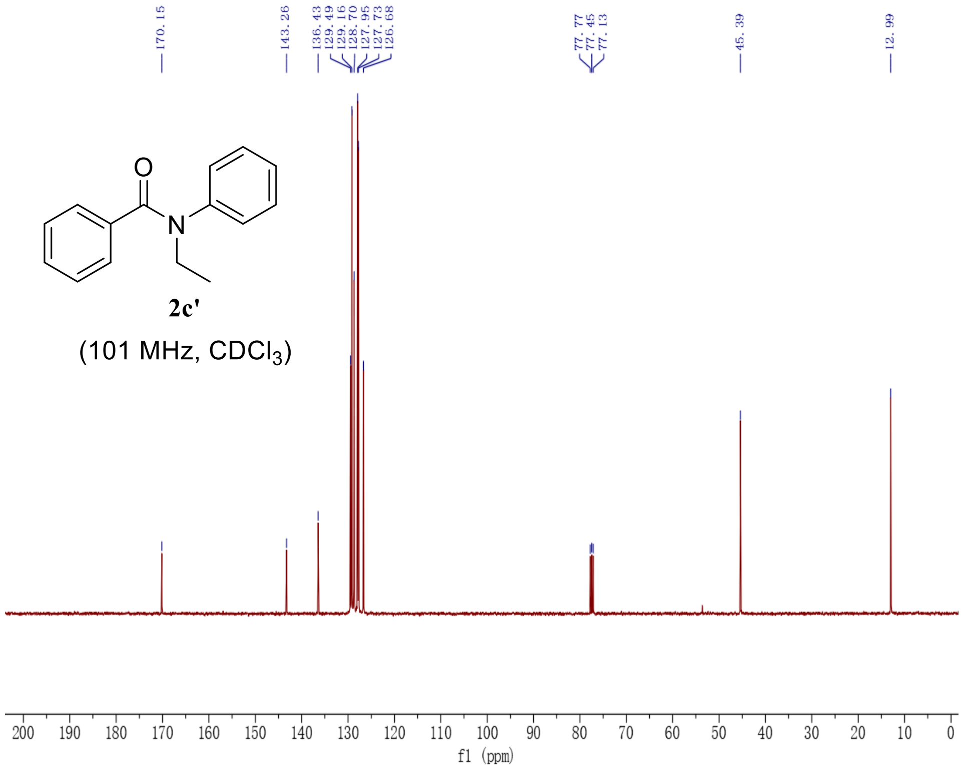

^{1}H NMR spectrum of 4a (400 MHz, $CDCl_3$)

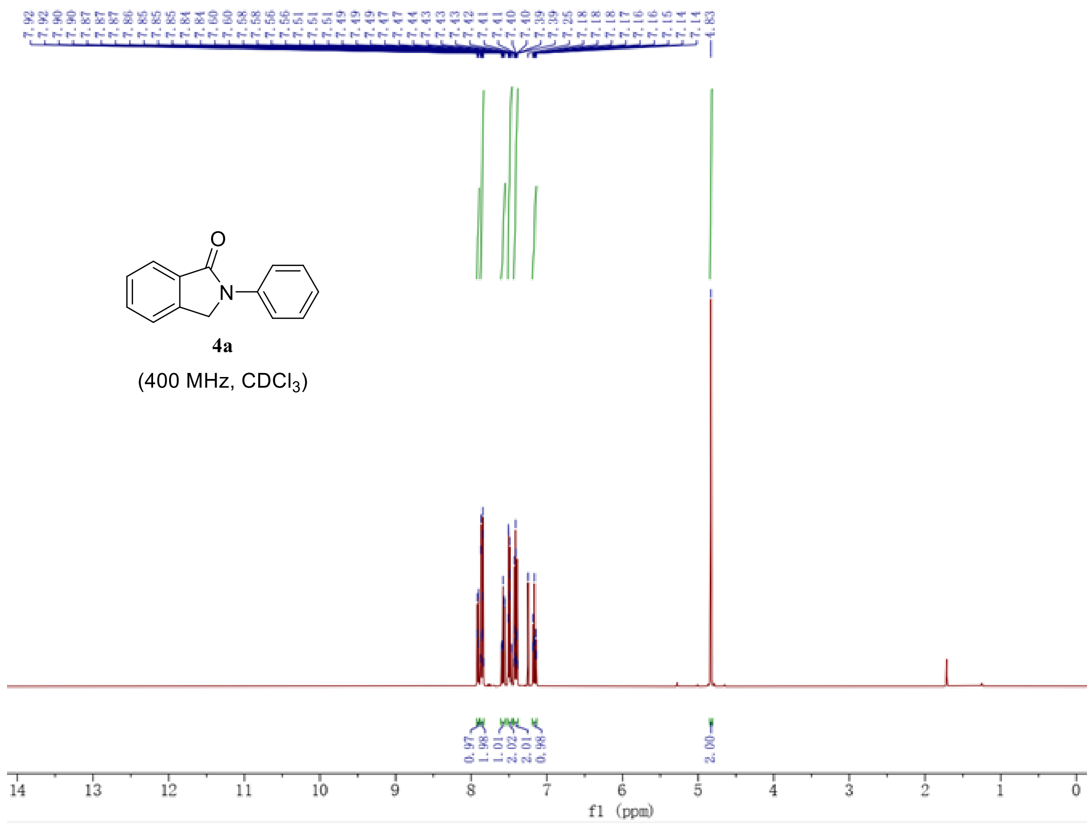

^{13}C NMR spectrum of 4a (101 MHz, $CDCl_3$)

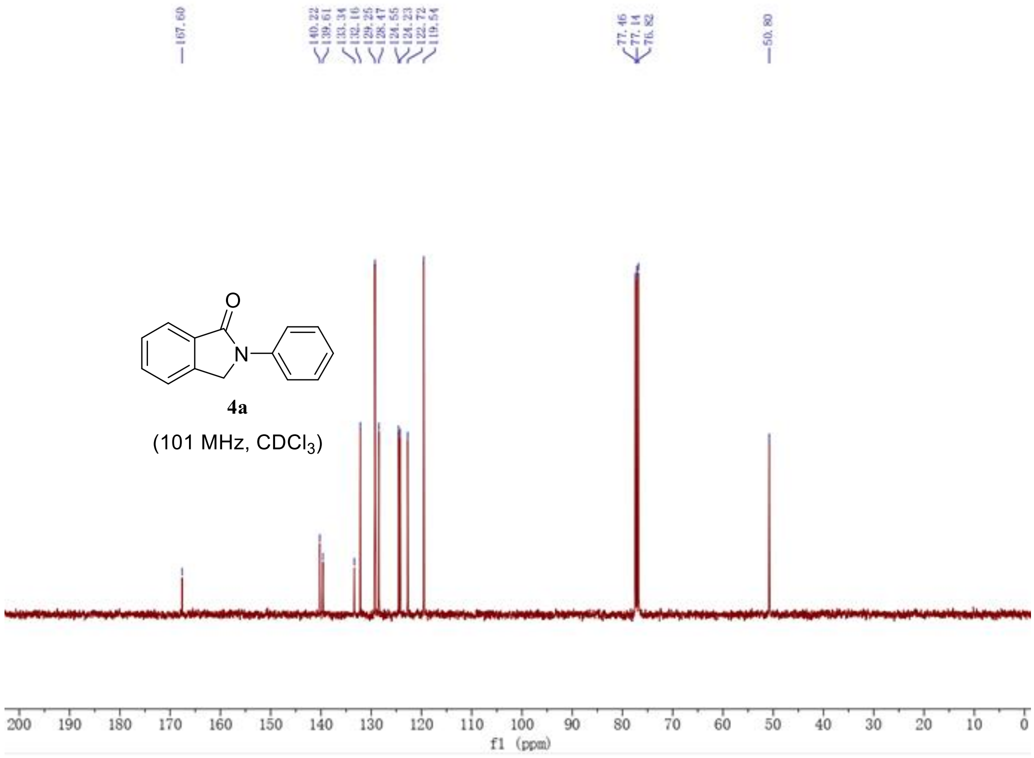

^{1}H NMR spectrum of 4b (400 MHz, $CDCl_3$)

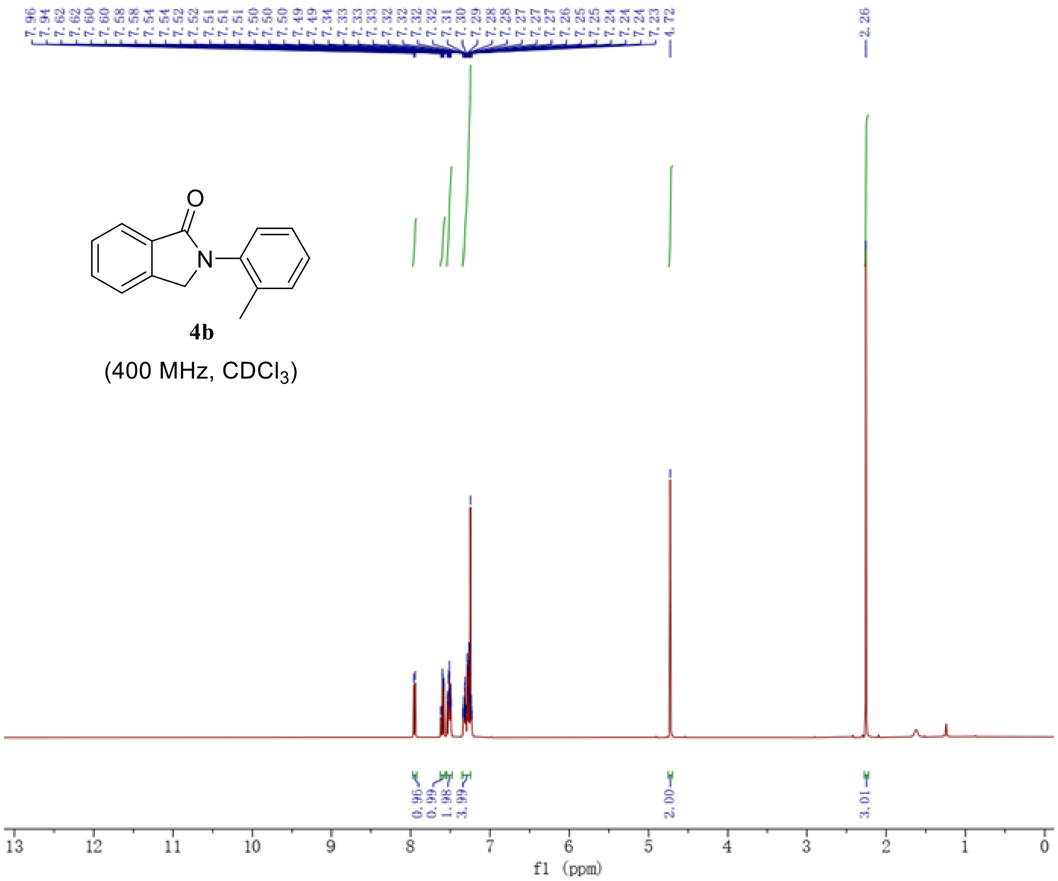

^{13}C NMR spectrum of 4b (101 MHz, $CDCl_3$)

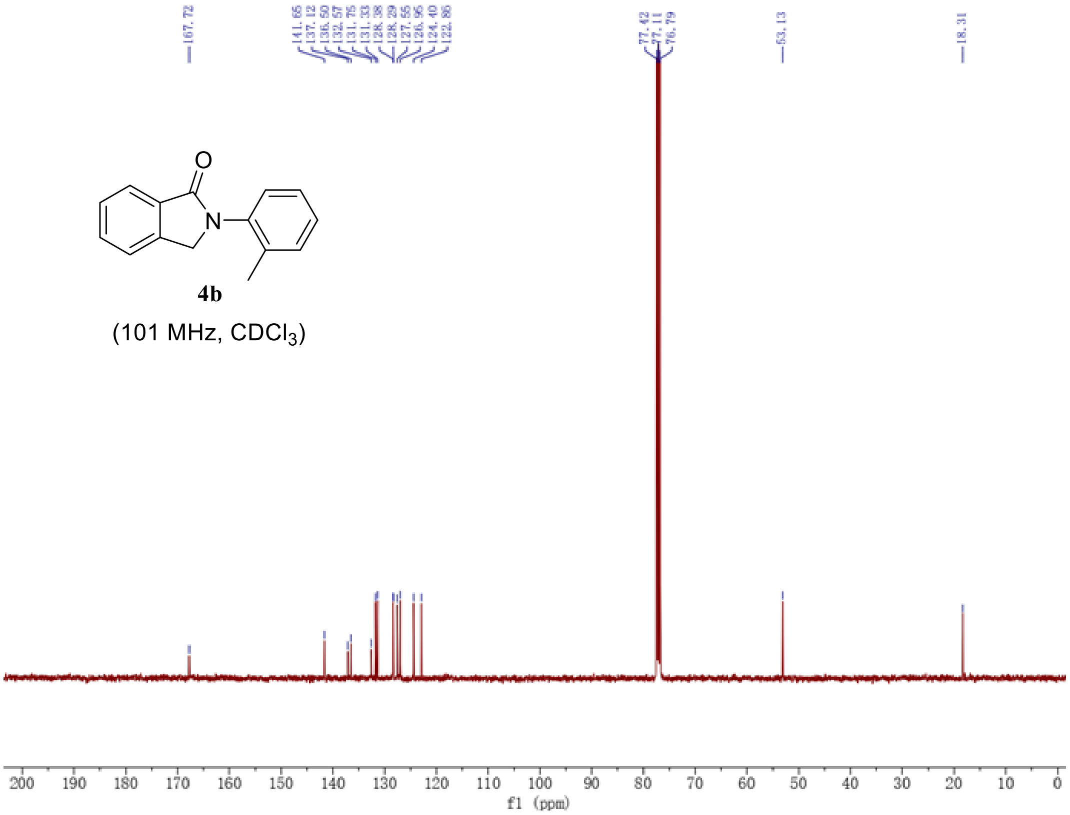

^{1}H NMR spectrum of 4c (400 MHz, $CDCl_3$)

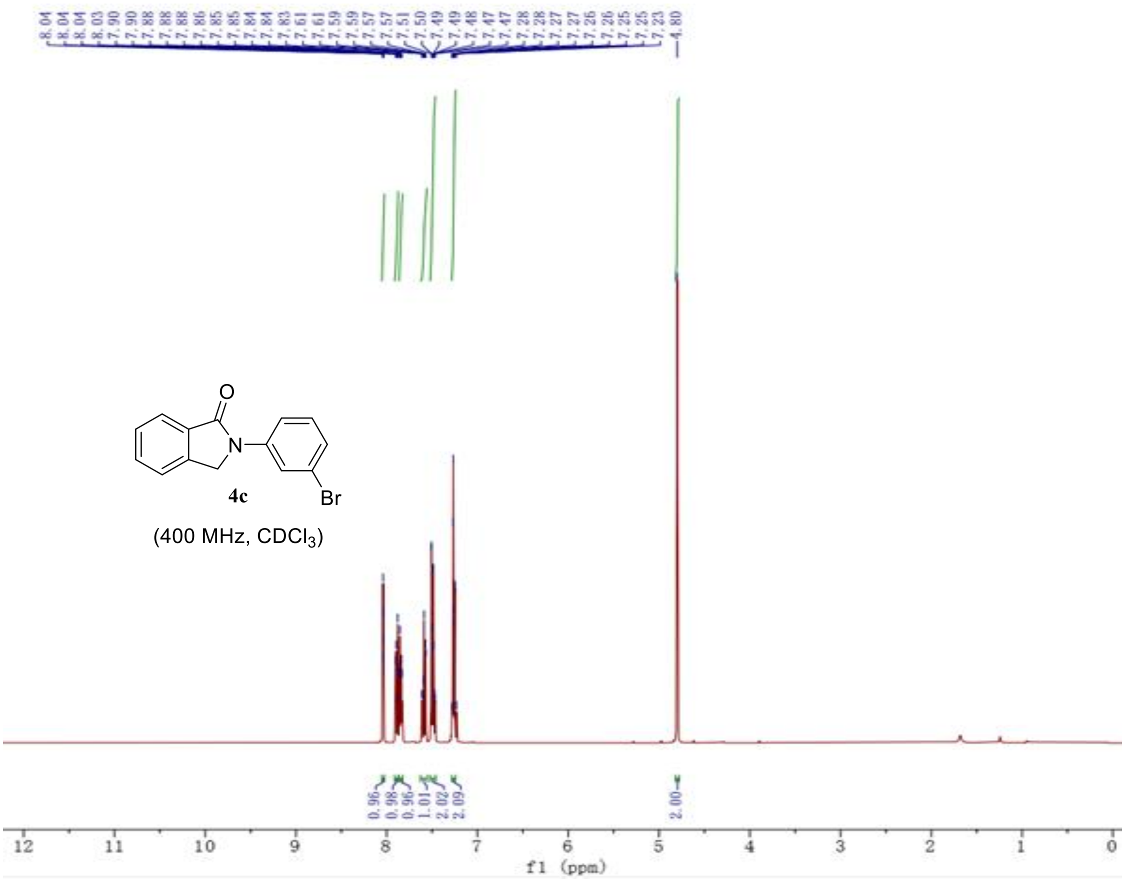

^{13}C NMR spectrum of 4c (101 MHz, $CDCl_3$)

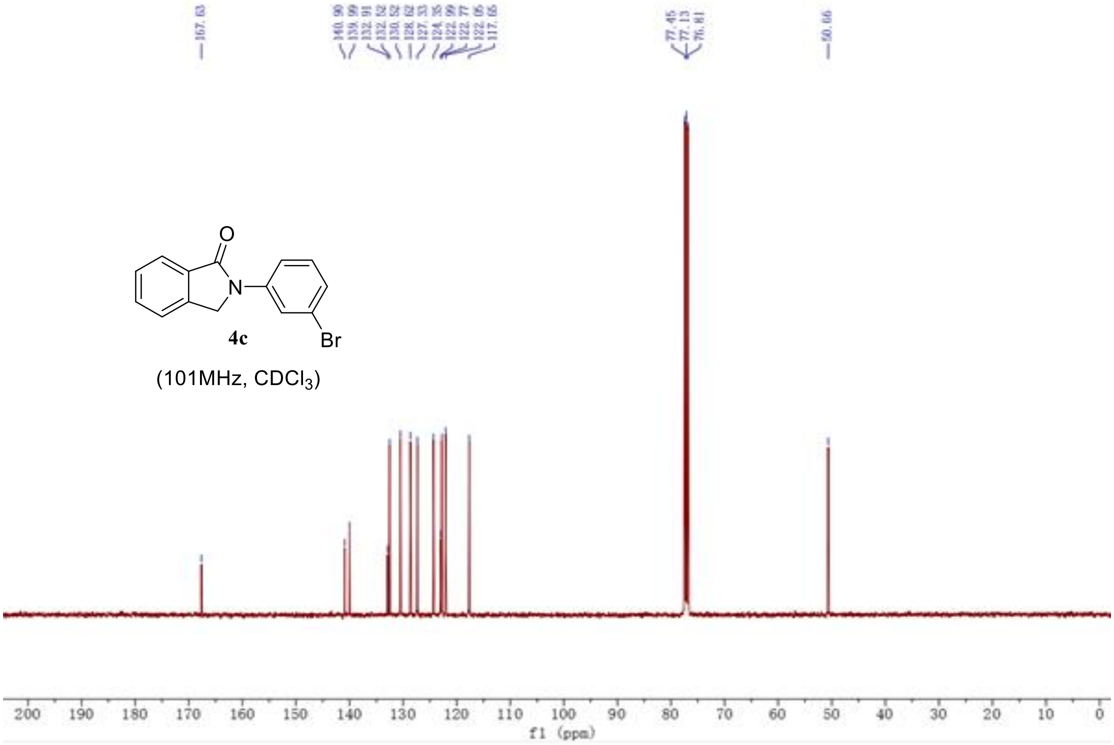

^{1}H NMR spectrum of 4d (400 MHz, $CDCl_3$)

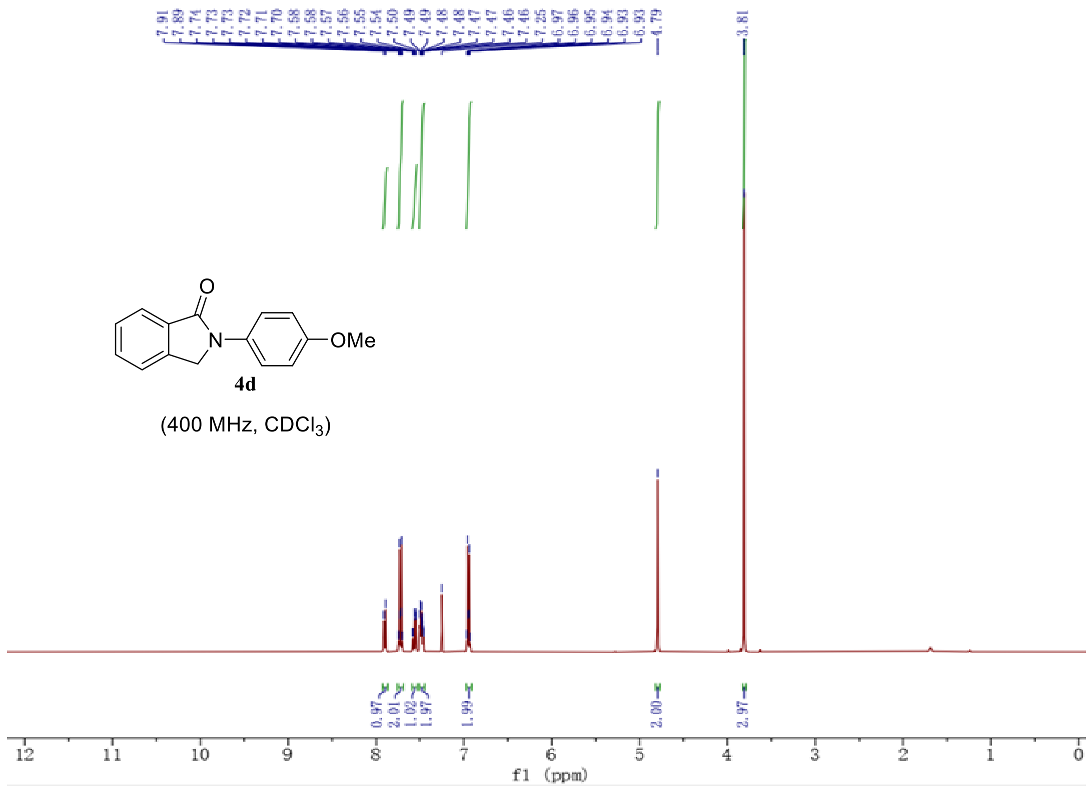

^{13}C NMR spectrum of 4d (101 MHz, $CDCl_3$)

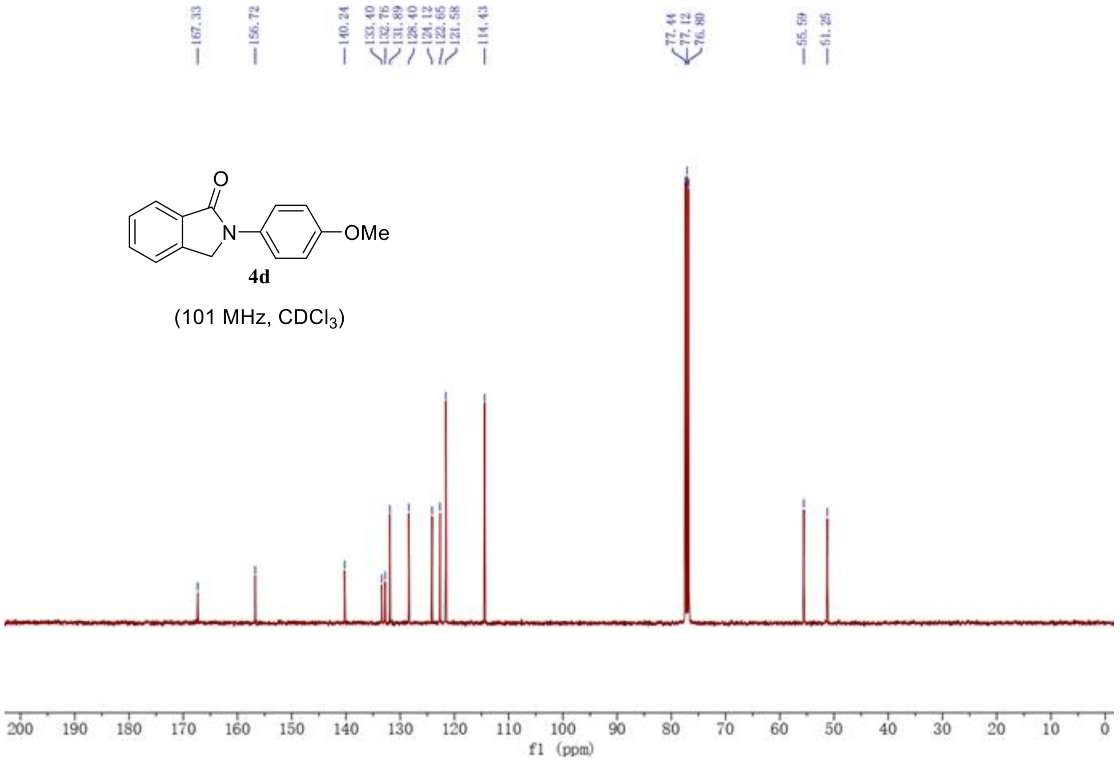

^{1}H NMR spectrum of 4e (400 MHz, $CDCl_3$)

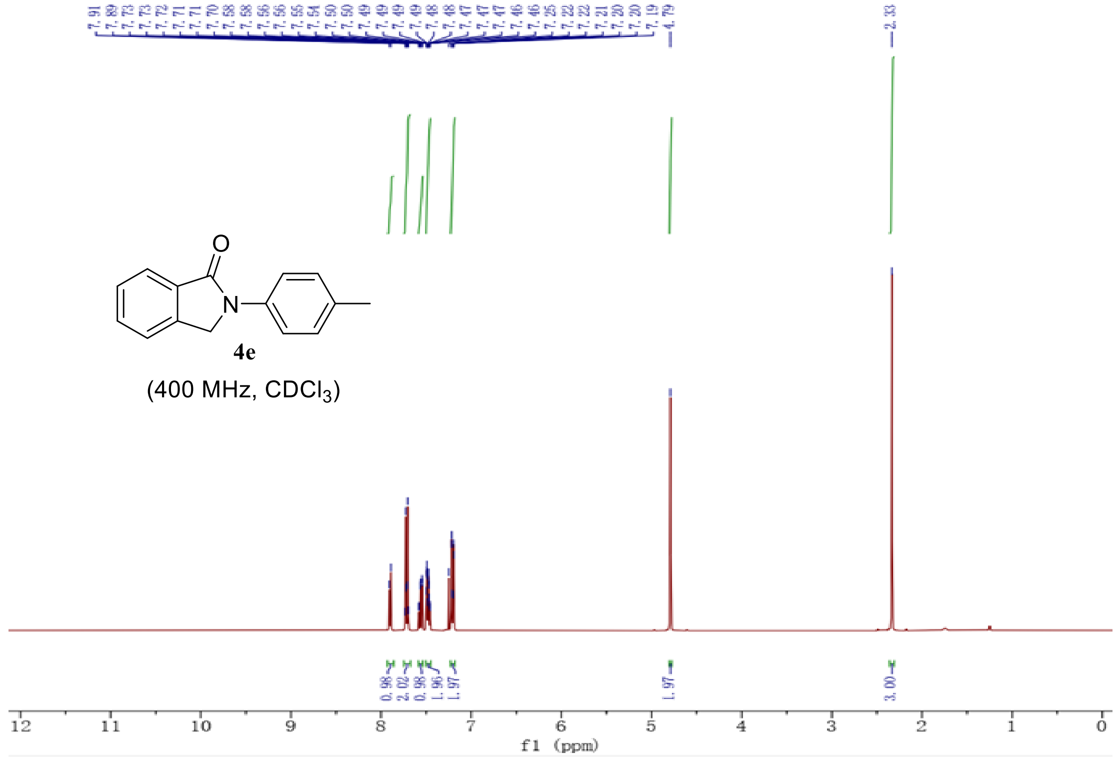

^{13}C NMR spectrum of 4e (101 MHz, $CDCl_3$)

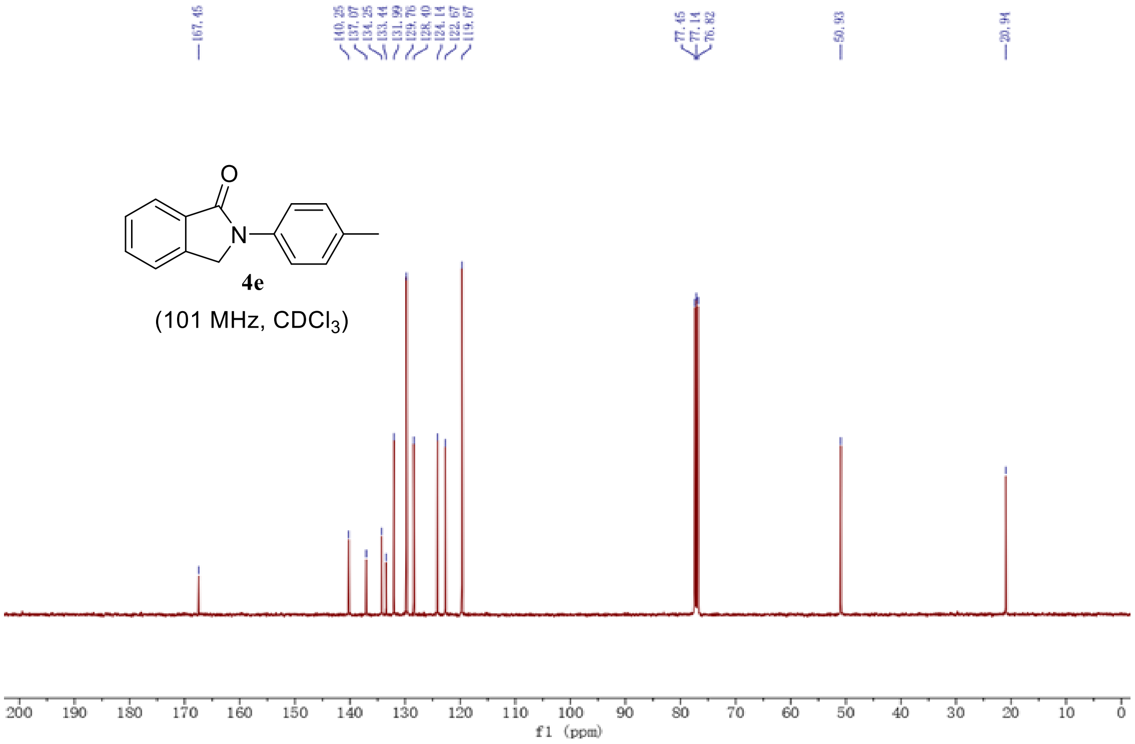

^{1}H NMR spectrum of 4f (400 MHz, $CDCl_3$)

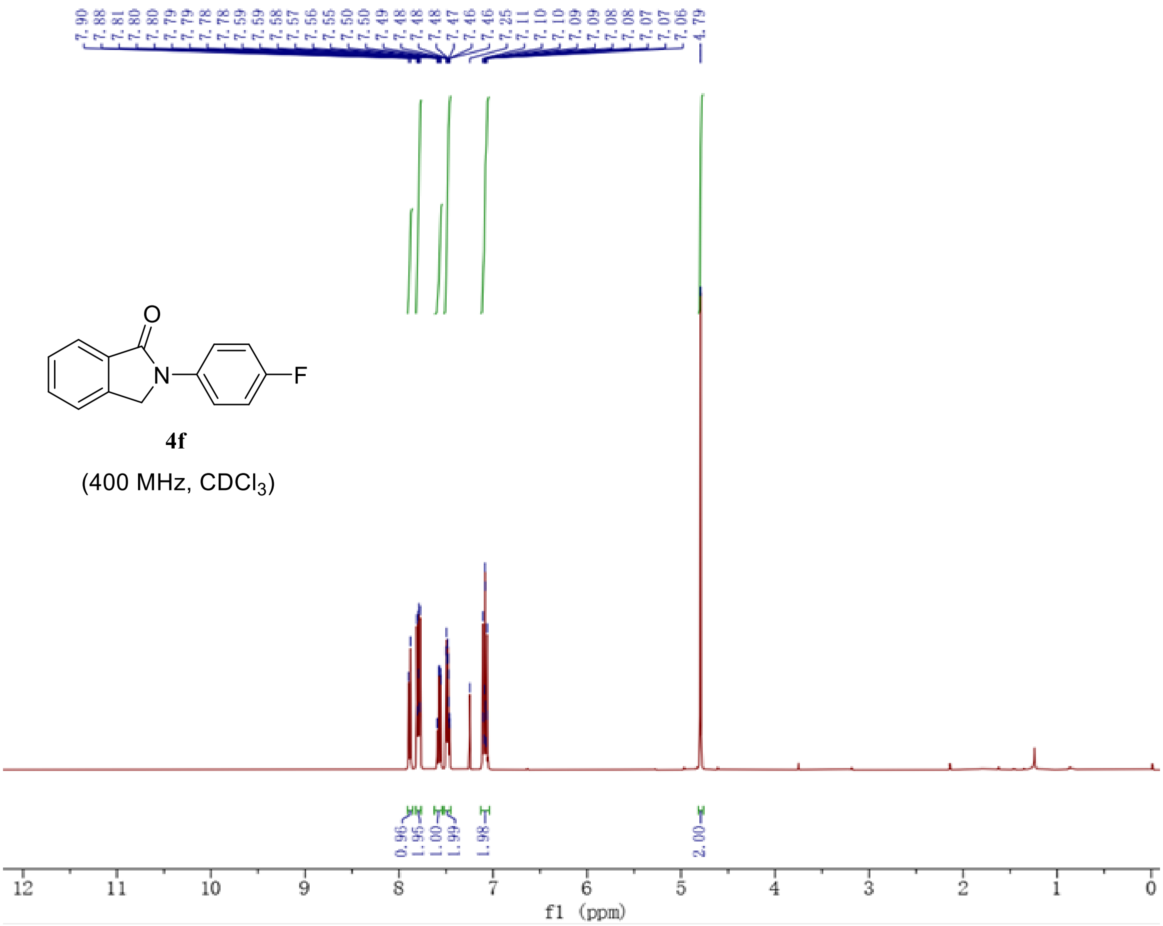

^{13}C NMR spectrum of 4f (101 MHz, $CDCl_3$)

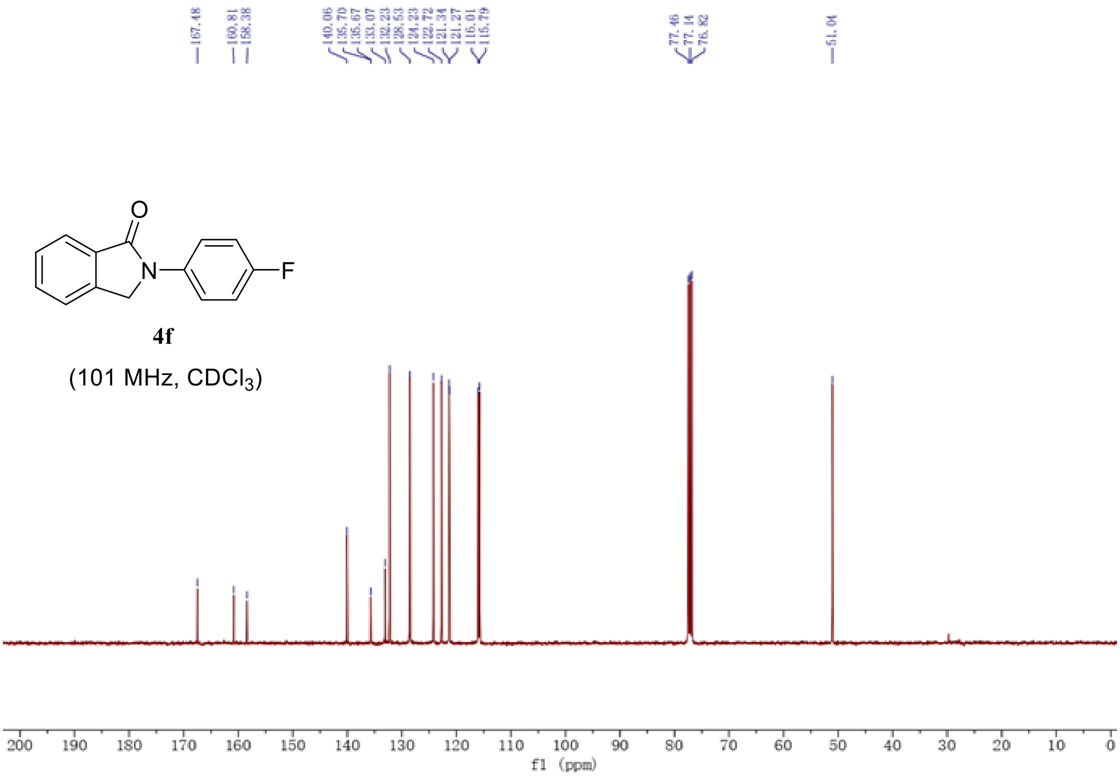

^{1}H NMR spectrum of 4g (400 MHz, $CDCl_3$)

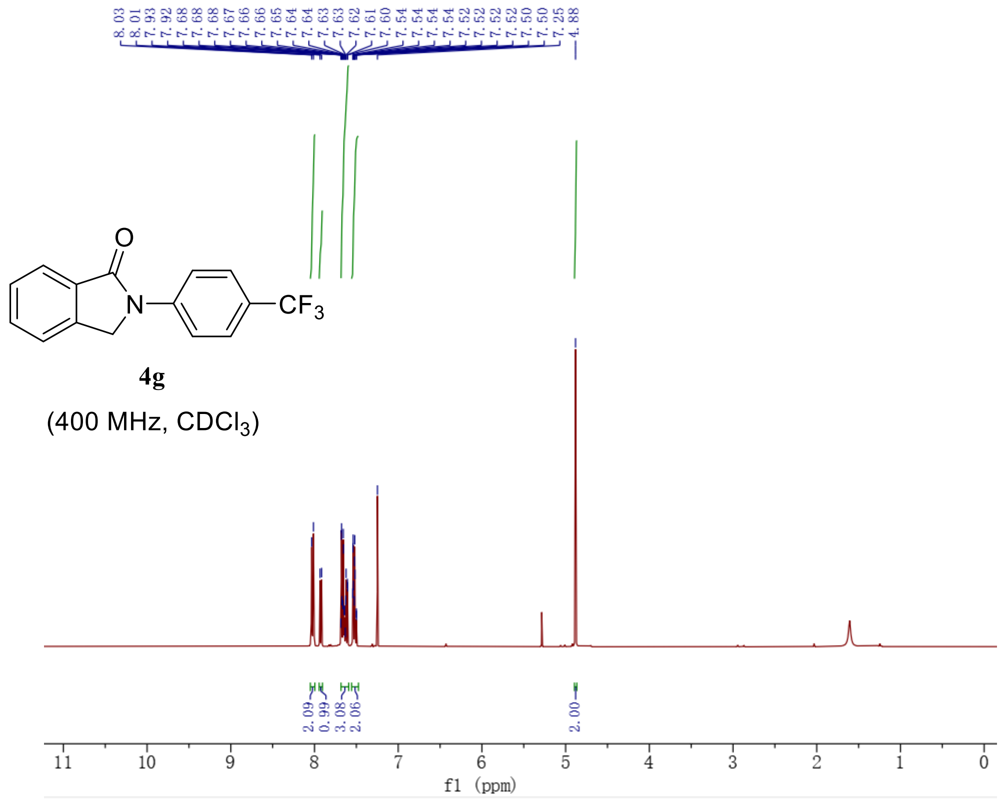

^{13}C NMR spectrum of 4g (101 MHz, $CDCl_3$)

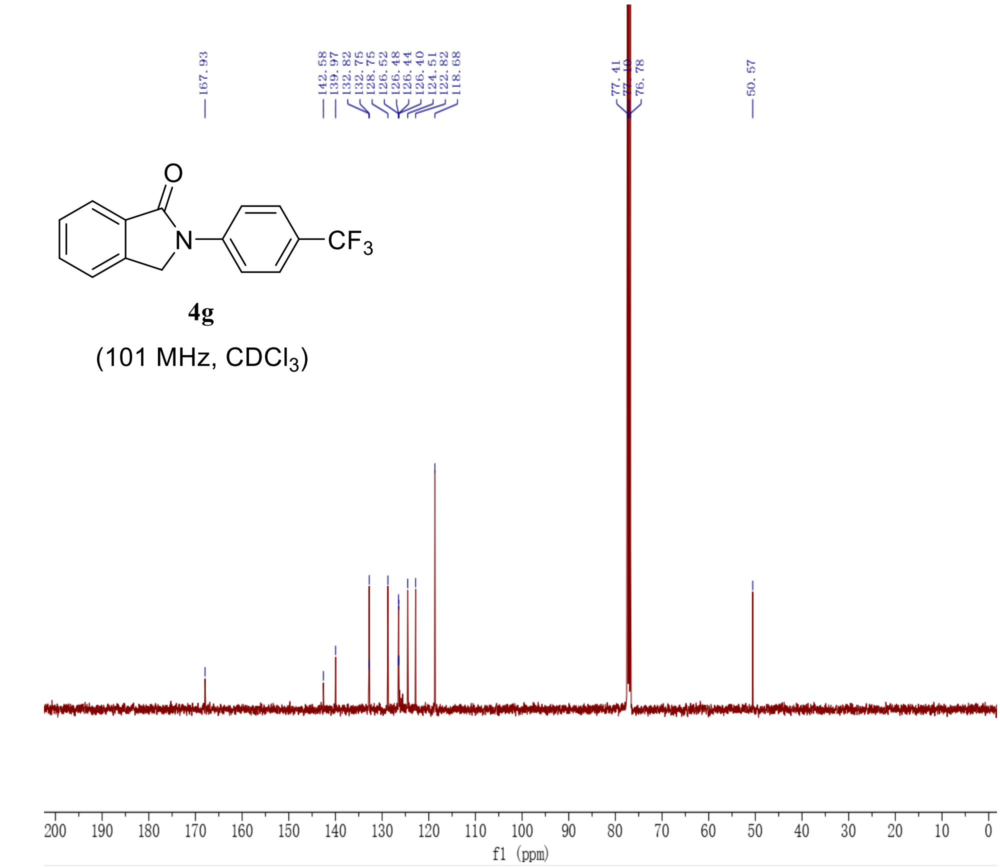

¹H NMR spectrum of 4h (400 MHz, CDCl₃)

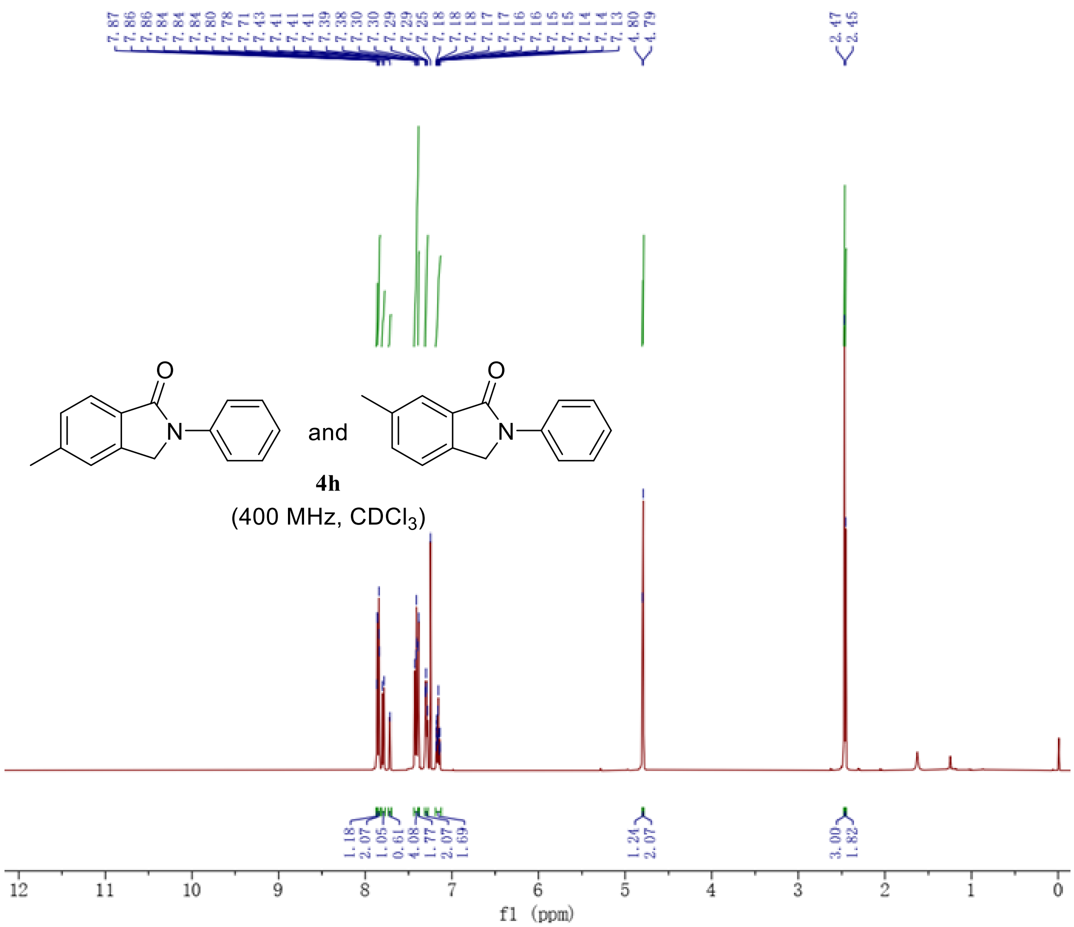

¹³C NMR spectrum of 4h (101 MHz, CDCl₃)

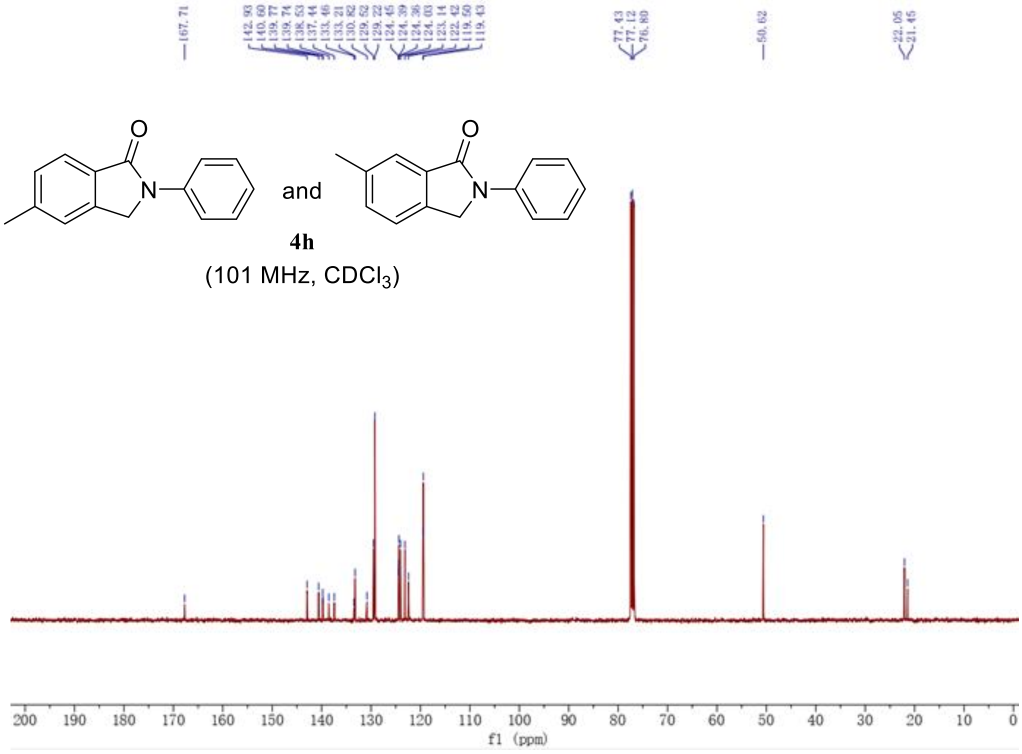

^{1}H NMR spectrum of 4i (400 MHz, $CDCl_3$)

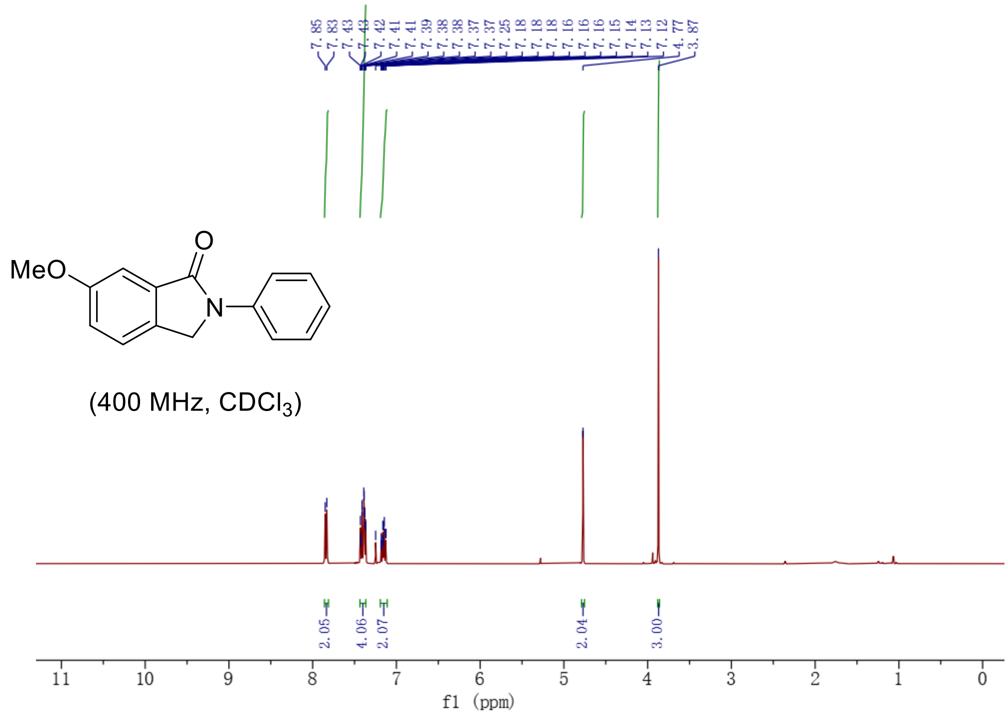

^{13}C NMR spectrum of 4i (101 MHz, $CDCl_3$)

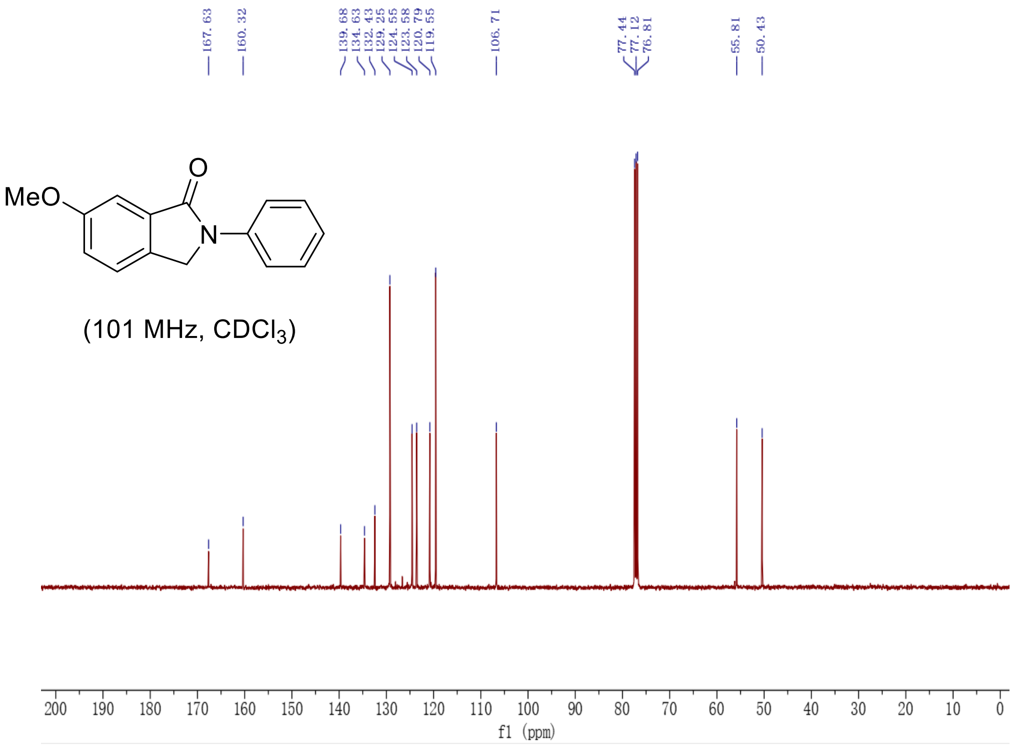

^{1}H NMR spectrum of 4i (400 MHz, $CDCl_3$)

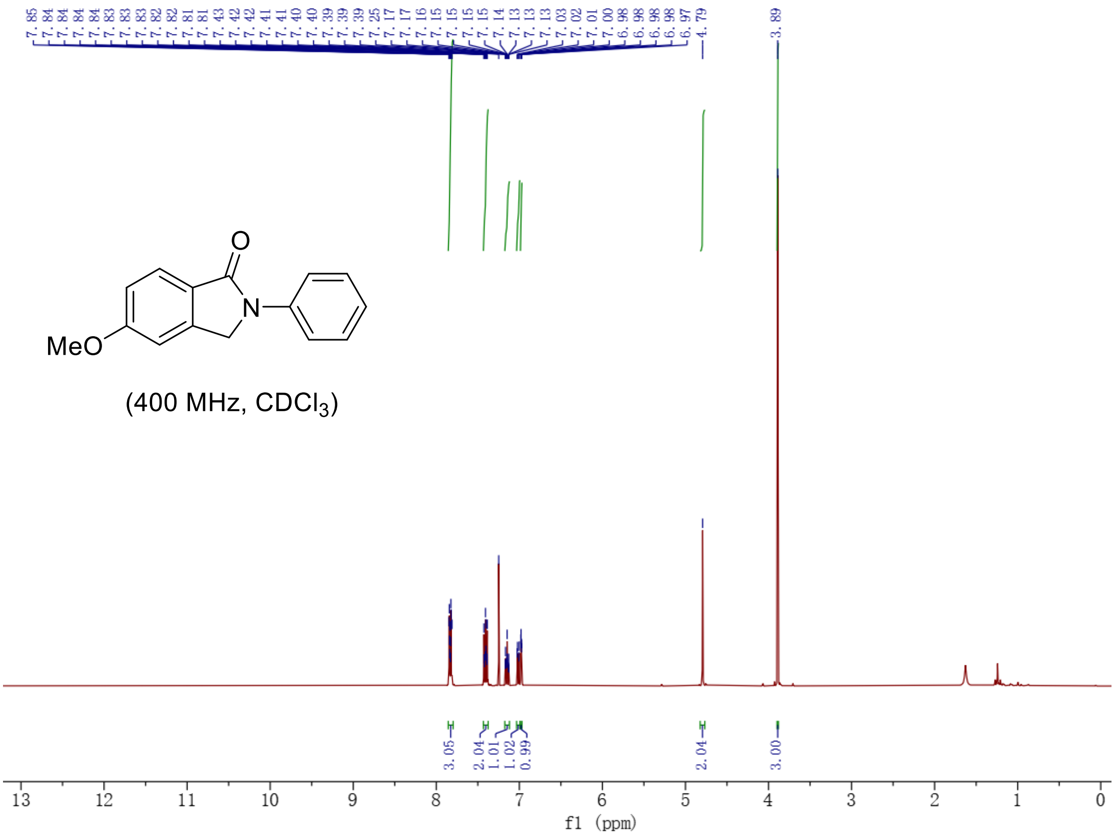

^{13}C NMR spectrum of 4i (101 MHz, $CDCl_3$)

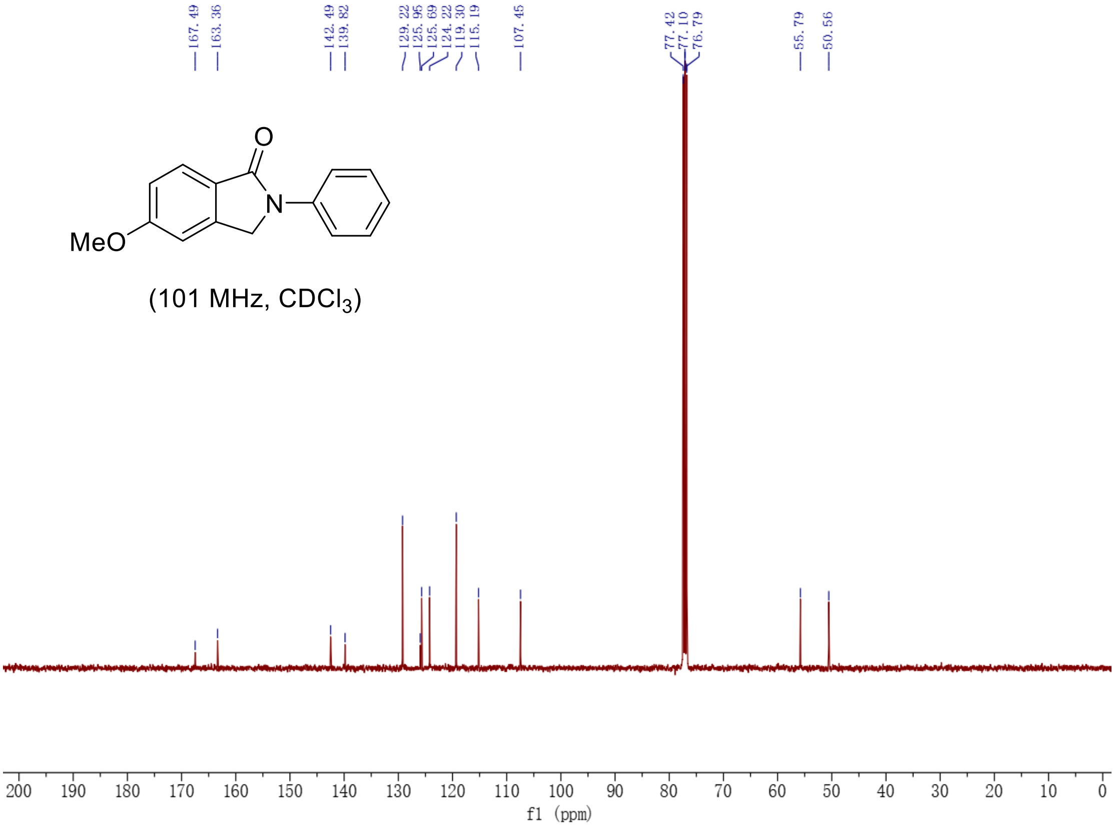

^{1}H NMR spectrum of 4j (400 MHz, $CDCl_3$)

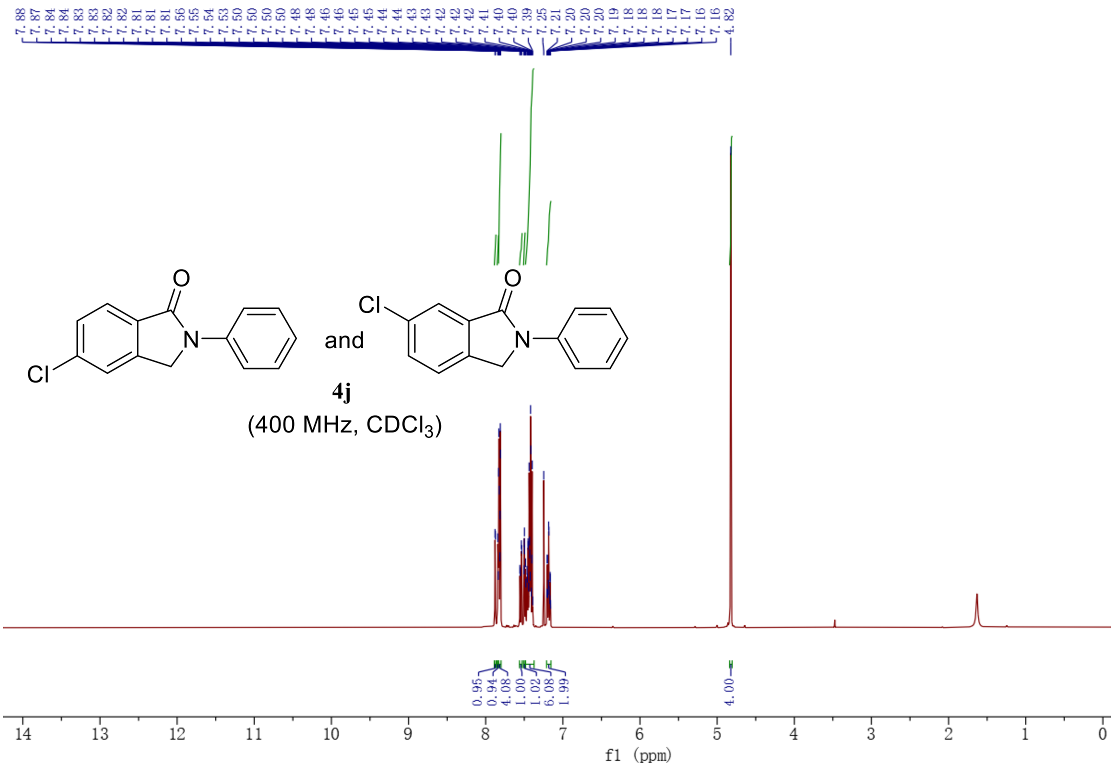

^{13}C NMR spectrum of 4j (101 MHz, $CDCl_3$)

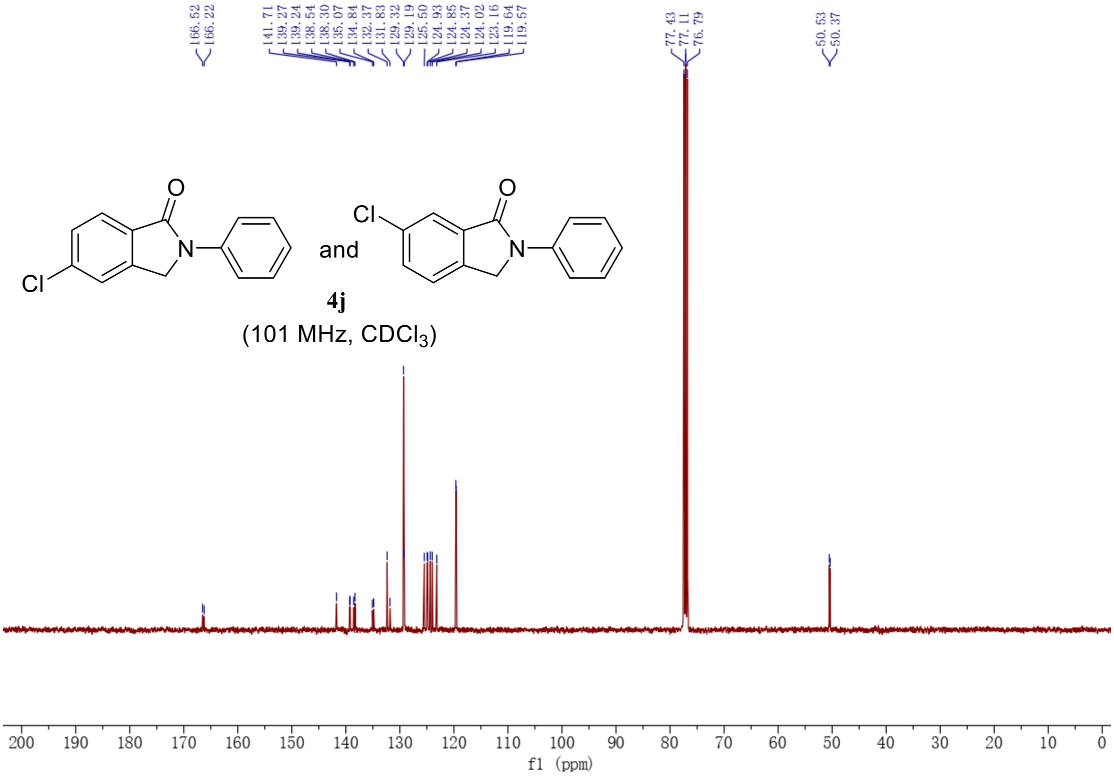

^{1}H NMR spectrum of 4k (400 MHz, $CDCl_3$)

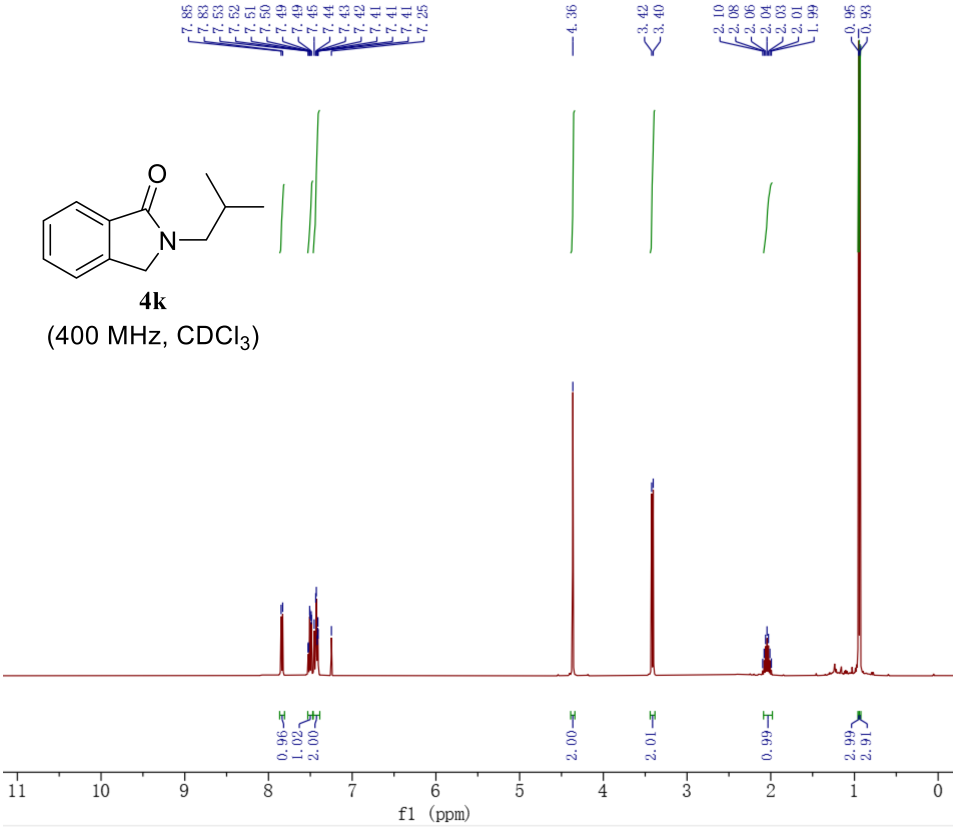

^{13}C NMR spectrum of 4k (101 MHz, $CDCl_3$)

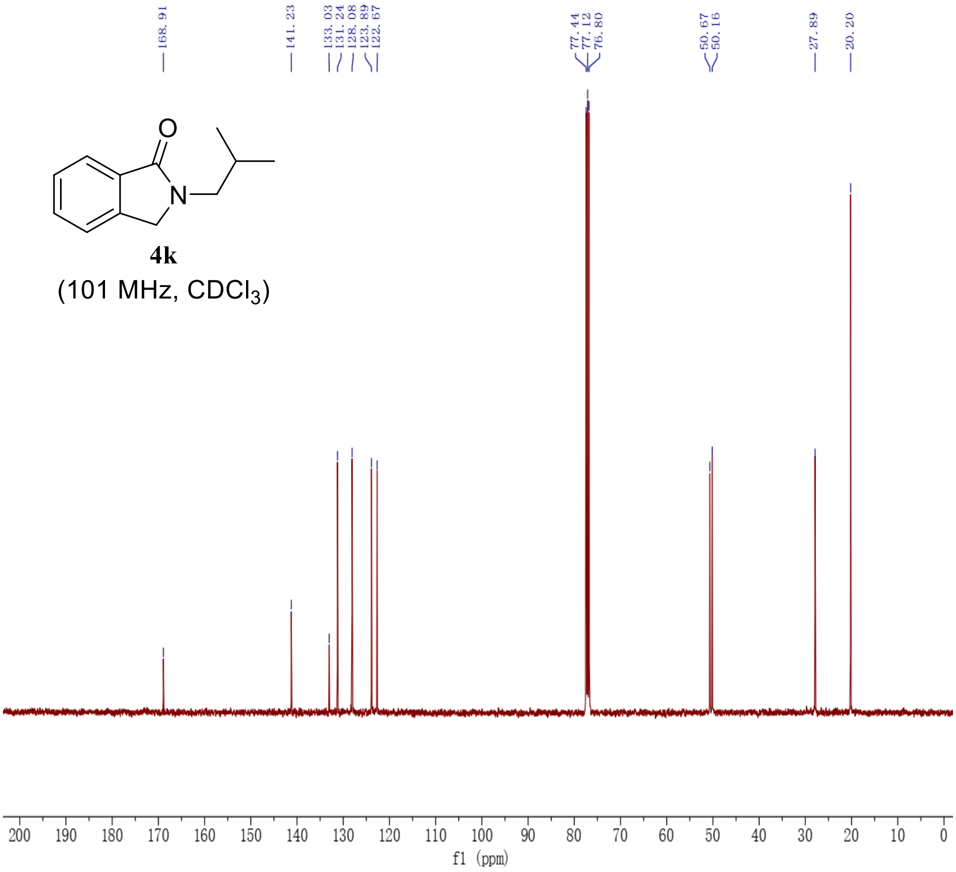

^{1}H NMR spectrum of 4l (400 MHz, $CDCl_3$)

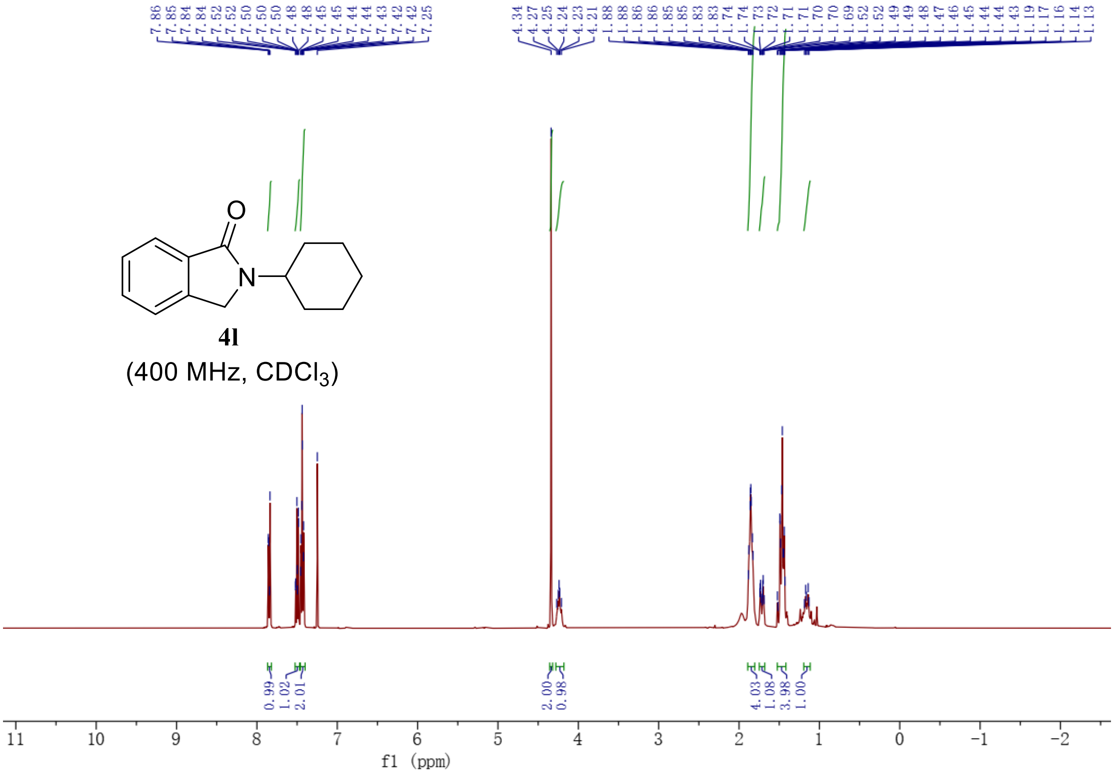

^{13}C NMR spectrum of 4l (101 MHz, $CDCl_3$)

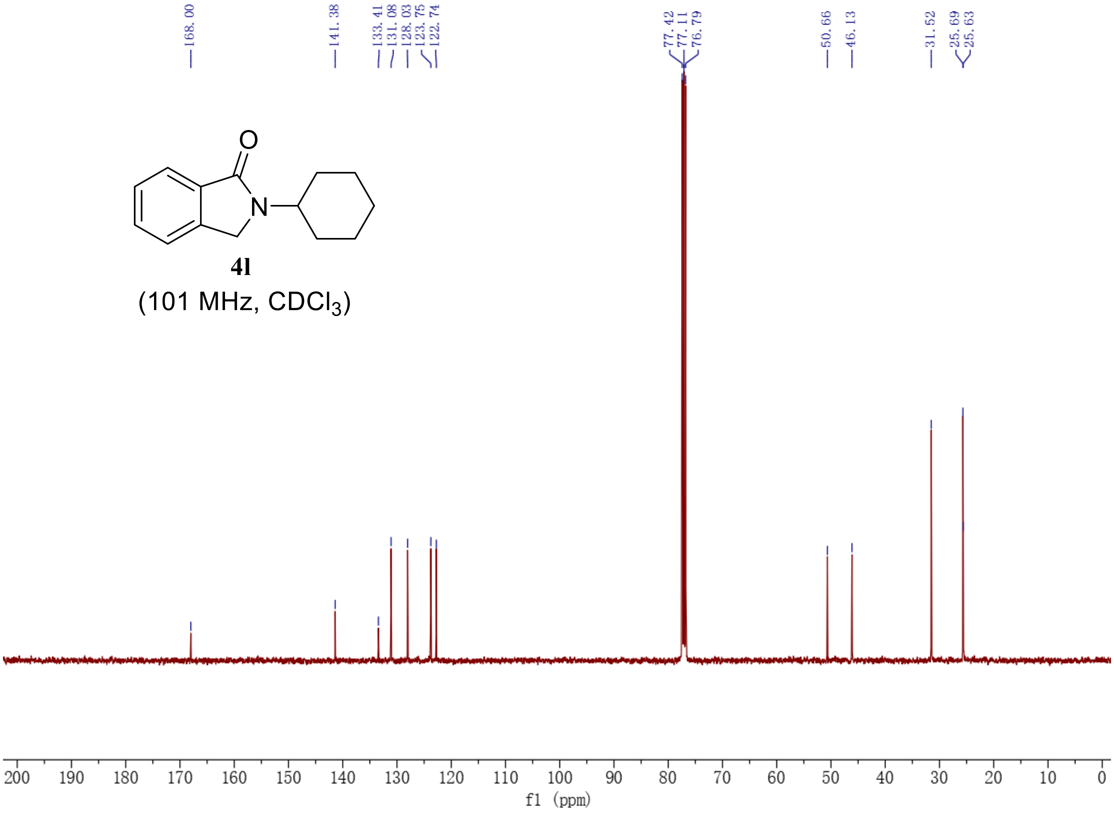

^{1}H NMR spectrum of 4m (400 MHz, CDCl$_3$)

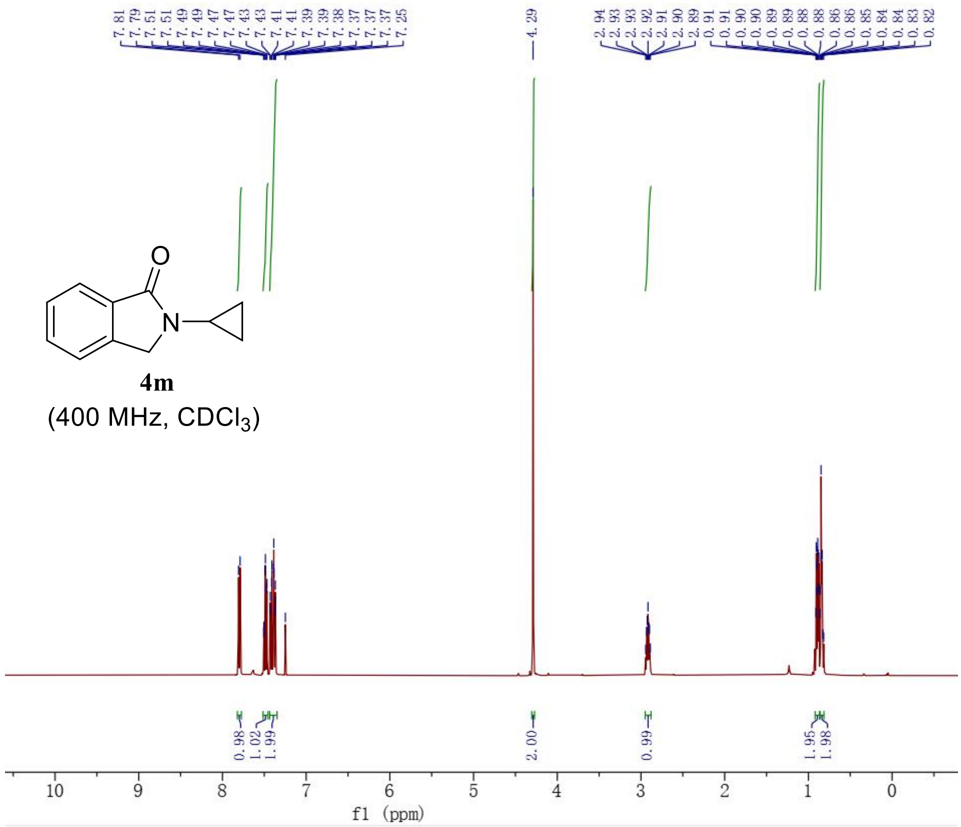

^{13}C NMR spectrum of 4m (101 MHz, CDCl$_3$)

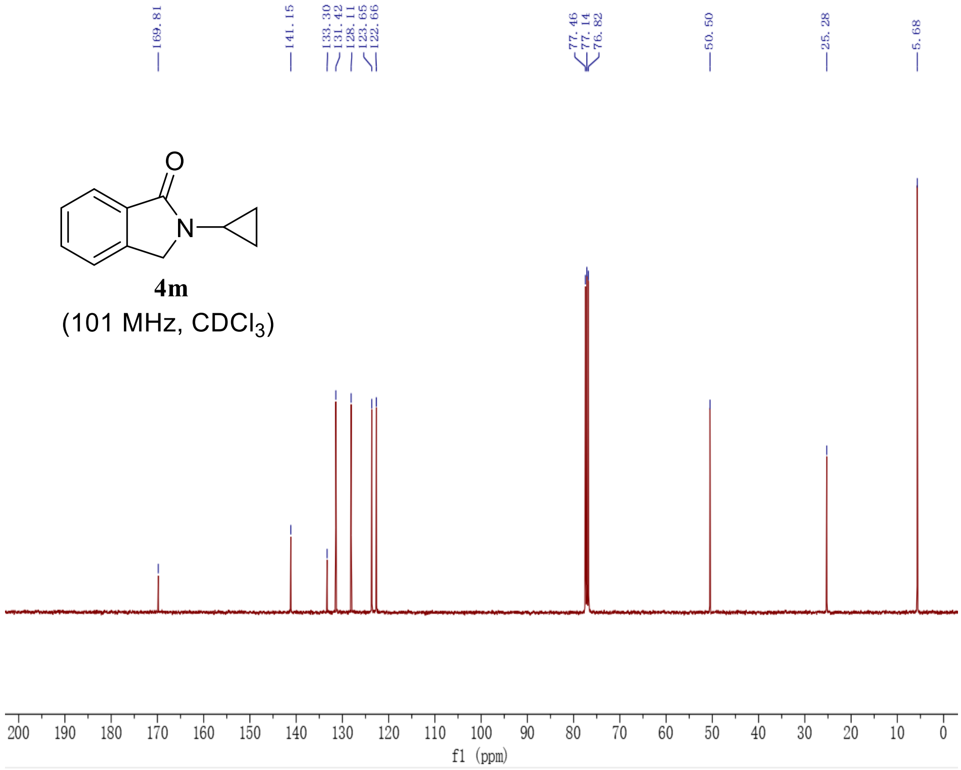

^{1}H NMR spectrum of 4n (400 MHz, $CDCl_3$)

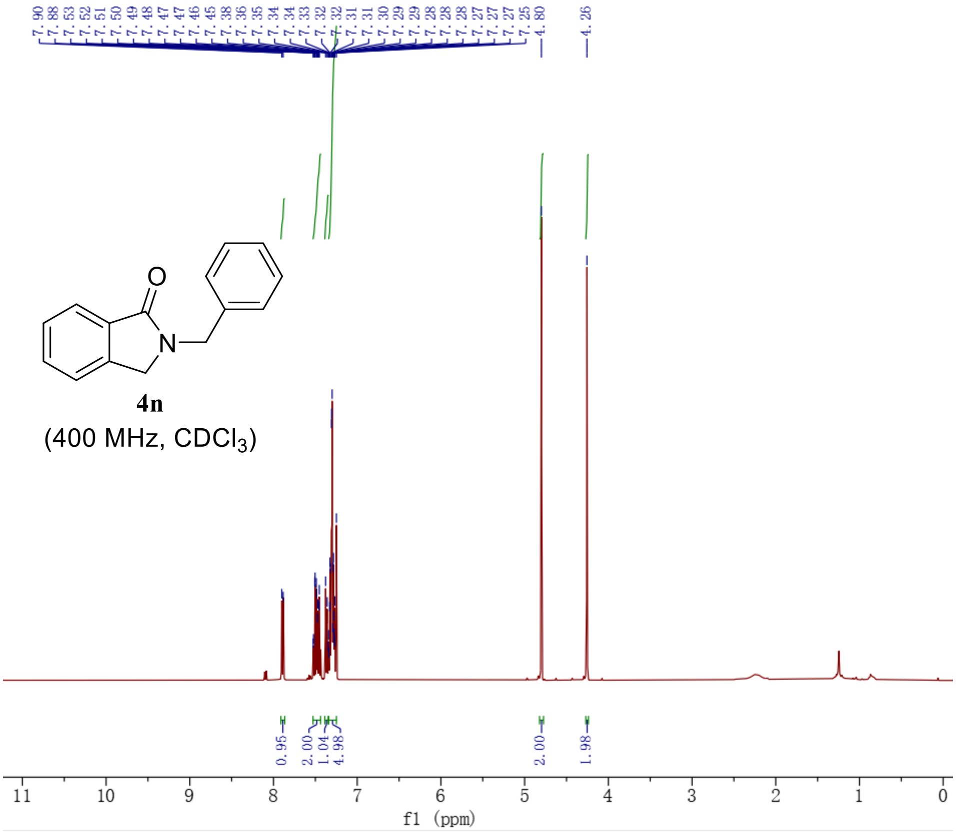

^{13}C NMR spectrum of 4n (101 MHz, $CDCl_3$)

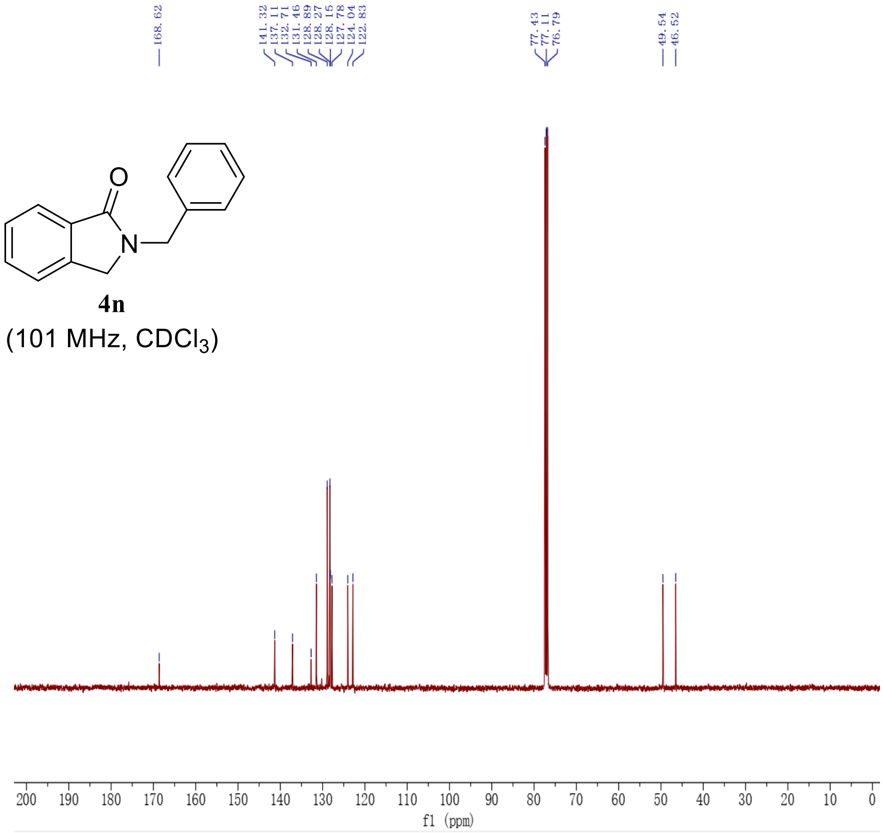

^{1}H NMR spectrum of 4o (400 MHz, $CDCl_3$)

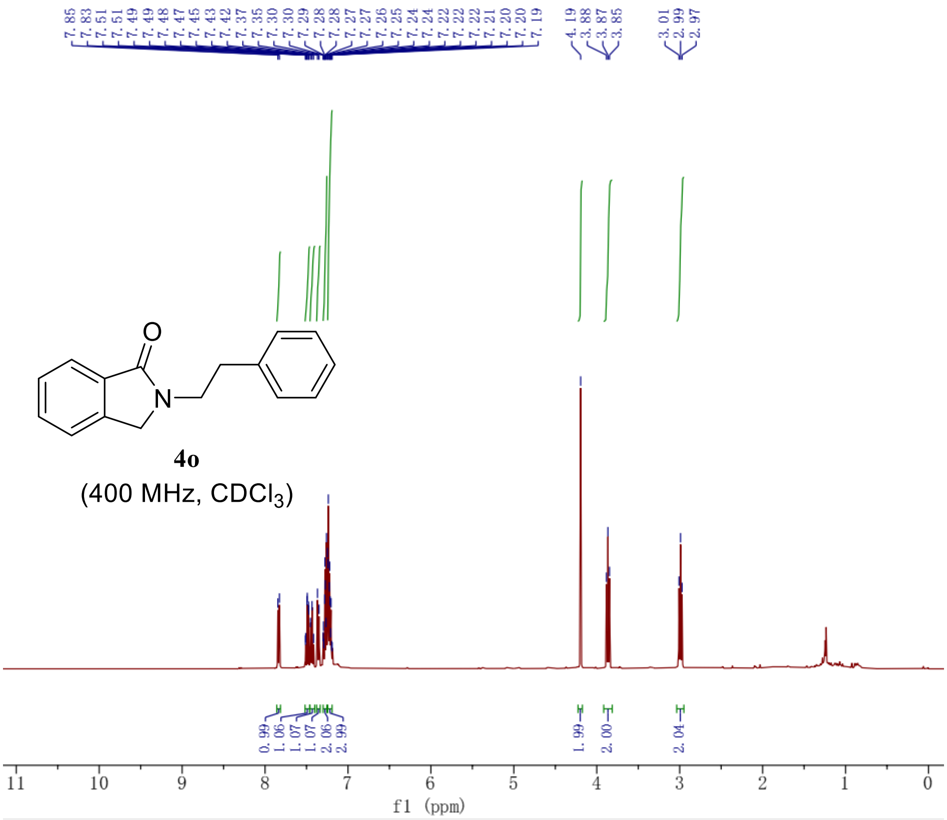

^{13}C NMR spectrum of 4o (101 MHz, $CDCl_3$)

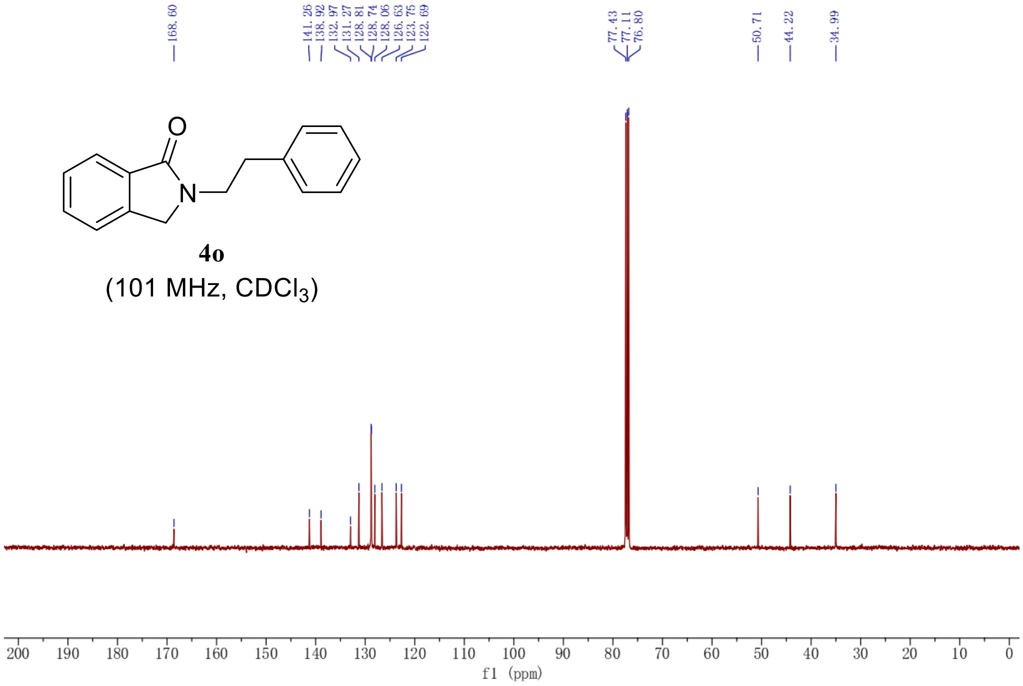

^{1}H NMR spectrum of BHT-OH (400 MHz, $CDCl_3$)

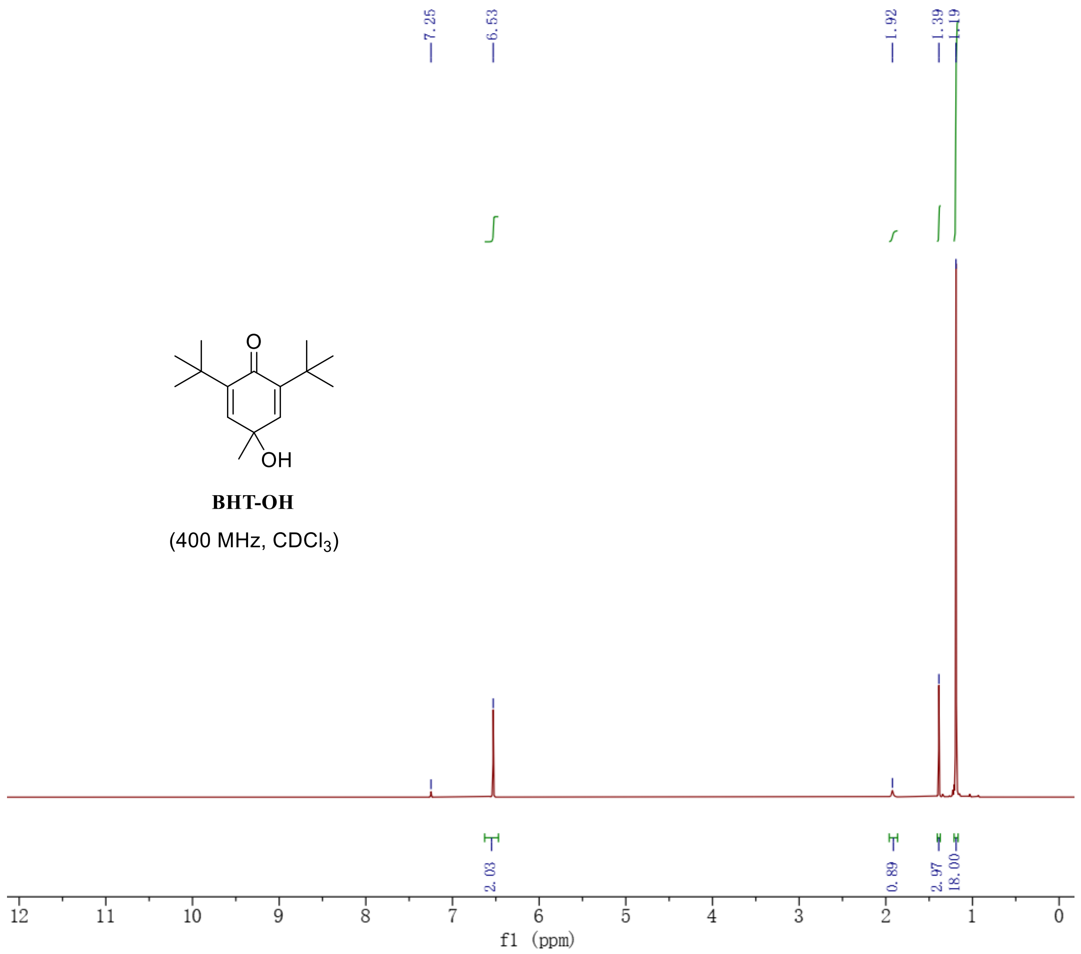

^{13}C NMR spectrum of BHT-OH (101 MHz, $CDCl_3$)

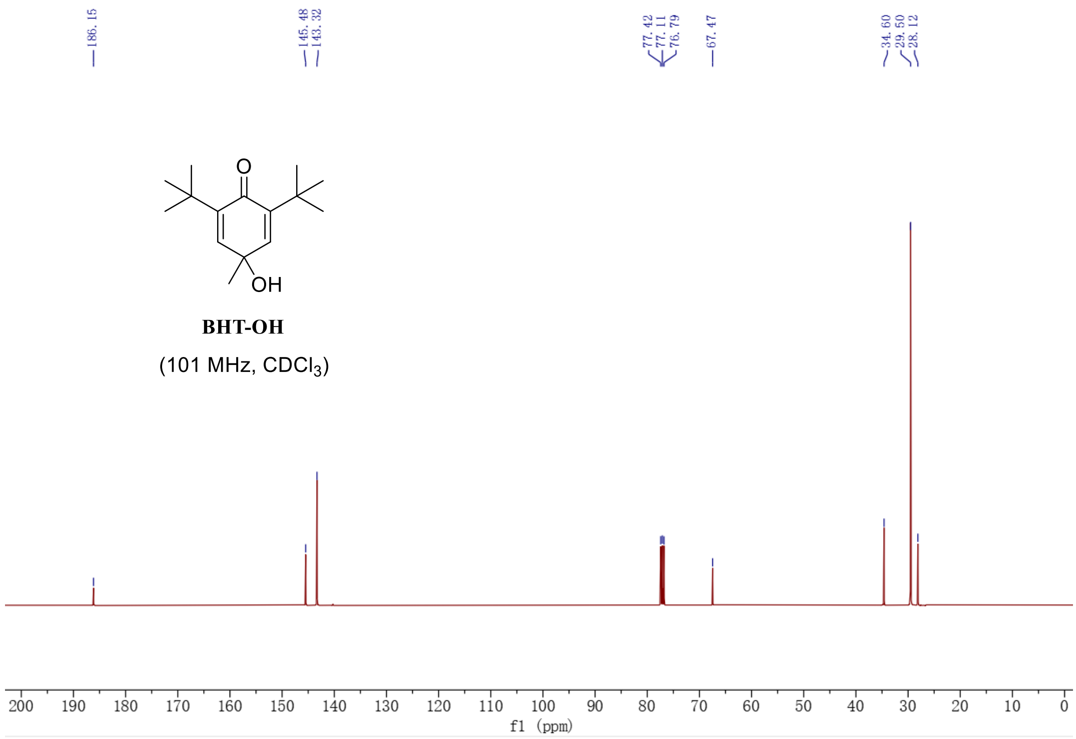

^{1}H NMR spectrum of S1 (400 MHz, $CDCl_3$)

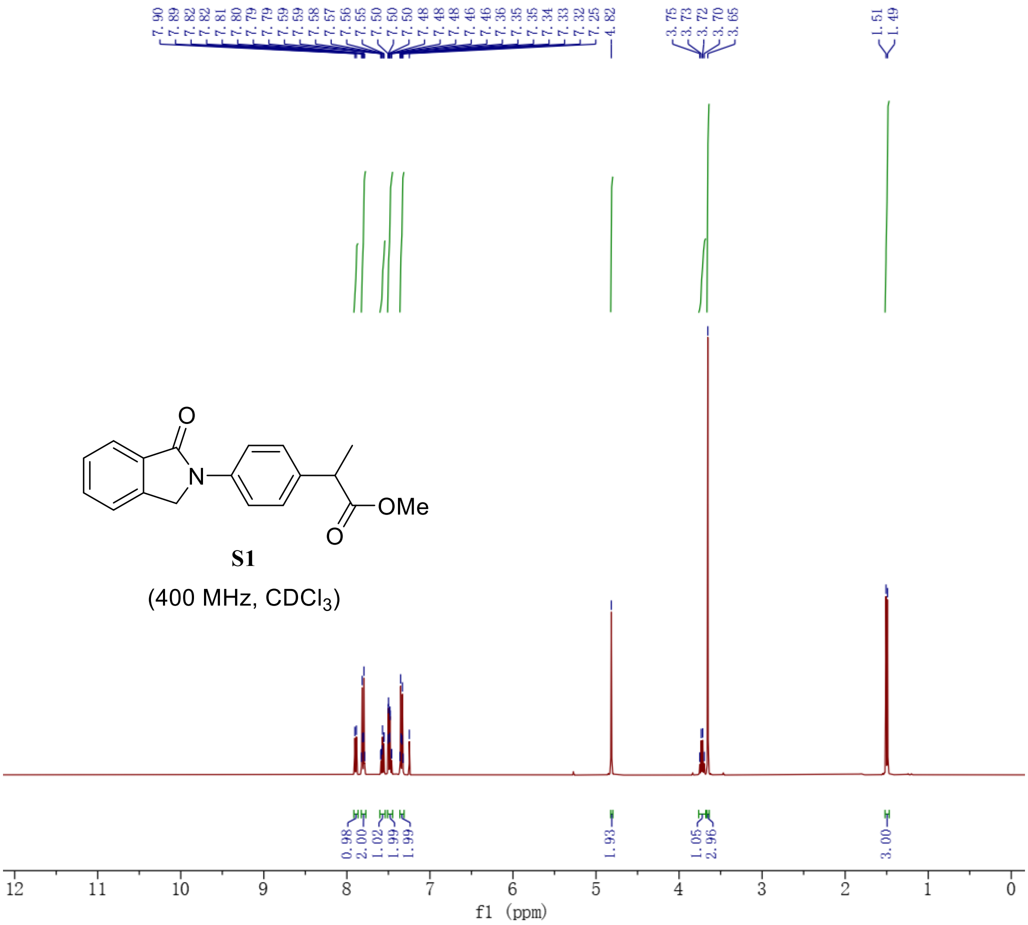

^{13}C NMR spectrum of S1 (101 MHz, $CDCl_3$)

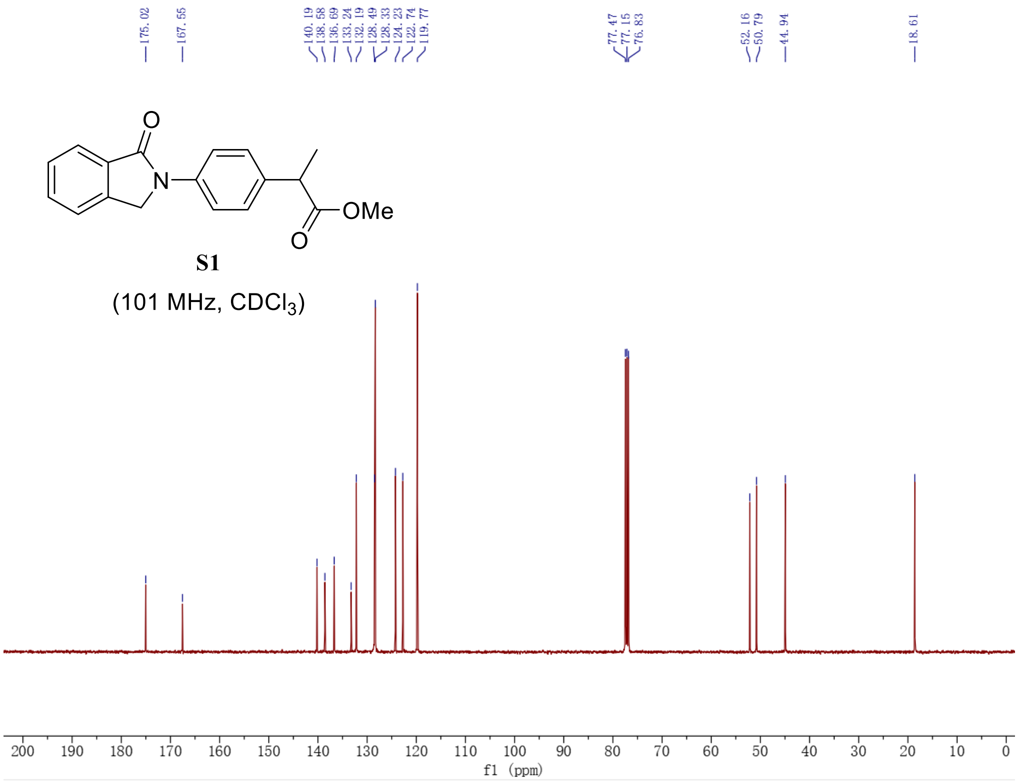

^{1}H NMR spectrum of indoprofen (400 MHz, $CDCl_3$)

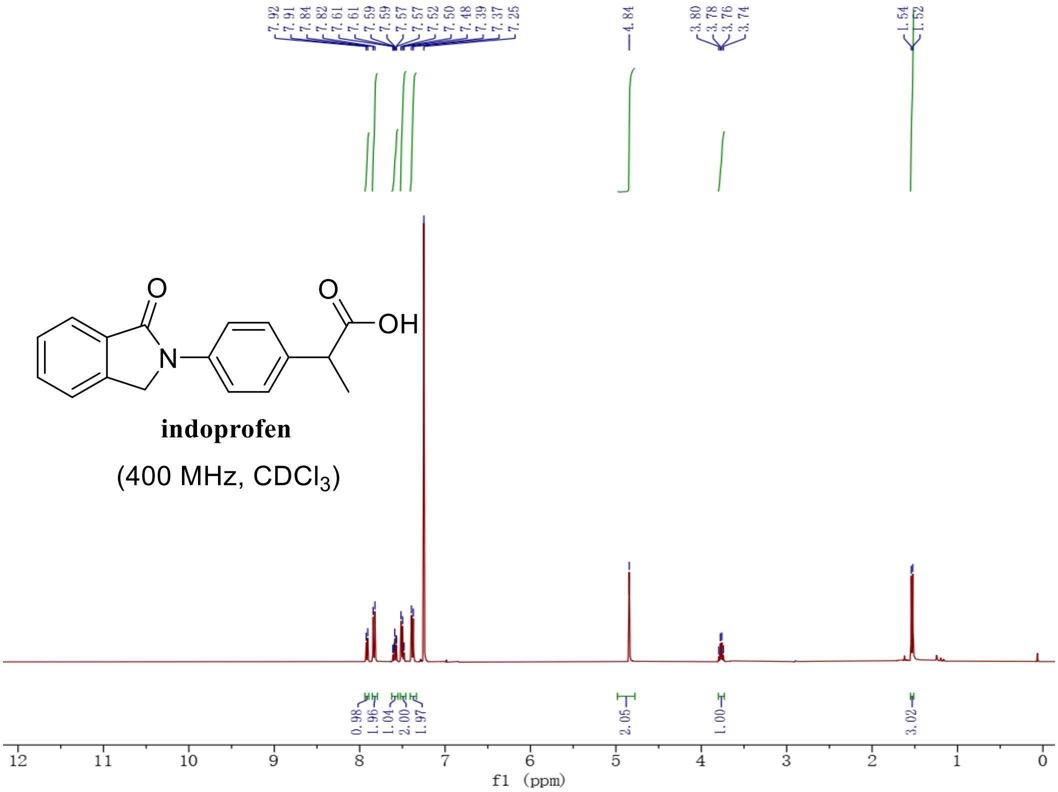

^{13}C NMR spectrum of indoprofen (101 MHz, $CDCl_3$)

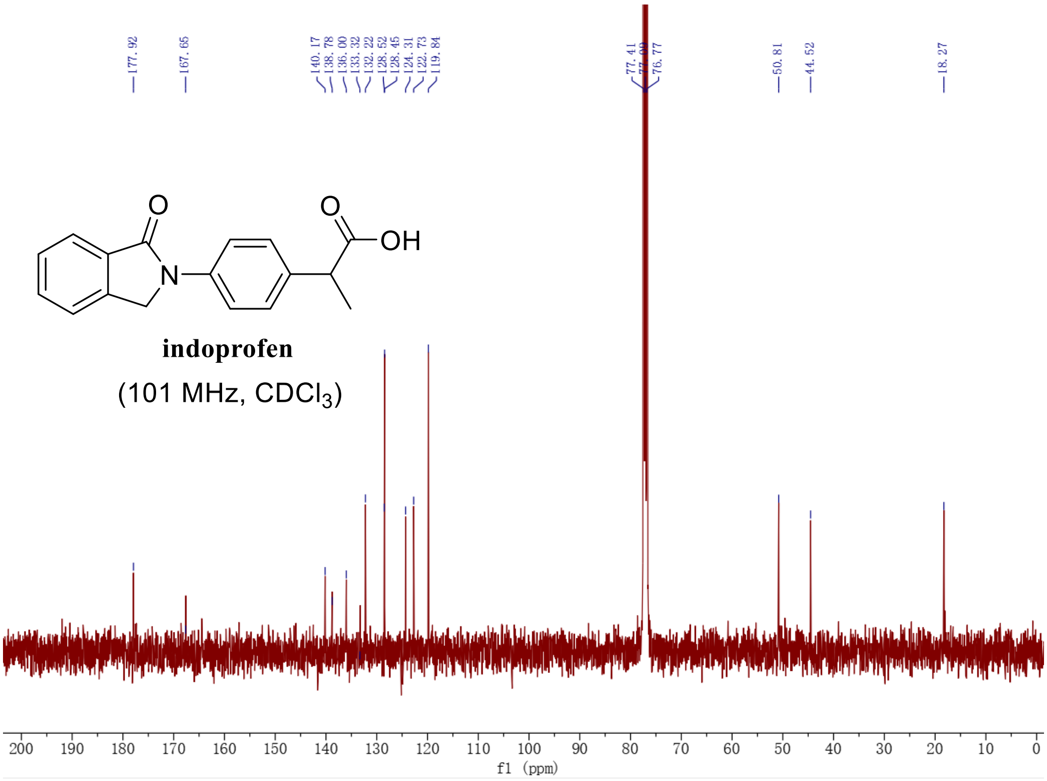

^{1}H NMR spectrum of 8-oxoberberine (400 MHz, $CDCl_3$)

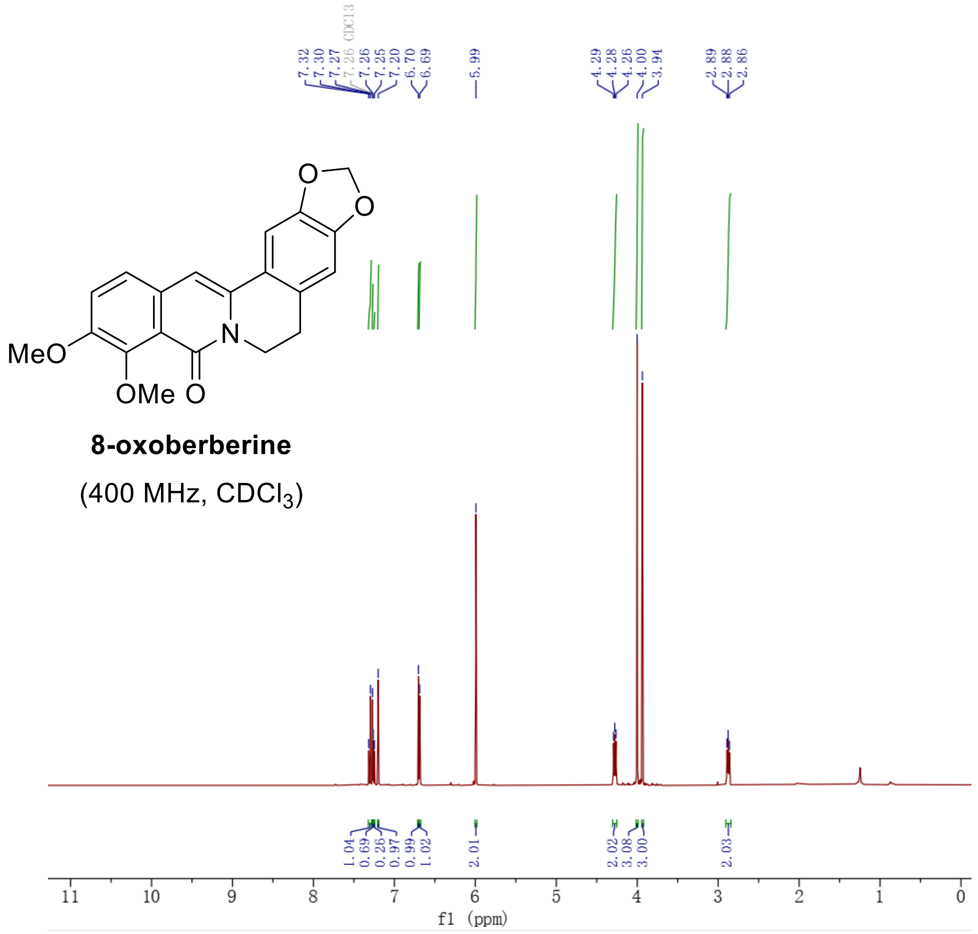

^{13}C NMR spectrum of 8-oxoberberine (101 MHz, $CDCl_3$)

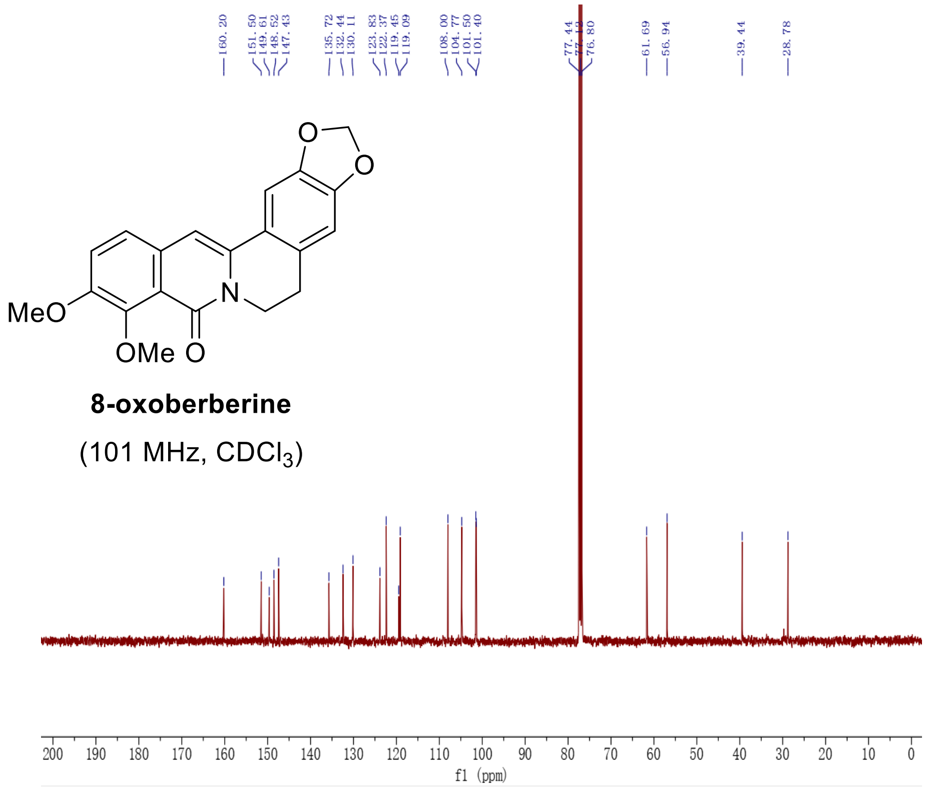

Supplement: Supplementary file 1 [file molecules-30-00743-s001.zip › molecules-3453973-supplementary.pdf]
